# Computer-guided design of novel nitrogen-based heterocyclic sphingosine-1-phosphate (S1P) activators as osteoanabolic agents

**DOI:** 10.17179/excli2024-7214

**Published:** 2024-05-27

**Authors:** Rattanawan Tangporncharoen, Chuleeporn Phanus-Umporn, Supaluk Prachayasittikul, Chanin Nantasenamat, Veda Prachayasittikul, Aungkura Supokawej

**Affiliations:** 1 Department of Clinical Microscopy, Faculty of Medical Technology, Mahidol University, Bangkok 10700, Thailand; 2 Department of Community Medical Technology, Faculty of Medical Technology, Mahidol University, Bangkok 10700, Thailand; 3 Center for Research Innovation and Biomedical Informatics, Faculty of Medical Technology, Mahidol University, Bangkok 10700, Thailand; 4 Streamlit Inc., San Francisco, CA 94121, USA

**Keywords:** Sphingosine-1-phosphate activators, Quantitative Structure-Activity Relationship, computer-aided drug design, osteoanabolic, quinoxalines, indoles

## Abstract

Osteoanabolic agents, or drugs that promote bone formation, have gained considerable attention for osteoporosis management due to their curative and preventive potentials. Sphingosine-1-phosphate receptor 2 (S1PR2) is an attractive drug target, in which its activation leads to osteogenesis-promoting effect. Nitrogen-containing heterocyclic scaffolds (i.e., quinoxaline and indole) are promising pharmacophores possessing diverse bioactivities and were reported as S1PR2 activators. Quantitative structure-activity relationship (QSAR) modeling is a computational approach well-known as a fundamental tool for facilitating successful drug development. This study demonstrates the discovery of new S1PR2 activators using computational-driven rational design. Herein, an original dataset of nitrogen-containing S1PR2 activators was collected from ChEMBL database. The retrieved dataset was separated into two datasets according to their core scaffolds (i.e., quinoxaline and indole). QSAR modeling was performed using multiple linear regression (MLR) algorithm to successfully obtain two models with good predictive performance. The constructed models also revealed key properties playing essential roles for potent S1PR2 activation, such as Van der Waals volume (R2v+ and E3v), mass (MATS5m and Km), electronegativity (H3e), and number of 5-membered rings (nR05). Subsequently, the constructed models were further employed to guide rational design and predict S1PR2 activating effects of an additional set of 752 structurally modified compounds. Most of the modified compounds were predicted to have higher potency than their parents, and a set of promising potent newly designed compounds was highlighted. Additionally, drug-likeness prediction was performed to reveal that most of the newly designed compounds are druggable compounds with possibility for further development.

## Introduction

Aging drives many degenerative conditions and is the most concerning health issue globally (United Nations Department of Economic Social Affairs, 2021[44]). Osteoporosis is a degenerative condition typically found among elders. It is initiated by an imbalanced bone remodeling leading to the loss of bone density and porous bone appearance with increased risk of fragility fractures (Akkawi and Zmerly, 2018[1]; Ensrud and Crandall, 2017[8]). Pharmacotherapeutics for osteoporosis are mainly aimed to increase bone mineral density either by preventing bone resorption (antiresorptive agents) or facilitating osteogenesis (osteoanabolic agents). The osteoanabolic agents have gained greater interest due to their healing-promoting effects (Kostenuik et al., 2023[16]). Several classes of anti-osteoporotic drugs are clinically available (i.e., bisphosphonates, selective estrogen receptor modulators, sclerostin inhibitors, and receptor activator of nuclear factor kappa-β ligand inhibitors (Akkawi and Zmerly, 2018[1]; Ensrud and Crandall, 2017[8]; Tu et al., 2018[43]). However, their adverse effects and complications are still concerning (Black et al., 2020[3]; Gilbert et al., 2022[12]; Wotton et al., 2019[49]). Accordingly, the discovery of novel osteoanabolic agents for osteoporosis management is of great interest.

Sphingosine-1-phosphate (S1P) plays crucial roles in regulating many biological systems including angiogenesis, inflammation, neurogenesis (Mendelson et al., 2014[21]; Takuwa et al., 2012[42]), osteogenesis (Cao et al., 2019[4]; Sartawi et al., 2017[38]), and bone remodeling (Grewe et al., 2022[13]). Therefore, their receptors are attractive drug targets for several clinical indications (Baumruker et al., 2007[2]; Yazdi et al., 2020[50]). Among other subtypes, S1P receptor 2 (S1PR2) was noted for its role in bone remodeling. An activation of S1PR2 was reported to induce the expression of a key transcriptional factor (RUNX2) playing a role in osteoblast differentiation (Higashi et al., 2016[15]; Sato et al., 2012[39]), increase osteoblast differentiation markers at mRNA level (Matsuzaki et al., 2022[19]), and improve bone structural parameters in several *in vivo* models (Matsuzaki et al., 2022[19]; Weske et al., 2018[46], 2019[47]). Accordingly, targeting the activation of S1PR2 is a promising strategy for managing bone diseases.

Nitrogen-based heterocyclic compounds (i.e., quinoxaline and indole) are promising pharmacophores for drug discovery due to their various bioactivities. Quinoxalines were reported as antimicrobial, anticancer, anti-inflammatory and antidepressant agents (Pereira et al., 2015[25]). Similarly, indoles were noted for their anti-inflammatory, anticancer, antimicrobial, antidiabetic, and antioxidant activities (Sravanthi and Manju, 2016[41]). Notably, these nitrogen-containing compounds were reported to take parts in bone remodeling via modulating the differentiations and functions of both osteoclast and osteoblast (Ha et al., 2021[14]; Yu et al., 2015[51]; Zhou et al., 2021[52]). Therefore, these pharmacophores are attractive scaffolds for the discovery of new anti-osteoporotic drugs.

Computational (*in silico*) approaches have been employed as facilitating tools in drug development for decades (Prachayasittikul et al., 2015[29]) due to their benefits in reducing late-stage failures, increasing success rate, and saving time (Vemula et al., 2023[45]). Quantitative Structure-Activity Relationship (QSAR) modeling is a computational method widely used to elucidate the relationships between chemical properties of the compounds and their biological activities (Nantasenamat et al., 2009[23]; Roy et al., 2015[35]). Among others, the multiple linear regression (MLR) algorithm is well-known for its interpretable nature that suitably reveals informative knowledge for guiding the effective design of new derivatives. Successful QSAR-driven rational drug designs have been reported for diverse activities by our group (Phanus-umporn et al., 2020[26]; Pingaew et al., 2022[27]; Prachayasittikul et al., 2017[28]; Pratiwi et al., 2019[30]; Worachartcheewan et al., 2020[48]). Drug-likeness is one of the key properties determining successful drug development, therefore, *in silico* drug-likeness prediction is widely included in the initial phases of development to improve the success rate (Ekins et al., 2023[7]). 

**Figure 1:**
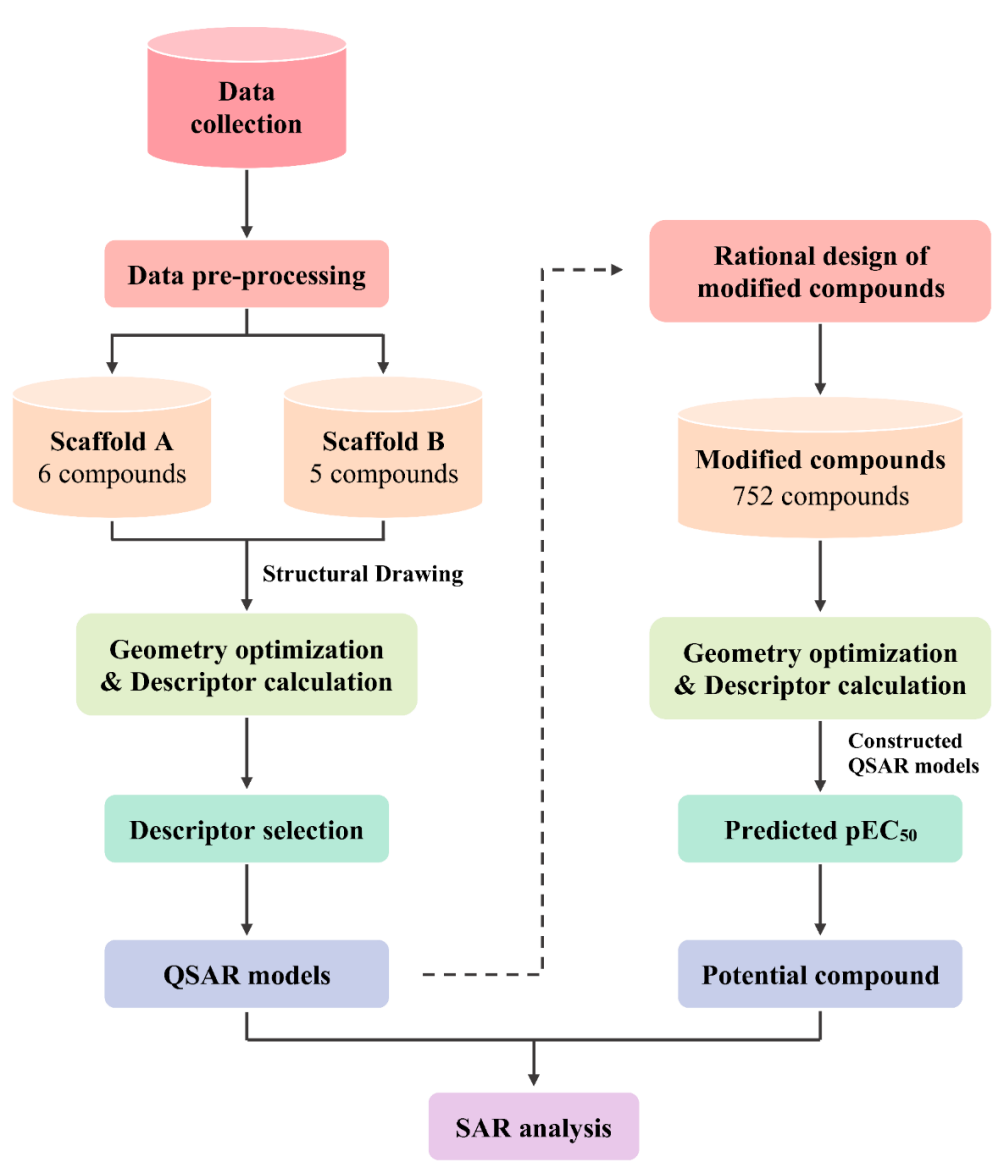
Overview workflow of the study

To date, QSAR studies regarding the S1PR modulating compounds are still limited. Herein, previously reported quinoxaline-based and indole-based S1PR2 activators (Pubchem, 2004[31][32][33]) (Figure 1(Fig. 1)) were retrieved as input datasets for QSAR modeling using MLR algorithms. The constructed models were further applied to guide the design of new structurally modified compounds. Subsequently, the newly designed compounds were predicted for their S1PR2 activating effects as well as drug-likeness. SAR analysis was also performed to reveal key beneficial knowledge. Finally, a set of promising novel compounds was highlighted for further potential development.

## Materials and Methods

### Data collection

**Figure 2:**
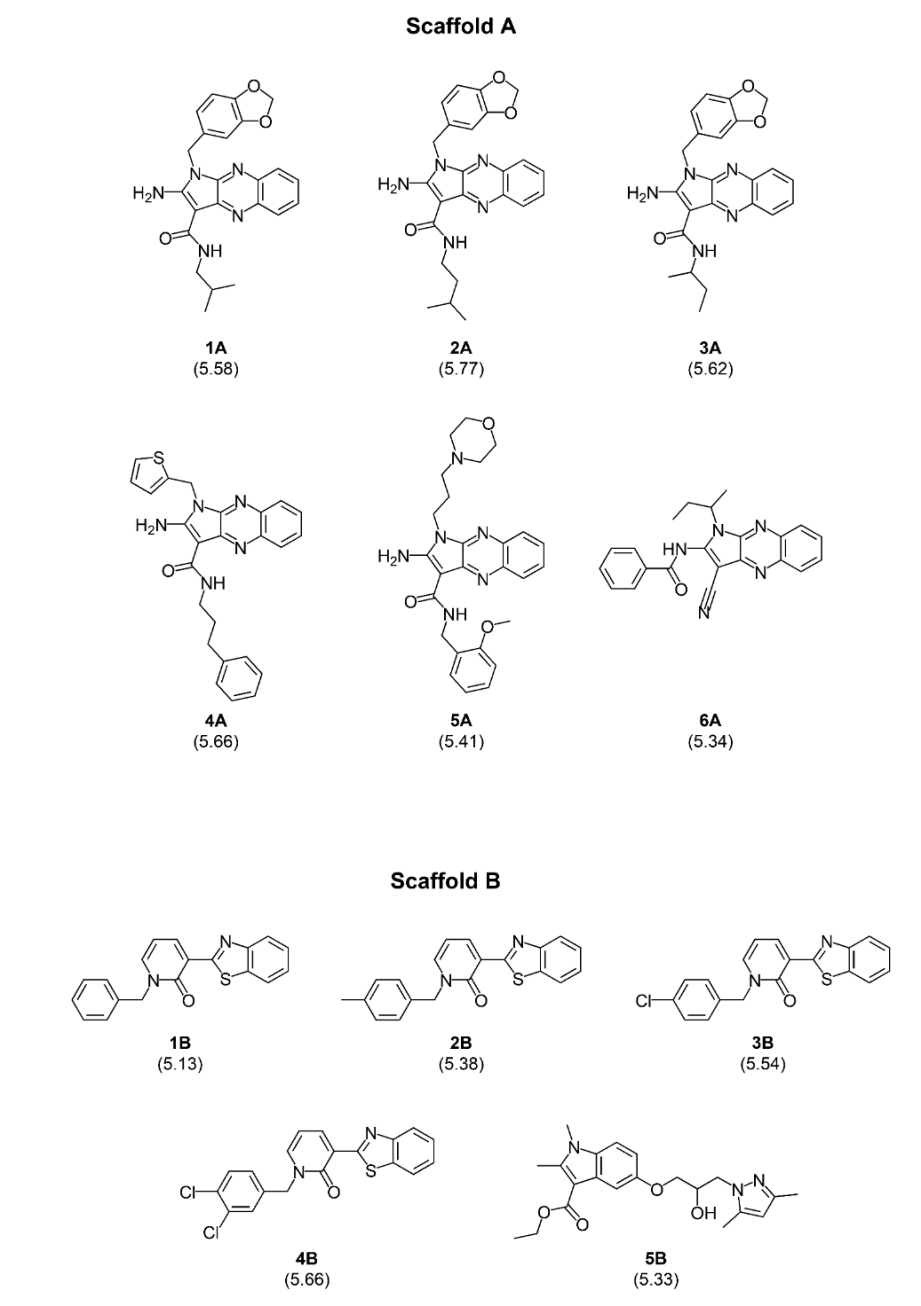
Chemical structures of quinoxaline-based (Scaffold A: 6 compounds) and indole-based (Scaffold B: 5 compounds) activators. Experimental pEC_50_ values of the compounds are presented in parentheses.

An original set of quinoxaline- and indole-based S1PR2 activators was obtained from the ChEMBL database (Mendez et al., 2018[22]) and was curated (Fourches et al., 2010[9]) by the following steps (i) removal of inorganic compounds (i.e., salts and mixtures), (ii) structural validation and cleaning, (iii) normalization of specific chemotypes, (iv) deletion of duplicates, and (v) final checking to obtain the final curated data set containing 11 active compounds along with their bioactivity values, represented by the half maximal effective concentration (EC_50_) (Neubig et al., 2003[24]). EC_50_ is a concentration that increases the S1P activity by 50 %. The EC_50_ values were converted into pEC_50_ values by taking the negative logarithm based 10 to normalize the data points. According to the compound's core structure, the dataset was separately preprocessed into two final datasets (i.e., scaffold A = 6 quinoxaline-based compounds and scaffold B = 5 indole-based compounds), Figure 2(Fig. 2).

### Geometry optimization

Chemical structures of compounds in SMILES format were converted into MOL format using MarvinSketch (ChemAxon Ltd., 2018[5]). All structures were geometrically optimized to obtain the lowest energy conformers by density functional theory (DFT) computation with Becke's three-parameter Lee-Yang-Parr hybrid functional (B3LYP) and 6-31g(d) basis set using Gaussian 09 software (Frisch et al., 2009[11]). The optimized structures were subsequently used as input files for descriptor calculation.

### Molecular descriptors calculation

Molecular descriptors are numerical values used to represent characteristics of the compounds in terms of connectivity, constitution, and physicochemical properties (Nantasenamat et al., 2009[23]). A set of 13 quantum chemical descriptors was calculated using an in-house developed script (i.e., mean absolute atomic charge (Q_m_), molecular dipole moment (µ), electronegativity (χ), total energy (E_T_), electron affinity (EA), ionization potential (IP), electron ionization (EI), highest occupied molecular orbital energy (HOMO), lowest unoccupied molecular orbital energy (LUMO), energy difference of HOMO  and LUMO (HOMO-LUMO_gap_), absolute hardness (η), softness (S), and electrophilicity (ω). Additionally, the optimized structures were used as input files for calculation of molecular descriptor using Dragon 5.5 software (Mauri et al., 2006[20]) to obtain a set of 3,224 molecular descriptors, comprising 48 constitutional descriptors, 119 topological descriptors, 47 walk and path counts, 33 connectivity indices, 47 information indices, 96 2D autocorrelations, 107 edge adjacency indices, 64 Burden eigenvalues, 21 topological charge indices, 44 eigenvalue-based indices, 41 Randic molecular profiles, 74 geometrical descriptors, 150 RDF descriptors, 160 3D-MoRSE descriptors, 99 WHIM descriptors, 197 GETAWAY descriptors, 154 functional group counts, 120 atom-centered fragments, 14 charge descriptors, 29 molecular properties, 780 2D binary fingerprints, and 780 2D frequency fingerprints.

### Descriptor selection

Descriptor selection is a process to select a set of informative descriptors from a large set of calculated descriptors to be used as final predictors. Correlation-based feature selection was initially performed to filter a set of descriptor variables that are highly correlated with bioactivity of the compounds. Pearson's correlation coefficient (r) values for each pair of descriptor value and bioactivity value (pEC_50_) were calculated. Descriptors with |*r*| ≥ 0.8 were considered as highly correlated predictors and were selected for further selection process using stepwise MLR in PASW Statistics 18 software (SPSS Inc, 2009[40]) and M5 method in WEKA software (Frank et al., 2016[10]) to obtain a final set of informative descriptors. Finally, the values of selected descriptors along with the pEC_50 _values were used to prepare final datasets for model construction.

### Model construction

Two final datasets were used as input files for construction of QSAR models by WEKA software 3.8.4 (Frank et al., 2016[10]) using MLR algorithm. The MLR model represents a linear relationship between multiple descriptors (X variables) and bioactivity (Y variable) as shown in equation (1).

*Y* = *B*_0_ + Σ*B_n_X_n_* (1)

where *Y* is the pEC_50_ of compounds, *B*_0_ is the Y-intercept and *B*_n_ are the regression coefficients of descriptors (*X*_n_).

### Data sampling and model validation

The dataset was divided into training set and testing set by leave-one-out cross validation (LOO-CV). Since the datasets contain small samples with less than 50 compounds, the LOO-CV sampling method is reliable for model validation (Roy et al., 2015[36]). The training set is used to build the model whereas the testing set was used to validate the predictive performance of the built model. For each round, one sample was excluded from the whole dataset (N) to be used as a testing set (in which its activity was predicted using the trained model) whereas the remaining samples (N-1) were used as a training set to train and construct the model. The same process was repeated until every compound in the dataset was selected to be used as a testing set (Sammut and Webb, 2011[37]).

### Assessment of model performance

The constructed models were assessed for their predictive performance using two statistical parameters i.e., correlation coefficient (R) and root mean square error (RMSE). The first parameter reflects predictive correlation of the model whereas the second one represents predictive error (Rácz et al., 2015[34]).

### Application of the constructed models for guiding the design of modified compounds

To increase structural diversity, a set of 752 structurally modified compounds was rationally designed based on key descriptors presented in the constructed QSAR models. All newly designed compounds were undergone the same processes as their parent compounds (e.g., drawing, geometrical optimization, and descriptor calculation) to obtain their informative descriptor values, and their S1P activities (pEC_50 _values) were subsequently predicted using the constructed models.

### In silico drug-likeness prediction

The newly designed compounds were predicted for their drug-likeness using *in silico* web-based tool SwissADME (Daina et al., 2017[6]). Chemical structures in SMILES format were uploaded for prediction. The drug-likeness of the compounds was assessed based on Lipinski's rule of five (LRo5).

## Results and Discussion

### QSAR modeling

Two final datasets of 6 quinoxaline-based (scaffold A) and 5 indole-based (scaffold B) S1PR2 activators were prepared for construction of two QSAR models, Figure 2(Fig. 2). Compounds were preprocessed to obtain their descriptor values. Bioactivity values were normalized into pEC_50 _values. Descriptor selection was performed to select a final set of 6 informative descriptors for construction of scaffold A model (4 descriptors: R2v+, MATS5m, nR05, and Km) and scaffold B model (2 descriptors: H3e and E3v), Table 1(Tab. 1). Dataset and predicted activities of scaffolds A and scaffold B models are provided in Tables 2(Tab. 2) and 3(Tab. 3).

**Figure 3:**
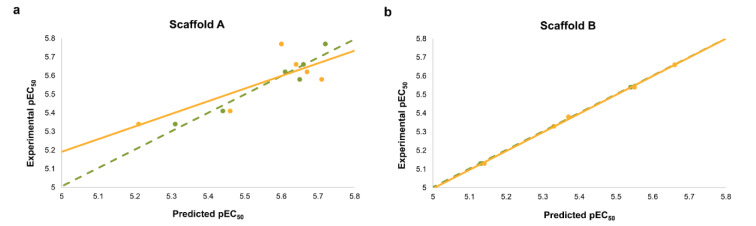
Comparative plots of experimental pEC_50_ and predicted pEC_50_ values from two QSAR models. a: Scaffold A (quinoxaline-based compounds), b: Scaffold B (indole-based compounds). Plots of training set are presented as green circles and dotted regression lines.

Two QSAR models were successfully constructed using MLR (i.e., scaffold A and scaffold B models). Two built models provided acceptable predictive performance as shown by their validated statistical values for both training and testing sets (R_tr_ = 0.9667-0.9997, RMSE_tr_ = 0.0046-0.0375, Q_cv_ = 0.7902-0.9989, and RMSE_cv_ = 0.0093-0.1057, Table 4(Tab. 4)). Comparative plots of experimental pEC_50_ versus predicted pEC_50_ values are provided in Figure 3(Fig. 3).

From scaffold A, four descriptors play roles as predictors for the activity of quinoxaline-based activators. According to regression coefficient, the most influential descriptor was noted to be a van der Waals volume descriptor (R2v+) followed mass descriptors (MATS5m and Km), and number of five-membered ring (nR05), respectively (Table 4(Tab. 4)). The high positive values of R2v+ and MATS5m together with low positive or high negative values of the nR05 and Km are required to provide potent activity (high pEC_50_ value). For scaffold B, the van der Waals volume descriptor (E3v) also played major influence on S1PR activating effect, followed by the electronegativity (H3e) descriptor (Table 4(Tab. 4)). Herein, both predictors possessed positive regression coefficient (R) values suggesting their positive effects on the pEC_50_ values.

### Application of the QSAR models for rational design of modified compound series

Key descriptors of the constructed models were further used to guide the design of new derivatives. Modification strategies are conceptually depicted in supplementary information section 1.2 and 2.2 (Tables S2 and S5). Finally, two sets of structurally modified compounds were designed (i.e., scaffold A = 635 compounds, scaffold B = 117 compounds). Chemical structures of the newly designed compounds and their predicted pEC_50_ values are provided in supplementary data and supplementary information, Figures S1-S11. In overview, most of the modified compounds exhibit improved predicted activity when compared to their parents.

### Structure-Activity Relationship (SAR) analysis

**Figure 4:**
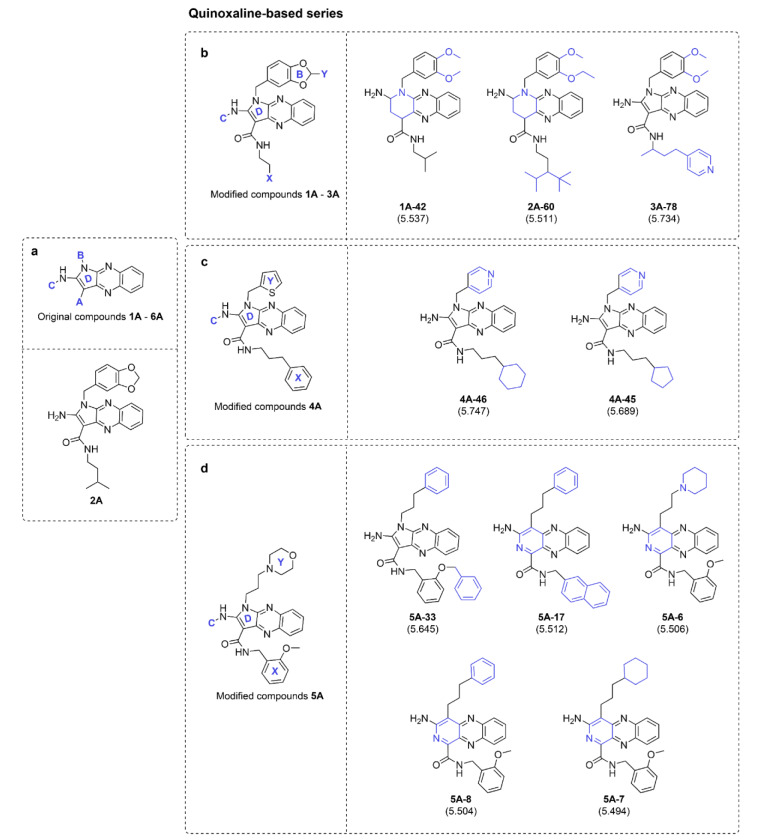
Summary of the most potent quinoxaline-based original compound (a) and modified series 1A-5A (b-d). Predicted pEC_50_ values of the compounds are presented in parentheses.

**Figure 5:**
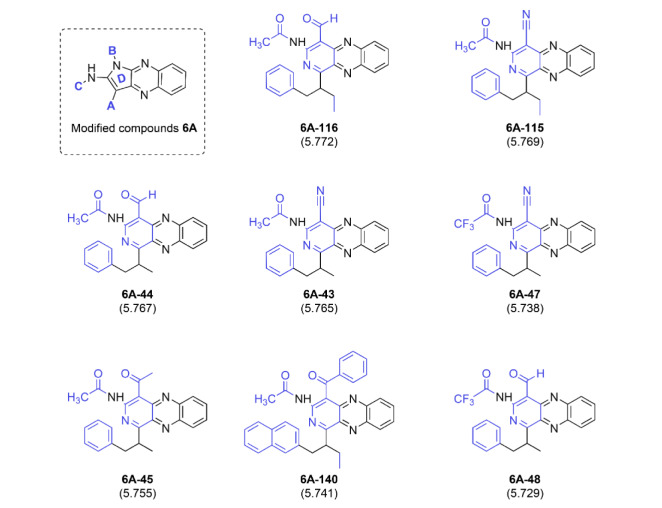
Summary of the most potent compounds from modified series 6A. Predicted pEC_50_ values of the compounds are presented in parentheses.

**Figure 6:**
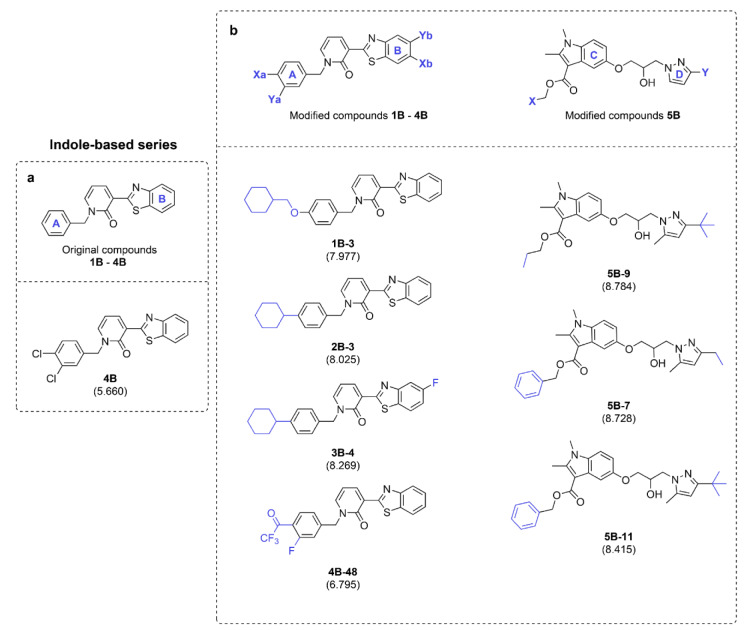
Summary of the most potent indole-based original compounds (a), and modified series (b). Predicted pEC_50_ values of the compounds are presented in parentheses.

Understanding structure-activity relationship (SAR) is essential for successful drug development (Macalino et al., 2015[18]; Roy et al., 2015[35]). Herein, SAR analysis was performed to reveal key features influencing S1PR activating effect of both compound series. For each series, a modification template is illustrated to facilitate effective discussion (as shown in the main panels of Figures 4(Fig. 4) and 6). Insight analysis of each sub-modified series was provided to suggest a potential strategy for rational design and development of new derivatives with improved properties. Additionally, the most potent compounds of each subseries are summarized in Figures 4(Fig. 4), 5(Fig. 5), and 6(Fig. 6).

From the original quinoxaline-based activators, compound **2A** (pEC_50 _= 5.77) is the most potent compound followed by **4A**,** 3A**,** 1A**,** 5A**, and **6A** (pEC_50 _= 5.66, 5.62, 5.58, 5.41, and 5.34, respectively, Table 2(Tab. 2). An increased length of the alkyl chain amide linker at A site on ring D (Figure 4a(Fig. 4)) could improve activity via increasing MATS5m values (**4A **and** 3A**, Table 2(Tab. 2)). The presence of long-length alkyl chain at A site is required to give high R2V+ and MATS5m values, thereby, high pEC_50_ values of the compounds (as observed for compounds **2A** and **4A**). The aromatic ring (i.e., benzene, and thiophene) nearly attached at B site is also essential for potent activity. Two compounds without (**6A**) or with aromatic ring attached at this position in further distance (**5A**) provided lower nR05 value (nR05 =1) leading to their lesser potency when compared to other compounds (**1A-4A** with nR05 =2), Figure 4a(Fig. 4) and Table 2(Tab. 2). Summarized SAR analysis of the original compounds **1A**-**6A** is provided in supplementary information, Table S1.

An additional set of 635 modified quinoxaline-based activators were designed. The modified compounds were grouped by their main core structures into three main templates (Figure 4b-d(Fig. 4)) and their modification strategies are provided in supplementary information Table S2. Summarized SAR analysis of the modified compounds **1A**-**6A** is provided in supplementary information, Table S3. In overview, the modified series **6A** provided the most potent predicted activities (predicted pEC_50_ = 5.262-5.772, supplementary data, Scaffold A). Notably, the replacement of nitrogen-containing ring D from the five-membered (pyrrole) to six-membered ring (piperidine or pyridine) gave various effects depending on the parent compounds. Improved activities were observed for the six-membered ring modified series **5A-6A**, whereas decreased activities were found for those of the series **1A-4A**. This phenomenon could be due to the decreased nR05 values (supplementary data, Scaffold A).

For the best modified series **6A**, modifications were performed on A, B, and C sites to obtain 287 modified compounds. Most of them provided better activities (predicted pEC_50_ = 5.262-5.772, supplementary data, Scaffold A) than the parent **6A** (experimental pEC_50 _= 5.340, Table 2(Tab. 2)). Compounds **6A-116 **and **6A-115 **were the two most potent compounds (predicted pEC_50_: **6A-116** = 5.772 and **6A-115 **= 5.769). The improved activity (higher predicted pEC_50_) was suggested to be via decreasing the nR05 and Km values while increasing the R2v+ value (supplementary information, Table S3). A set of promising compounds in modified **6A** series is summarized in Figure 5(Fig. 5). Some essential key modifications to achieve preferable activity of series **6A** were revealed such as 1) a replacement of ring D with six-membered ring, 2) modification on A site by an insertion of a moiety containing terminal benzene ring, 3) a modification on B site by replacing the branched alkyl chain with -COH and -CN groups as well as 4) a modification on C site by substitutions of -COCH_3_ or -COCF_3_ gave compounds with better activities than those substituted with benzoyl group. The better activities were provided when the longer chain was substituted at A position (**6A-116 **>** 6A-44** and **6A-115 **>** 6A-43**, Figure 5(Fig. 5)). Substitution at B position with -COH group provided better activity than with the CN group (**6A-116 **>** 6A-115** and **6A-44 **>** 6A-43**, Figure 5(Fig. 5)). The replacement of COH group on B position of compound **6A-44** (predicted pEC_50_ = 5.767) by the COCH_3_ group gave compound **6A-45** (predicted pEC_50_ = 5.755) with the lesser potency. Similarly, the bulky ring substitutions on A and B positions impaired activity of compound **6A-140 **(predicted pEC_50_ = 5.741) when compared to **6A-116** (predicted pEC_50_ = 5.772). Additionally, the substitution at C position with acetyl group (COCH_3_) gave the more potent compounds rather than the COCF_3_ group (**6A-43 **>** 6A-47** and **6A-44 **>** 6A-48**). The lesser potency of these COCF_3 _derivatives was suggested to be via a shift of MATS5m value to positive values and the increased Km value (supplementary data, Scaffold A).

Activity of the original indole-based compounds (scaffold B) was ranked as **4B **>** 3B **>** 2B **>** 5B **>** 1B** (pEC_50 _= 5.66, 5.54, 5.38, 5.33, and 5.13, respectively, Table 3(Tab. 3)). The two most potent compounds (**4B** and **3B**) are chloro-containing derivatives indicating that substitutions on ring A with chlorine (Cl) atom provides better improved activity than substitutions with alkyl (**2B**) or hydrogen (**1B**) groups. This could be via the increasing values of both H3e and E3v descriptors (Figure 2(Fig. 2) and Table 3(Tab. 3)). Moreover, an insertion of additional chloro group on the A ring of **3B** gave the **4B** compound with increased activity via increasing the value of van der Waals volume (**4B **>** 3B**: H3e value: **4B** = 2.832, **3B** = 2.74, Table 3(Tab. 3)). Summary SAR analysis of original compounds **1B**-**5B** is provided in supplementary information, Table S4.

The QSAR scaffold B model was used to guide a design of 117 modified compounds (supplementary information, Table S4) and are subdivided into 5 subseries (**1B**-**5B**). The most potent compounds of each subseries are summarized in Figure 6b(Fig. 6). In overview, the modifications provided compounds with improved activities in every subseries. The substitution on Xa position of ring A with the ring-containing moiety (aliphatic ring: **1B**-**3**,** 2B**-**3**, and **3B**-**4** or aromatic ring: **4B-48**) provided the best activities when compared to other types of moieties (i.e., -OH, -OCH_3_, -OCF_3_, and alkyl chain). Notably, improvement was observed when the -CH_3_ group of the parent **2B** (experimental pEC_50_ = 5.38) is replaced by a cyclohexane ring to give compound **2B-3** (predicted pEC_50_ = 8.025). The same improvement was noticed when comparing parent mono-chloro containing compound **3B** (experimental pEC_50_ = 5.54) with the modified **3B-4** (predicted pEC_50_ = 8.269). The improved activity of the **3B-4** is also influenced by a substituted fluorine (F) atom on the Yb moiety of ring B. The type of halogen atom substituted on this Yb position also affected the activity of the compounds as observed for modified subseries **3B**. The best activity is achieved by substitution with fluorine (F: **3B-4**: pEC_50_ = 8.269) followed by chlorine (Cl:** 3B-5**: pEC_50_ = 8.223) > bromine (Br: **3B-6**: pEC_50_ = 8.148) > iodine (I:**3B-7**: pEC_50_ = 8.061), supplementary information, Table S5 and supplementary data, Scaffold B). For modified **4B** series, the compounds with COCF_3 _substitution on Xa position (**4B-48** to **4B-55**: predicted pEC_50 _= 6.275-6.795, supplementary information, Figure S10) provided the best improvement among others. This could be due to their high E3v value (all compounds possess E3v value > 3, greater than those of other series).

For compound **5B** possessing different core structure, the modifications effectively improved activity (as shown by high predicted pEC_50 _= 5.794-8.784, supplementary information, Table S5 and supplementary data, Scaffold B). The best improvements were noted when the X moieties are substituted by alkyl chain (**5B-9**) or aromatic ring (**5B-7** and **5B-11**) moieties, while the Y moieties are replaced with long (**5B-7**) or branched alkyl (**5B-9** and **5B-11**) chain, Figure 6b(Fig. 6). 

### Predicted drug-likeness of the newly designed compounds

**Figure 7:**
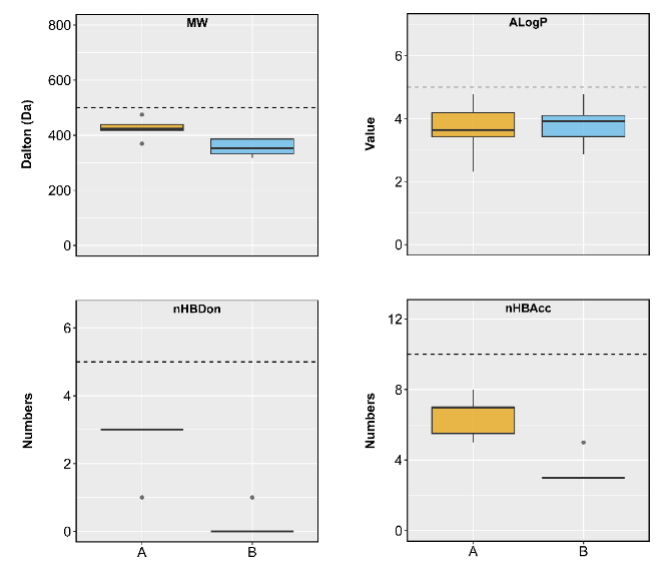
The distribution of Lipinski's descriptors of scaffold A (yellow) and scaffold B (blue). Abbreviation: MW, molecular weight; AlogP, Ghose-Crippen-Viswanadhan octanol-water partition coefficient; nHBDon, number of hydrogen bond donors; nHBAcc, number of hydrogen bond acceptors

Regarding the Lipinski's rule, newly designed compounds were predicted for their drug-like parameters including molecular weight (MW), Ghose-Crippen-Viswanadhan octanol-water partition coefficient (AlogP), number of hydrogen bond donors and acceptors (nHBDon and nHBAcc) (Lipinski et al., 2001[17]). The parameter values were visualized as distribution plots (Figure 7(Fig. 7)). It was shown that all compounds are accepted by the rule of five, suggesting that they are drug-like molecules with the possibility for further development.

## Conclusion

This study demonstrates the utilization of QSAR modeling for facilitating the effective design of novel nitrogen-based heterocyclic S1PR2 activators. Two QSAR models were successfully constructed and provided good predictive performance (R_tr_ = 0.9667-0.9997, RMSE_tr_ = 0.0046-0.0375, Q_cv_ = 0.7902-0.9989, and RMSE_cv_ = 0.0093-0.1057). The constructed models were further applied to guide the design and activity prediction of an additional set of 752 modified compounds. The QSAR-driven modification on scaffold B outperformed those on scaffold A in improving the activities (predicted pEC_50_ values: modified scaffold B > 6.00, modified scaffold A ≤ 5.80). All newly designed compounds were predicted to be drug-like molecules. Additionally, the models revealed key properties required for potent S1PR2 activities including van der Waals volume (R2v+ and E3v), mass (MATS5m and Km), number of 5-membered rings (nR05), and electronegativity (H3e), which would be beneficial for future design, screening, and development of the related compounds. Finally, a set of 25 newly designed compounds with outstanding predicted activities (scaffold A = 18 and scaffold B = 7 compounds) were highlighted for further development. This work demonstrates an initial step in the discovery journey of the novel osteoanabolic agents, and further studies regarding the synthesis and biological investigations (i.e., *in vitro*, *in vivo*, clinical trials) are essentially recommended.

## Notes

Veda Prachayasittikul and Aungkura Supokawej (Department of Clinical Microscopy, Faculty of Medical Technology, Mahidol University, Bangkok 10700, Thailand; Phone: +66(0)2 441 4371-5 Ext. 2723, Fax: +66(0)2 441 4380, E-mail: aungkura.jer@mahidol.ac.th) contributed equally as corresponding author.

## Declaration

### Supplementary information

Supplementary information and supplementary data are available on EXCLI Journal's website.

### Conflict of interest

There are no conflicts to declare.

### Acknowledgments

Rattanawan Tangporncharoen is supported by the Royal Golden Jubilee (RGJ) Ph.D. Programme (grant no. PHD/0066/2561), through the National Research Council of Thailand (NRCT), Thailand Research Fund (TRF) and Thailand Science Research and Innovation (TSRI). Veda Prachayasittikul was supported by Office of the Permanent Secretary, Ministry of Higher Education, Science, Research and Innovation, Research Grant for New Scholar (grant no. RGNS 64-167), and by Mahidol University (Fundamental Funds: fiscal year 2023 by National Science Research and Innovation Fund (NSRF)). Chuleeporn Phanus-Umporn was supported by Office of the Permanent Secretary, Ministry of Higher Education, Science, Research and Innovation (grant no. RGNS 65-145).

## Supplementary Material

Supplementary information

Supplementary data

## Figures and Tables

**Table 1:**
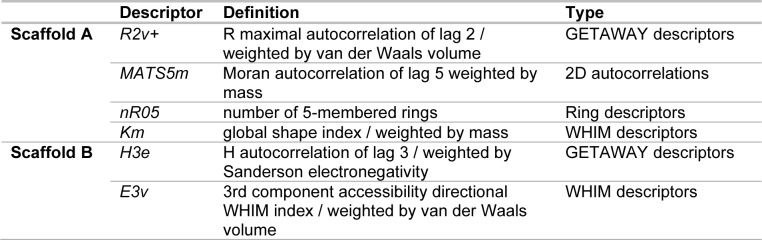
Definitions of informative descriptors used for QSAR modeling

**Table 2:**
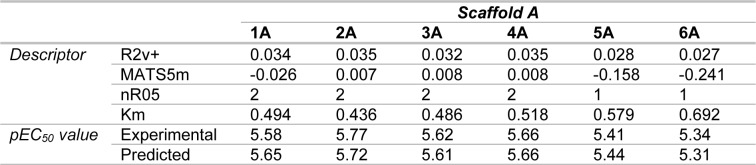
Dataset and predicted activities of scaffold A compounds (1A - 6A)

**Table 3:**
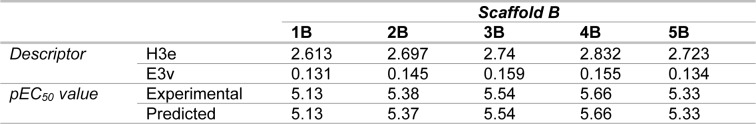
Dataset and predicted activities of scaffold B compounds (1B - 5B)

**Table 4:**
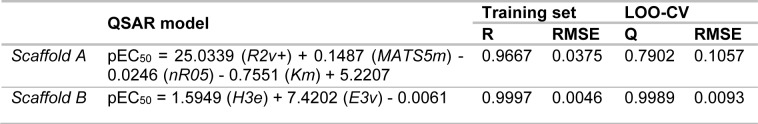
Summary of predictive performance of the constructed QSAR models
