# Supplementary material for: Computer-guided design of novel nitrogen-based heterocyclic sphingosine-1-phosphate (S1P) activators as osteoanabolic agents
Source: EXCLI J. 2024 May 27;23:818–32. doi: 10.17179/excli2024-7214 (PMC11579520; doi:10.17179/excli2024-7214)
Supplement: Supplementary information [file EXCLI-23-818-s-001.pdf]

EXCLI Journal 2024;23:818-832 – ISSN 1611-2156
Received: March 26, 2024, accepted: April 17, 2024, published: May 27, 2024

Supplementary information to:

Original article:

COMPUTER-GUIDED DESIGN OF NOVEL NITROGEN-BASED HETEROCYCLIC SPHINGOSINE-1-PHOSPHATE (S1P) ACTIVATORS AS OSTEOANABOLIC AGENTS

Rattanawan Tangporncharoen[1], Chuleeporn Phanus-Umporn[2], Supaluk Prachayasittikul[3], Chanin Nantasenamat[4], Veda Prachayasittikul[3*], Aungkura Supokawej[1*]

[1] Department of Clinical Microscopy, Faculty of Medical Technology, Mahidol University, Bangkok 10700, Thailand
[2] Department of Community Medical Technology, Faculty of Medical Technology, Mahidol University, Bangkok 10700, Thailand
[3] Center for Research Innovation and Biomedical Informatics, Faculty of Medical Technology, Mahidol University, Bangkok 10700, Thailand
[4] Streamlit Inc., San Francisco, CA 94121, USA

* **Corresponding authors:** Aungkura Supokawej, Department of Clinical Microscopy, Faculty of Medical Technology, Mahidol University, Bangkok 10700, Thailand. Phone: +66(0)2 441 4371-5 Ext. 2723; Fax: +66(0)2 441 4380; E-mail: aungkura.jer@mahidol.ac.th
Veda Prachayasittikul, Center for Research Innovation and Biomedical Informatics, Faculty of Medical Technology, Mahidol University, Bangkok 10700, Thailand. Phone: +66(0)2 441 4371-5 Ext. 2726; Fax: +66(0)2 441 4380; E-mail: veda.pra@mahidol.ac.th

https://dx.doi.org/10.17179/excli2024-7214

This is an Open Access article distributed under the terms of the Creative Commons Attribution License (https://creativecommons.org/licenses/by/4.0/).

Received: March 26, 2024, accepted: April 17, 2024, published: May 27, 2024

SUPPLEMENTARY FILES

Supplementary data

An excel file containing chemical structures in SMILES format, descriptor values, and predicted activities of modified compounds series A (Scaffold A) and B (Scaffold B).

Supplementary information contents Page

Table S1 Summary SAR analysis of scaffold A original compounds (**1A-6A**) S3

Table S2 Modification strategies for rational design of modified compounds in scaffold A S4

Table S3 Summary SAR analysis of modified compounds in scaffold A S9

Table S4 Summary SAR analysis of scaffold B original compounds (**1B-5B**) S13

Table S5 Summary SAR analysis of modified compounds in scaffold B S14

Figure S1 Chemical structure of modified compounds **1A** S17

Figure S2 Chemical structure of modified compounds **2A** S21

Figure S3 Chemical structure of modified compounds **3A** S25

Figure S4 Chemical structure of modified compounds **4A** S30

Figure S5 Chemical structure of modified compounds **5A** S33

Figure S6 Chemical structure of modified compounds **6A** S36

Figure S7 Chemical structure of modified compounds **1B** S47

Figure S8 Chemical structure of modified compounds **2B** S48

Figure S9 Chemical structure of modified compounds **3B** S48

Figure S10 Chemical structure of modified compounds **4B** S49

Figure S11 Chemical structure of modified compounds **5B** S51

EXCLI Journal 2024;23:818-832 – ISSN 1611-2156
Received: March 26, 2024, accepted: April 17, 2024, published: May 27, 2024

Section 1: Summary SAR analysis scaffold A

Section 1.1: Original set (1A-6A)

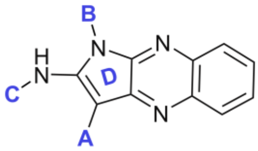

Original compounds **1A** - **6A**

Table S1 Summary SAR analysis of scaffold A original compounds **(1A-6A)**

Compound	A site	B site	Summary SAR
**1A – 3A**	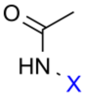 X = alkyl chain linker with terminal branched alkyl chain	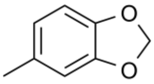	**Activity: 2A** > **4A** > **3A** > **1A** > **5A** > **6A**<br>• Compounds with A site containing longer alkyl chain linker provided better activity than short linker (**2A** > **3A** > **1A**). The longer chain linker provided the higher MATS5m value.<br>• The number of five-membered rings presented in the molecule at B site is important to potent activity of the compounds via affecting the nR05 values. Compounds **5A** and **6A** with absence of substituted five-membered ring at B site provide the lower nR05 value (nR05 =1) and lesser potency than those of five-membered ring substituted derivatives **1A-4A** (with nR05 =2).<br>• The presence of long-length alkyl chain at A site is required to give high R2V+ and MATS5m values and high pEC_{50} values of the compounds in this class (as observed for compounds **2A** and **4A**).
**4A**	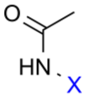 X = alkyl chain linker terminal substituted benzene ring	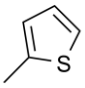	
**5A**	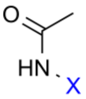 X = alkyl chain linker terminal substituted benzene ring	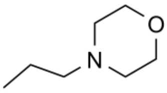	
**6A**	—C≡N<br>Cyanide	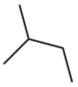 Alkyl chain	

EXCLI Journal 2024;23:818-832 – ISSN 1611-2156
Received: March 26, 2024, accepted: April 17, 2024, published: May 27, 2024

Table S1 (cont.) Summary SAR analysis of scaffold A original compounds **(1A-6A)**

Overview key summary

- Activity: **2A** > **4A** > **3A** > **1A** > **5A** > **6A**
- Compounds with A site containing longer alkyl chain linker provided better activity than short linker (**2A** > **3A** > **1A**). The longer chain linker provided the higher MATS5m value.
- The number of five-membered rings presented in the molecule at B site is important to potent activity of the compounds via affecting the nR05 values. Compounds **5A** and **6A** with absence of substituted five-membered ring at B site provide the lower nR05 value (nR05 =1) and lesser potency than those of five-membered ring substituted derivatives **1A-4A** (with nR05 =2).
- The presence of long-length alkyl chain at A site is required to give high R2V+ and MATS5m values and high pEC_{50} values of the compounds in this class (as observed for compounds **2A** and **4A**).

Section 1.2: Modified compounds *(635 compounds)*

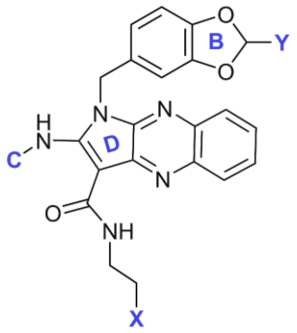

Modified compounds **1A** - **3A**

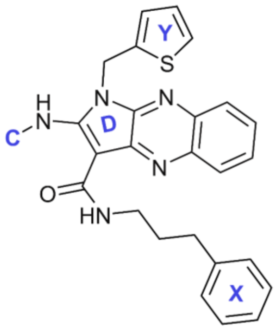

Modified compounds **4A**

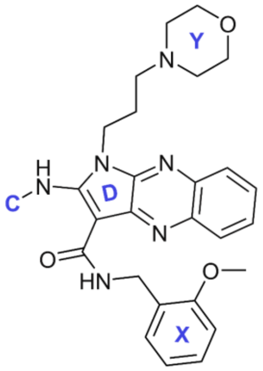

Modified compounds **5A**

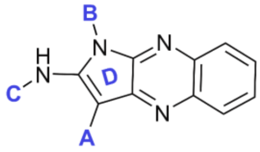

Modified compounds **6A**

Table S2 Modification strategies for rational design of modified compounds in scaffold A

Modified Series	Subseries	Ring D modification	A site / X site modification	B site / Y site modification	C site modification
**1A**<br>(81 compounds)	**1A-1**<br>**1A-11**<br>**1A-21**<br>**1A-31**	No	X modification:<br>↑ length of alkyl chain<br>↑ branching of alkyl chain	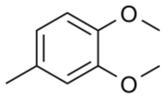<br>Ring B:<br>Open five-membered ring<br>Y site:<br>↑ length of substituted alkyl chain	No

EXCLI Journal 2024;23:818-832 – ISSN 1611-2156
Received: March 26, 2024, accepted: April 17, 2024, published: May 27, 2024

Table S2 (cont.) Modification strategies for rational design of modified compounds in scaffold A

Modified Series	Subseries	Ring D modification	A site / X site modification	B site / Y site modification	C site modification
	**1A-42**<br>**1A-52**<br>**1A-62**<br>**1A-72**	Yes<br>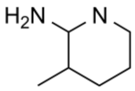	X modification:<br>↑ length of alkyl chain<br>↑ branching of alkyl chain	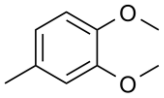<br>Ring B:<br>Open five-membered ring<br>Y site:<br>↑ length of substituted alkyl chain	No
**2A**<br>(83 compounds)	**2A-1**<br>**2A-11**<br>**2A-21**<br>**2A-31**	No	X modification:<br>↑ length of alkyl chain<br>↑ branching of alkyl chain	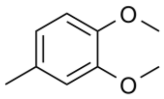<br>Ring B:<br>Open five-membered ring<br>Y site:<br>↑ length of substituted alkyl chain	No
	**2A-42**<br>**2A-52**<br>**2A-62**<br>**2A-72**	Yes<br>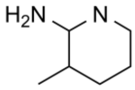	X modification:<br>↑ length of alkyl chain<br>↑ branching of alkyl chain	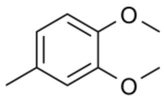<br>Ring B:<br>Open five-membered ring<br>Y site:<br>↑ length of substituted alkyl chain	No
**3A**<br>(89 compounds)	**3A-1**	Yes<br>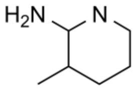	X modification:<br>↑ length of alkyl chain<br>↑ branching of alkyl chain	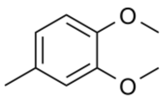<br>Ring B:<br>Open five-membered ring<br>Y site:<br>↑ length of substituted alkyl chain	No

EXCLI Journal 2024;23:818-832 – ISSN 1611-2156
Received: March 26, 2024, accepted: April 17, 2024, published: May 27, 2024

Table S2 (cont.) Modification strategies for rational design of modified compounds in scaffold A

Modified Series	Subseries	Ring D modification	A site / X site modification	B site / Y site modification	C site modification
	**3A-2**<br>**3A-3**<br>**3A-4**<br>**3A-8**	Yes<br>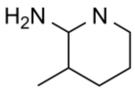	X modification:<br>Insert different five- and six-membered rings at terminal of the chain	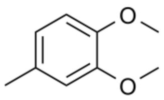<br>Ring B:<br>Open five-membered ring<br>Y site:<br>↑ length of substituted alkyl chain<br>Ring substitution at terminal chain of B position	No
	**3A-46**<br>**3A-50**<br>**3A-51**<br>**3A-52**<br>**3A-56**	No	No	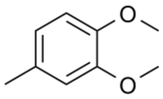<br>Ring B:<br>Open five-membered ring<br>Y site:<br>↑ length of substituted alkyl chain<br>Ring substitution at terminal chain of B position	No
**4A**<br>(56 compounds)	**4A-1**<br>**4A-4**	Yes<br>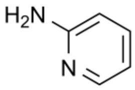	Change type of X ring	Change type of Y ring	No
	**4A-29**<br>**4A-32**	No	Change type of X ring	Change type of Y ring	No
**5A**<br>(39 compounds)	**5A-1**	Yes<br>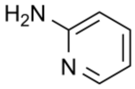	No	Change type of Y ring	No

EXCLI Journal 2024;23:818-832 – ISSN 1611-2156
Received: March 26, 2024, accepted: April 17, 2024, published: May 27, 2024

Table S2 (cont.) Modification strategies for rational design of modified compounds in scaffold A

Modified Series	Subseries	Ring D modification	A site / X site modification	B site / Y site modification	C site modification
	**5A-2**<br>**5A-3**<br>**5A-4**	Yes<br>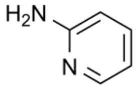	Change type of X ring<br>Change substituted group of X ring	Change type of Y ring	No
	**5A-21**<br>**5A-22**<br>**5A-23**	No	No	Change type of Y ring	No
	**5A-24**<br>**5A-25**<br>**5A-26**<br>**5A-27**	No	Change type of X ring<br>Change substituted group of X ring	Change type of Y ring	No
**6A**<br>(287 compounds)	**6A-1**<br>**6A-2**<br>**6A-3**<br>**6A-4**	Yes<br>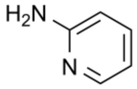	Change type of A substitution	No	No
	**6A-5**	Yes<br>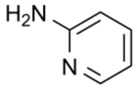	No	No	Change type of C substitution
	**6A-7**<br>**6A-8**<br>**6A-9**<br>**6A-10**	Yes<br>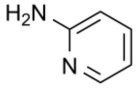	No	Substitution at terminal chain of B position with several types of rings	Change type of C substitution
	**6A-73**	Yes<br>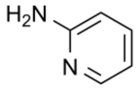	Change type of A substitution	Y site:<br>↑ length of substituted alkyl chain	No
	**6A-74**<br>**6A-75**<br>**6A-76**	Yes<br>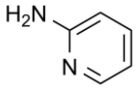	Change type of A substitution	Y site:<br>↑ length of substituted alkyl chain	No
	**6A-77**	Yes<br>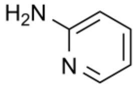	Change type of A substitution	Y site:<br>↑ length of substituted alkyl chain	Change type of C substitution

EXCLI Journal 2024;23:818-832 – ISSN 1611-2156
Received: March 26, 2024, accepted: April 17, 2024, published: May 27, 2024

Table S2 (cont.) Modification strategies for rational design of modified compounds in scaffold A

Modified Series	Subseries	Ring D modification	A site / X site modification	B site / Y site modification	C site modification
	**6A-79**<br>**6A-80**<br>**6A-81**<br>**6A-82**	Yes<br>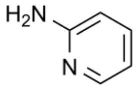	Change type of A substitution	Y site:<br>↑ length of substituted alkyl chain<br>Ring substitution at terminal chain of B position	Change type of C substitution
	**6A-145**<br>**6A-146**<br>**6A-147**	No	Change type of A substitution	No	No
	**6A-148**	No	Change type of A substitution	No	Change type of C substitution
	**6A-156**	No	Change type of A substitution	Ring substitution at terminal chain of B position	Change type of C substitution
	**6A-168**<br>**6A-180**<br>**6A-192**	No	Change type of A substitution	Ring substitution at terminal chain of B position	Change type of C substitution
	**6A-216**<br>**6A-217**<br>**6A-218**<br>**6A-219**	No	Change type of A substitution	Y site:<br>↑ length of substituted alkyl chain	No
	**6A-220**	No	Change type of A substitution	Y site:<br>↑ length of substituted alkyl chain	Change type of C substitution
	**6A-228**<br>**6A-240**<br>**6A-252**<br>**6A-264**	No	Change type of A substitution	Y site:<br>↑ length of substituted alkyl chain<br>Ring substitution at terminal chain of B position	Change type of C substitution

EXCLI Journal 2024;23:818-832 – ISSN 1611-2156
Received: March 26, 2024, accepted: April 17, 2024, published: May 27, 2024

Table S3 Summary SAR analysis of modified compounds in scaffold A

Modified Series	Effect of structure modification (Original VS Modified compounds)	Most potent compound	Type / position of substitution	Influence of descriptor variables
**1A**<br>(81 compounds)	All modified compounds = decreased activity<br>(**1A-42** > **1A-47** >**1A-2** > **1A-5**)	**1A-42**	Change ring D<br>Open ring B<br>Y site: substitutions with di-OCH_3	↓ nR05<br>↑ MATS5m<br>(positive value)
			Open ring B decreases activity of the compounds (**1A** > all modified 1A)<br>Open ring B together with increasing length of alkyl chain (X position) leads to decreased activity of the compounds (**1A-1** > **1A-11** > **1A-21** > **1A-31**)<br>Changing type of ring D to six-membered ring decreased activity of the compounds by mainly decreasing the value of nR05 to 0.	
**2A**<br>(83 compounds)	All modified compounds = decreased activity<br>(**2A-60** > **2A-72** > **2A-1** > **2A-82**)	**2A-60**	Change ring D<br>Open ring B<br>Y site: substitutions with branched multiple -OCH_3 group	↓ nR05<br>↑ MATS5m<br>(positive value)
			Same as observed from modifications of 1B series	
**3A**<br>(89 compounds)	All modified compounds = increased activity<br>(**3A-78** > **3A-80** > **3A-79** > **3A-81**)<br><br>Subseries provided best activity = **subseries 3A-78**	**3A-78**	Open ring B<br>Y site: ↑ length of substituted alkyl chain<br>Ring substitution at terminal chain of B position	↓ nR05<br>↓ Km
			Changing type of ring D to six-membered ring decreased activity of the compounds.<br>Open ring B and attachment of longer alkyl chain on Y position gave the compounds with decreased activity.<br>Subseries provided best activity = **subseries 3A-78** (original ring D, opened ring B with -OCH_3 substituted on Y positions, position X is modified by alkyl chain linker with terminal pyridine ring, and no modification on position C).<br>Modification of at X position by inserting terminal ring types gave compounds with various activities.<br>• When D ring is six-membered ring and ring B is opened<br>○ Substitutions of X containing pyridine (**3A-5** – **3A-7**) and naphthalene (**3A-38**) at terminal of the chain gave compounds with improved activity.	

EXCLI Journal 2024;23:818-832 – ISSN 1611-2156
Received: March 26, 2024, accepted: April 17, 2024, published: May 27, 2024

Table S3 (cont.) Summary SAR analysis of modified compounds in scaffold A

**Modified Series**	**Effect of structure modification** (Original VS Modified compounds)	**Most potent compound**	**Type / position of substitution**	**Influence of descriptor variables**
			○ Substitutions of X containing cyclohexane (**3A-14**) and cyclopentane (**3A-18**) at terminal of the chain gave compounds with decreased activity.<br>• When D ring is five-membered ring and ring B is closed<br>○ Only substitution of X containing pyridine ring gave compounds with improved activity (**3A-55**) while modification with other types of rings decreased activity when compared with the original compound **3A**.	
**4A**<br>(56 compounds)	Only two modified compounds with increased activity (**4A-45** and **4A-46**)<br>Other modified compounds = decreased activity<br><br>Subseries provided best activity = **subseries 4A-45 – 4A-48**	**4A-46**	Cyclopentane on terminal of X position<br>Y ring is change to pyridine	↑ R2v+<br>↓ nR05
			Changing type of ring D to six-membered ring decreased activity of the compounds (subseries **4A-45** – **4A-48** provided better activity than the related derivatives in subseries **4A-17** – **4A-20** i.e., **4A-45** > **4A-17**, **4A-46** > **4A-18**, **4A-47** > **4A-19**, **4A-48** > **4A-20**).<br>When ring D is five-membered ring, changing type of Y ring mostly gave the compounds with decreased activity (except for **4A-3** when Y is a pyridine ring).<br>When D ring is six-membered ring, the replacement of X benzene ring with aliphatic rings (cyclohexane and cyclopentane) and naphthalene ring leads to improved activity of the compounds (as observed for subseries **4A-17** – **4A-20**).	
**5A**<br>(39 compounds)	Most of the modified compounds exhibited improved activity when compared to the parent **5A**	**5A-33**	Additional benzene ring is substituted on X ring<br>Y ring is replaced by benzene ring	↓ Km<br>↑ R2v+
			The replacement of five-membered D ring to six-membered ring increase activity of the compounds (as observe when comparing **5A-2** > **5A-24**, **5A-3** > **5A-25**, **5A-4** > **5A-26**, **5A-21** > **5A-27**), except for the most potent compound **5A-33**.<br>The compounds in which their ring X is modified with longer alkyl chain or substitution of additional ring on X, as well as whose ring X is replaced by naphthalene provided better activities when compared to a parent compound **5A**. This was	

Table S3 (cont.) Summary SAR analysis of modified compounds in scaffold A

Modified Series	Effect of structure modification (Original VS Modified compounds)	Most potent compound	Type / position of substitution	Influence of descriptor variables
			observed for both derivatives in which their D ring is original five-membered ring and modified six-membered ring. Conversely, the modification of ring Y by various type of rings variously affected on bioactivity of the compounds.	
**6A** (287 compounds)	Most of the modified compounds exhibited improved activity when compared to the parent **6A**	**6A-116** **6A-115**	Ring D is changed to six-membered ring. A site is modified by longer substituted alkyl chain and insertion of terminal benzene ring. B site is modified to -COH group (only for **6A-116**). C site is substituted with $-OCH_3$ group (only for **6A-116**).	↑ R2v+ ↓ nR05 ↓ Km
		**6A-44** **6A-43** **6A-45**	Ring D is changed to six-membered ring. A site is modified by longer substituted alkyl chain and insertion of terminal benzene ring. B site is modified to -COH group (**6A-44**) or $-COCH_3$ group (**6A-45**). C site is substituted with $-OCH_3$ group	↑ R2v+ ↓ nR05 ↓ Km
		**6A-140**	Ring D is changed to six-membered ring. A site is modified by longer substituted alkyl chain and insertion of terminal naphthalene ring. B site is modified to -CO-benzyl group	↓ nR05 ↓ Km
		In each subseries minor modifications were performed on A, B, and C sites. The modified compounds are predicted with various bioactivity results, but most of them provided improved activities when compared to the parent compound **6A**. Replacement of ring D with six-membered ring is required for improved activity of the compounds. Modification on A site by insertion of terminal benzene ring is essential for preferable activity. Modification on B site by replacing the branched alkyl chain to -COH, $-COCH_3$, -CN increased activity of the compounds.		

EXCLI Journal 2024;23:818-832 – ISSN 1611-2156
Received: March 26, 2024, accepted: April 17, 2024, published: May 27, 2024

Table S3 (cont.) Summary SAR analysis of modified compounds in scaffold A

Modified Series	Effect of structure modification (Original VS Modified compounds)	Most potent compound	Type / position of substitution	Influence of descriptor variables
		Modification on C site by substitutions of $-OCH_3$ or $-OCF_3$ gave compounds with better activities than those substituted with -O-benzyl group.		
**Overview summary of modified scaffold A**		• Effects of replacement of five-membered ring D to six-membered ring are varied depending on the parent compounds. Improved activities were observed for the six-membered ring modified compounds series **5A-6A**, whereas decreased activities were found for those of the series **1A-4A**.<br>• Modified compounds in series **6A** provided the best predicted activities.<br>• Modified compounds in series **1A-2A** provided lesser activity than their parent compounds.<br>• Modified compounds in series **3A-6A** provided various predicted activities (both improved and impaired activities).		

Most of the modified compounds in **5A** subseries (Figure 4d) provided more potent activity (predicted pEC_{50} = 5.242-5.645, supplementary data, Scaffold A) than parent **5A** (experimental pEC_{50} = 5.410, Table 2). The high predicted activity of compound **5A-33** (predicted pEC_{50} = 5.645) could be due to the presence of benzene ring attached to O-atom on the X ring and the replacement of Y ring with the benzene ring. These modifications provided an increased van der Waals volume (R2v+) value together with a decreased Km value (supplementary information, Table S3). Considering the top-five most potent compounds, most of them, except for **5A-33**, are compounds in which their five-membered D rings were replaced with the six-membered ring (Figure 4d). From the whole modified compounds **5A** (39 compounds), it was indicated that activity of the modified compounds was improved when compared to the original **5A** if 1) their ring X are modified with longer alkyl chain or substituted with additional ring, or 2) their ring X is replaced by naphthalene. This was observed for both modified compounds whose maintain their original five-membered D ring as well as those whose D ring were modified to six-membered ring. Conversely, the modification of ring Y by various type of rings differently affected the bioactivity.

From the whole modified **4A** series (56 compounds), only two compounds (i.e., **4A-46** and **4A-45**, Figure 4c) provided the higher predicted pEC_{50} values (**4A-46** = 5.747, **4A-45** = 5.689, supplementary data, Scaffold A) than that of their parent **4A** (experimental pEC_{50}: **4A** = 5.660, Table 2). It was suggested that the best activity is achieved when cyclic aliphatic rings (i.e., cyclohexane or cyclopentane) and the pyridine ring were replaced at rings X and Y, respectively. Conversely, the replacement of the five-membered ring D with the six-membered ring leads to compounds with impaired activity.

From the modified series **3A**, the best activity was obtained when the ring D as well as the moiety at C position are retained in the original form as their parent **3A** (Figure 4b). Moreover, an opening of ring B with terminal $-OCH_3$ substitutions on Y position together an insertion of alkyl chain linker with terminal pyridine ring on ring X leads to compound **3A-78**

with the most improved activity (predicted pEC_{50}: **3A-78** = 5.734 and **3A** = 5.620). Additionally, it was found from the modified series **1A** and **2A** that the structural features that lead to an impaired S1PR2 modulating activity includes an opening of ring B, an increasing length of the alkyl chain on X position, as well as the replacement of ring D with six-membered ring.

Section 2: Summary SAR analysis scaffold B

Section 2.1: Original set (1B-5B)

- Activity: **4B** >**3B** > **2B** > **5B** > **1B**
- Note: **5B** different structure (not use for comparison)

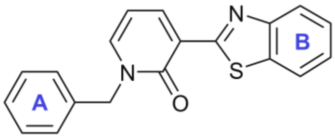

Original compounds
1B - 4B

Table S4 Summary SAR analysis of scaffold B original compounds (**1B-5B**)

Activity comparison	Effects of type / position / numbers of substitution on ring A	Effects on activity (increase / decrease)	Influence of descriptor variables
**4B** > **3B** > **2B** > **1B**	**2 Cl > 1 Cl > CH_3 > H**	Halogen substitution provides better activity than alkyl / hydrogen group	Changing type of R groups influence values of both descriptors
**4B** > **3B**	**2 Cl > 1 Cl**	Addition of more chlorine group on A ring increase activity via increasing van der Waals volume	via increasing H3e value

Section 2.2: Modified compounds (117 compounds)

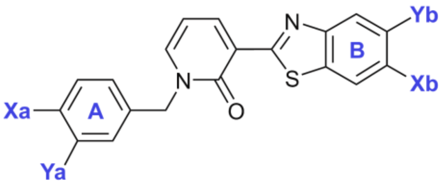

Modified compounds **1B - 4B**

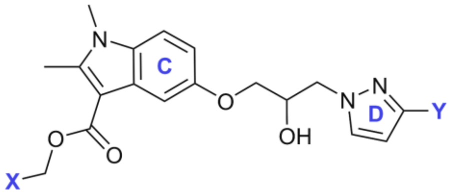

Modified compounds **5B**

EXCLI Journal 2024;23:818-832 – ISSN 1611-2156
Received: March 26, 2024, accepted: April 17, 2024, published: May 27, 2024

Table S5 Summary SAR analysis of modified compounds in scaffold B

**Modified Series**	**Effect of structure modification** (Original VS Modified compounds)	**Most potent compound**	**Type / position of substitution**	**Influence of descriptor variables**
**1B** (28 compounds)	All modified compounds = improved activity (**1B-3** > **1B-4** > **1B-8** > **1B-6**)	**1B-3** (Ring A)	Xa = 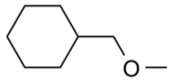	High H3e
		**1B-4** (Ring A)	Xa = 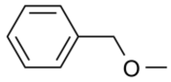	High E3v
	Modification of Ring A (**1B-1** – **1B-16**)	Modification on ring A (pEC_{50} = 5.739-7.977) gave compounds with improved activity than modification on ring B (pEC_{50} = 5.403-6.043)		
	Modification of Ring B (**1B-17** – **1B-28**)	Among compounds with modification on ring B, the compounds modified with NO_2 group gave the most promising improved activities (pEC_{50} = 5.752-6.043) when compared with those of -OH, $-OCH_3$, $-OCF_3$, and halogen series		
		Di-substitution of the same moiety on ring B (X_b=Y_b) gave the best activity when compared to substitution with different type of group, except for the series $-OCH_3$.<br>OCH_3 series: X_b= NO_2 > OCF_3 > OH > OCH_3 (**1B-16** > **1B-15** > **1B-14** > **1B-13**)<br>OCF_3 series: X_b= OCF_3 > NO_2 > OH > OCH_3 (**1B-17** > **1B-20** > **1B-18** > **1B-19**)<br>NO_2 series: X_b= NO_2 > OH > OCF_3 > OCH_3 (**1B-21** > **1B-22** > **1B-24** > **1B-23**)		
		For halogen series, the best activity was obtained when Y_b is substituted with fluoride (F) atom: F > Cl and Br > I (**1B-25** > **1B-26** ≈ **1B-27** > **1B-28**)		
**2B** (4 compounds)	All modified compounds = improved activity<br>Modification of Ring A<br>(**2B-3** > **2B-4** > **2B-2** > **2B-1**)	**2B-3**	Xa = 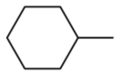	High H3e and E3V values
		Type of substitution affected values of electronegativity and van der Waal descriptors.<br>Ring substitution (**2B-3** and **2B-4**) gave compounds with better activities than alkyl-chain substitution (**2B-2** and **2B-1**)		

EXCLI Journal 2024;23:818-832 – ISSN 1611-2156
Received: March 26, 2024, accepted: April 17, 2024, published: May 27, 2024

Table S5 (cont.) Summary SAR analysis of modified compounds in scaffold B

Modified Series	Effect of structure modification (Original VS Modified compounds)	Most potent compound	Type / position of substitution	Influence of descriptor variables
**3B**<br>(11 compounds)	All modified compounds = improved activity<br>(**3B-4** > **3B-5** > **3B-6** > **3B-7**)<br>Modification of Ring A only<br>(**3B-1** – **3B-3**)<br>Modification of Ring A and B<br>Series Ia: when Xa = cycloalkane, Yb = halogen<br>(**3B-4** – **3B-7**)<br>Series IIa: when Xa = benzene ring (**3B-8** – **3B-11**)	Modifications on both ring A and B can better improve activities of the compounds than modification on ring A alone.		
		**3B-4**	Xa = 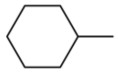	High H3e values
		Substitution on ring A by replacing Cl-group with six-membered aliphatic ring (series Ia cycloalkane) affected the increasing values of both descriptors and provided modified compounds with better activity than the substitution with aromatic ring (series IIa benzene)		
		Examples:<br>**3B-4** > **3B-8**<br>**3B-5** > **3B-9**<br>**3B-6** > **3B-10**<br>**3B-7** > **3B-11**	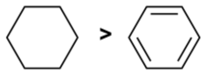	Increased H3e and E3V values
		Type of halogen substitution on ring B (at Yb) affected activities of the compounds.<br>Fluorine (F) substitution gave the best activity for compounds in series Ia (F > Cl > Br > I: **3B-4** > **3B-5** > **3B-6** > **3B-7**) whereas the iodine (I) substitution gave the most potent compound for series IIa (I > F > Cl > Br: **3B-11** > **3B-8** > **3B-9** > **3B-10**).		
**4B**<br>(63 compounds)	All modified compounds = improved activity<br>(**4B-48** > **4B-53** > **4B-52** > **4B-54**)<br>Modification of Ring A only<br>F-series: Xa = F, Xb = vary R<br>(**4B-1** – **4B-8**)<br>Cl-series: Xa = Cl, Xb = vary R<br>(**4B-9** – **4B-15**)<br>Br-series: Xa = Br, Xb = vary R<br>(**4B-16** – **4B-23**)<br>I-series: Xa = I, Xb = vary R<br>(**4B-24** – **4B-31**)	**4B-48**	Xa = OCF_3<br>Ya = F	High H3e and E3v values
		Modified compounds of OCF_3-series provided the most improved activities (predicted pEC_{50} = 6.275-6.795).<br>Potent predicted activities of the compounds in OCF_3-series are due to their high E3v value (all compounds possess E3v value > 3, greater than those of other series).<br>It was observed that all the most potent compounds of each subseries possess the highest H3e value (and some of them provide the highest E3v value) among others in the same subseries.<br>In overview, structural modifications could improve activities of the compounds mostly via affecting the H3e value.		

Table S5 (cont.) Summary SAR analysis of modified compounds in scaffold B

Modified Series	Effect of structure modification (Original VS Modified compounds)	Most potent compound	Type / position of substitution	Influence of descriptor variables
	CF_3-series: Xa = CF_3, Xb = vary R (**4B-32** – **4B-39**) OCH_3-series: Xa = OCH_3, Xb = vary R (**4B-40** – **4B-47**) OCF_3-series: Xa = OCF_3, Xb = vary R (**4B-48** – **4B-55**) NO_2-series: Xa = NO_2, Xb = vary R (**4B-56** – **4B-63**)			
**5B** (11 compounds)	All modified compounds = improved activity (**5B-9** > **5B-7** > **5B-11** > **5B-5**) Modification of Ring C only by changing X (**5B-1** – **5B-3**) Modification of Ring C and D by changing X and Y (**5B-4** – **5B-11**)	**5B-9**	X = CH_2CH_3 Y = branched tri-CH_3	
		Replacement of X with longer alkyl chain, branched alkyl group, and ring increased activities of the modified compounds. In overview, the best improved activity was obtained when X is replaced with long alkyl chain and Y is replaced with branched alkyl chain.		

EXCLI Journal 2024;23:818-832 – ISSN 1611-2156
Received: March 26, 2024, accepted: April 17, 2024, published: May 27, 2024

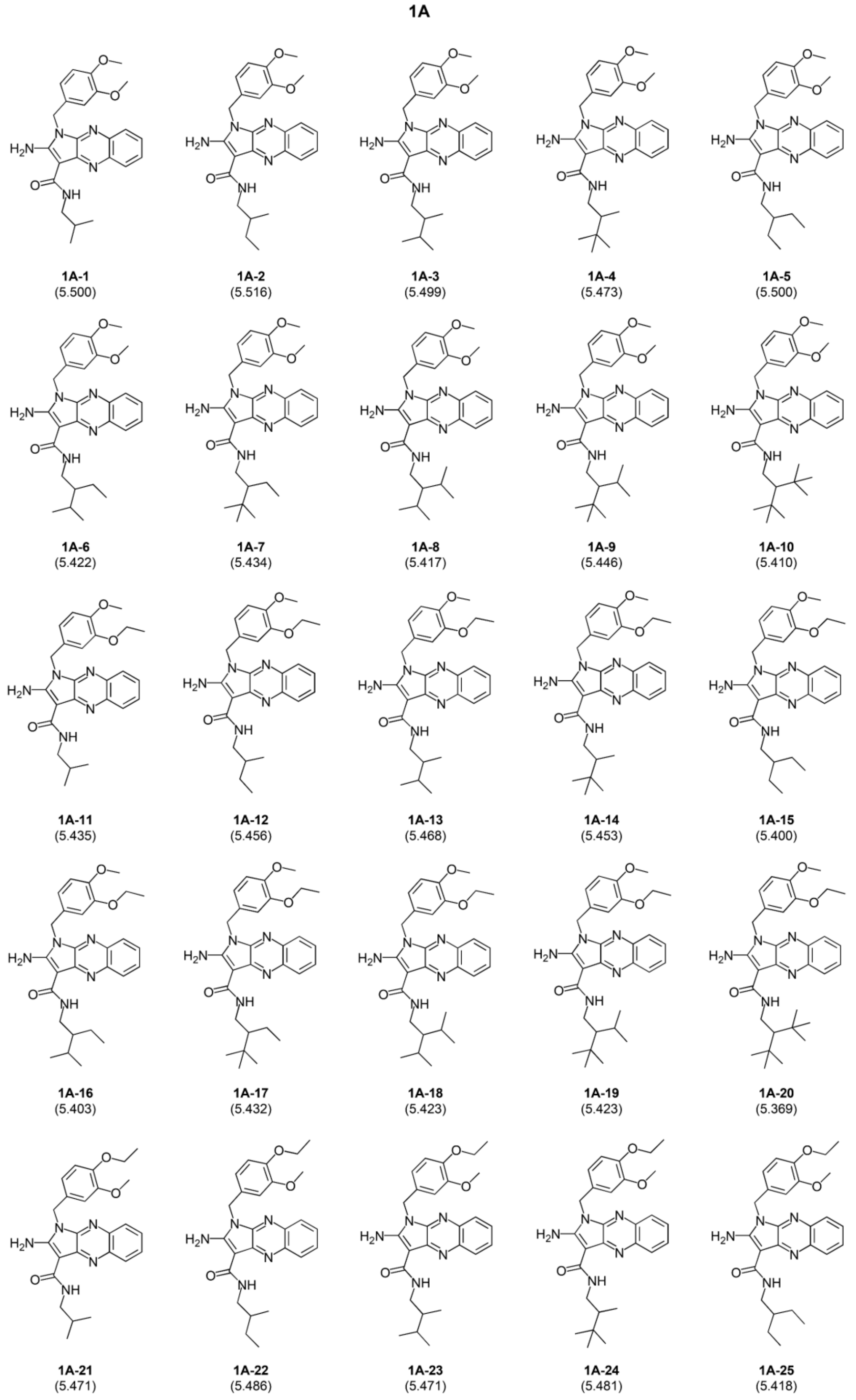

Figure S1: Chemical structure of modified compounds **1A**

EXCLI Journal 2024;23:818-832 – ISSN 1611-2156
Received: March 26, 2024, accepted: April 17, 2024, published: May 27, 2024

1A

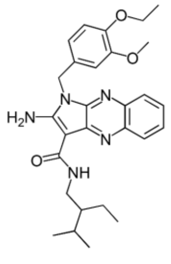

1A-26
(5.424)

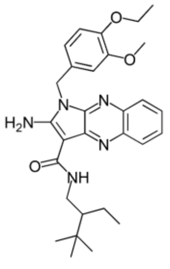

1A-27
(5.433)

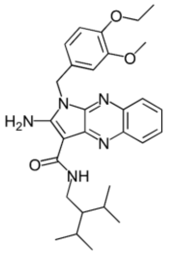

1A-28
(5.423)

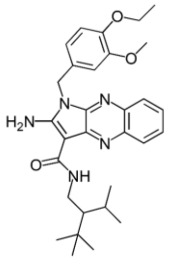

1A-29
(5.442)

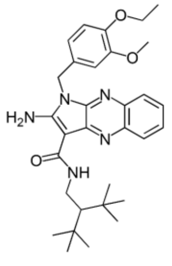

1A-30
(5.380)

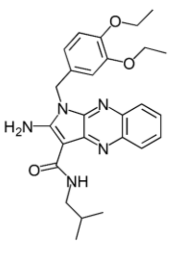

1A-31
(5.412)

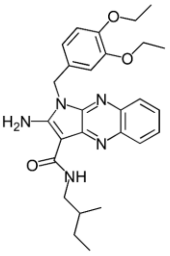

1A-32
(5.430)

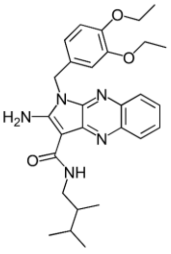

1A-33
(5.442)

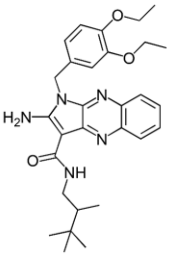

1A-34
(5.429)

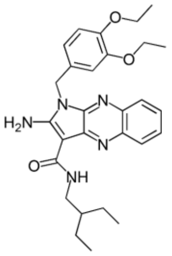

1A-35
(5.388)

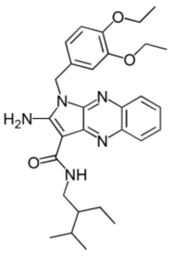

1A-36
(5.369)

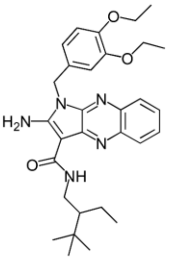

1A-37
(5.399)

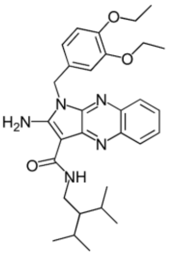

1A-38
(5.389)

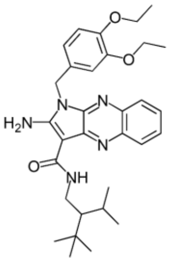

1A-39
(5.392)

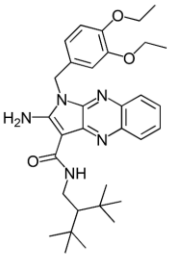

1A-40
(5.353)

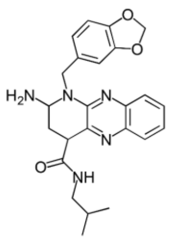

1A-41
(5.429)

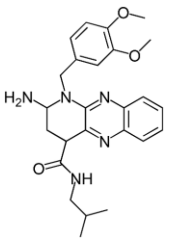

1A-42
(5.537)

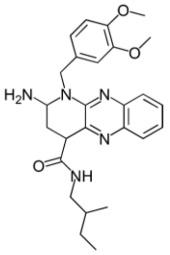

1A-43
(5.363)

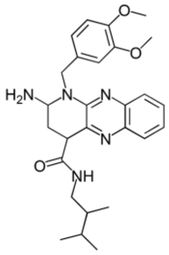

1A-44
(5.328)

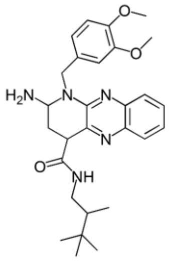

1A-45
(5.465)

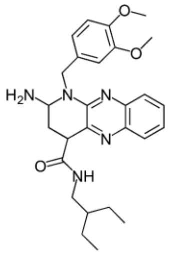

1A-46
(5.374)

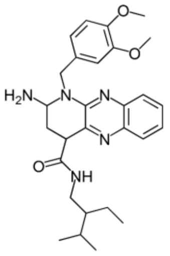

1A-47
(5.517)

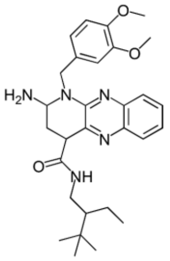

1A-48
(5.327)

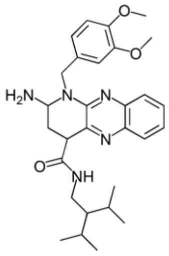

1A-49
(5.479)

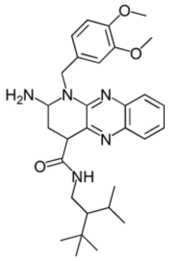

1A-50
(5.315)

Figure S1 (cont.): Chemical structure of modified compounds **1A**

EXCLI Journal 2024;23:818-832 – ISSN 1611-2156
Received: March 26, 2024, accepted: April 17, 2024, published: May 27, 2024

1A

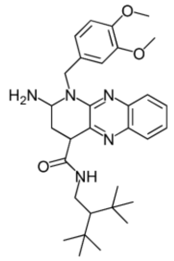
1A-51
(5.315)

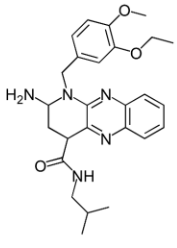
1A-52
(5.366)

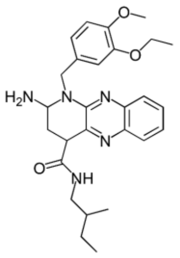
1A-53
(5.331)

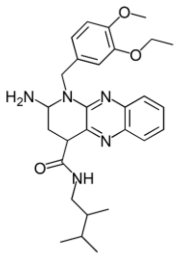
1A-54
(5.297)

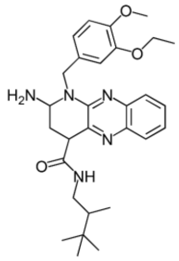
1A-55
(5.422)

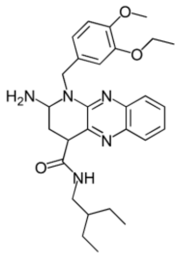
1A-56
(5.364)

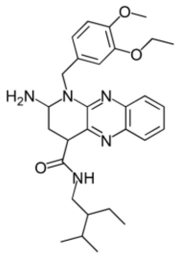
1A-57
(5.467)

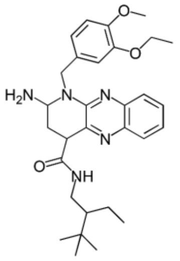
1A-58
(5.292)

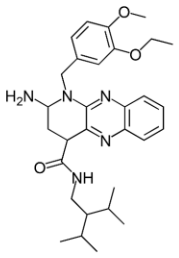
1A-59
(5.455)

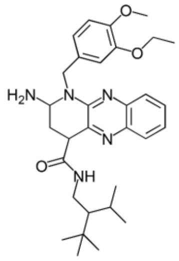
1A-60
(5.285)

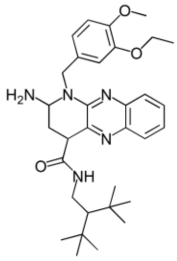
1A-61
(5.285)

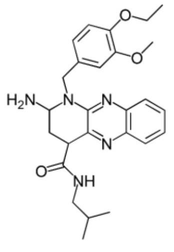
1A-62
(5.374)

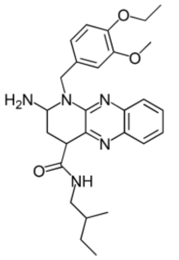
1A-63
(5.341)

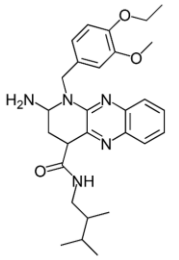
1A-64
(5.308)

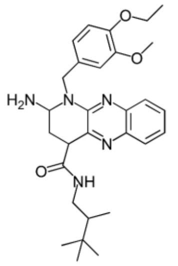
1A-65
(5.439)

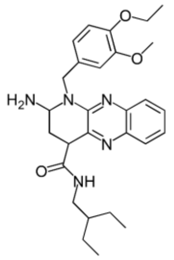
1A-66
(5.492)

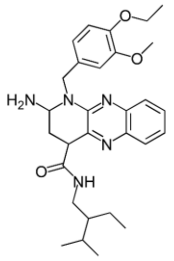
1A-67
(5.489)

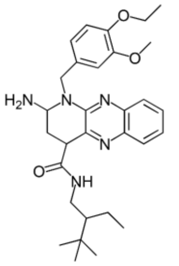
1A-68
(5.265)

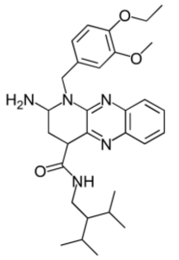
1A-69
(5.453)

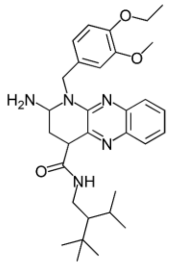
1A-70
(5.271)

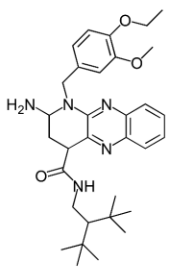
1A-71
(5.270)

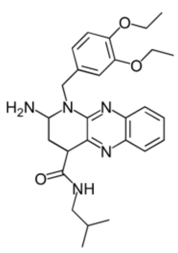
1A-72
(5.322)

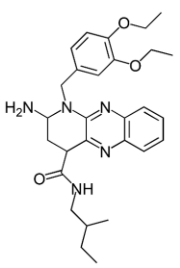
1A-73
(5.289)

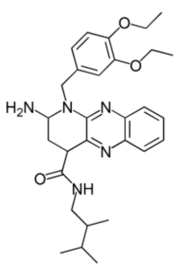
1A-74
(5.256)

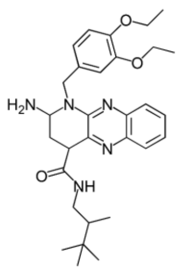
1A-75
(5.413)

Figure S1 (cont.): Chemical structure of modified compounds **1A**

EXCLI Journal 2024;23:818-832 – ISSN 1611-2156
Received: March 26, 2024, accepted: April 17, 2024, published: May 27, 2024

1A

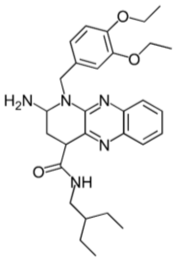

1A-76
(5.361)

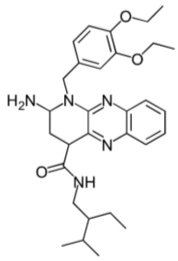

1A-77
(5.440)

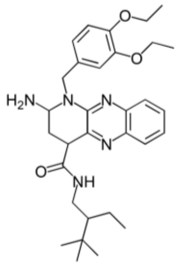

1A-78
(5.275)

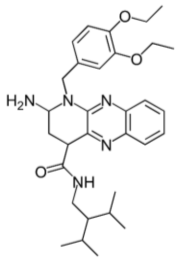

1A-79
(5.431)

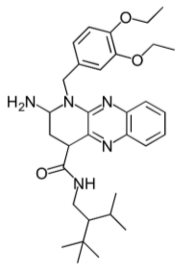

1A-80
(5.244)

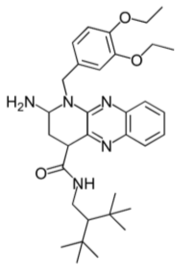

1A-81
(5.464)

Figure S1 (cont.): Chemical structure of modified compounds **1A**

EXCLI Journal 2024;23:818-832 – ISSN 1611-2156
Received: March 26, 2024, accepted: April 17, 2024, published: May 27, 2024

2A

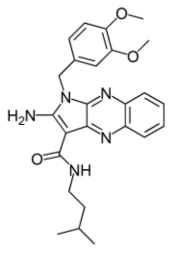
2A-1 (5.484)

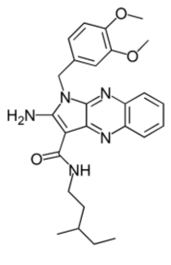
2A-2 (5.472)

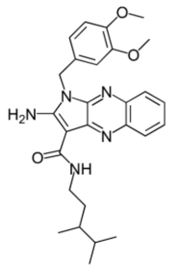
2A-3 (5.435)

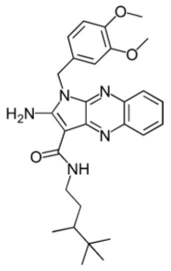
2A-4 (5.462)

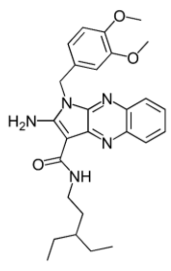
2A-5 (5.444)

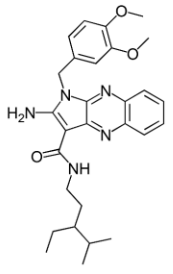
2A-6 (5.452)

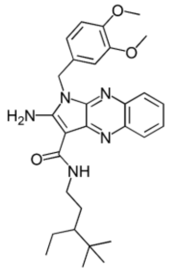
2A-7 (5.413)

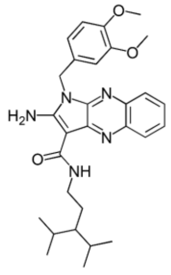
2A-8 (5.411)

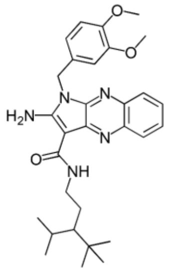
2A-9 (5.405)

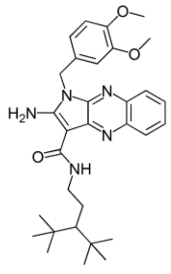
2A-10 (5.357)

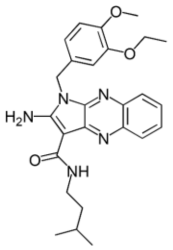
2A-11 (5.454)

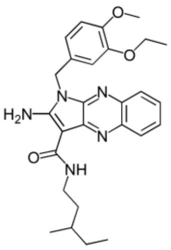
2A-12 (5.449)

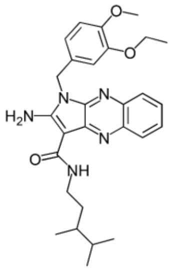
2A-13 (5.448)

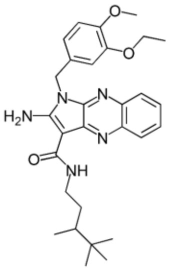
2A-14 (5.437)

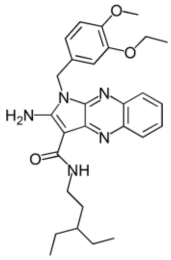
2A-15 (5.396)

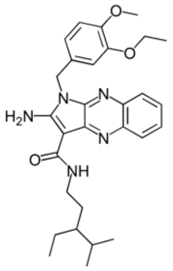
2A-16 (5.434)

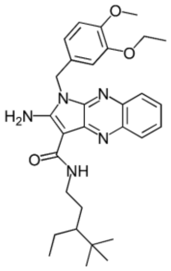
2A-17 (5.464)

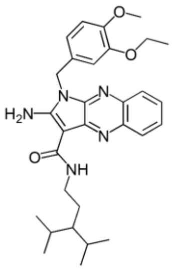
2A-18 (5.395)

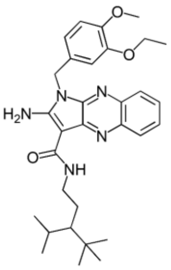
2A-19 (5.391)

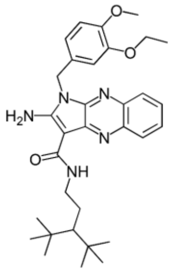
2A-20 (5.341)

Figure S2: Chemical structure of modified compounds **2A**

EXCLI Journal 2024;23:818-832 – ISSN 1611-2156
Received: March 26, 2024, accepted: April 17, 2024, published: May 27, 2024

2A

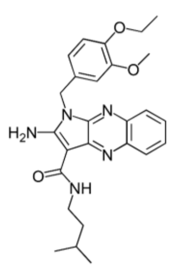

2A-21
(5.460)

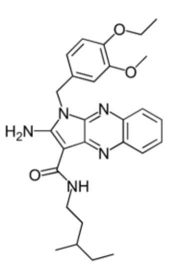

2A-22
(5.453)

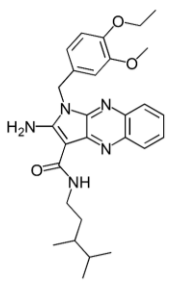

2A-23
(5.438)

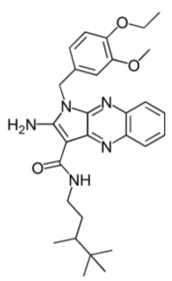

2A-24
(5.463)

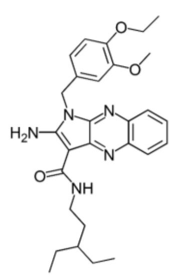

2A-25
(5.428)

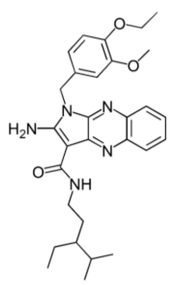

2A-26
(5.406)

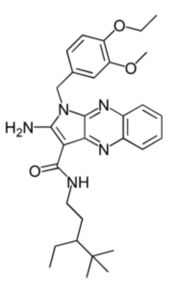

2A-27
(5.390)

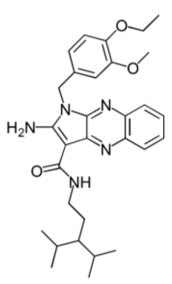

2A-28
(5.472)

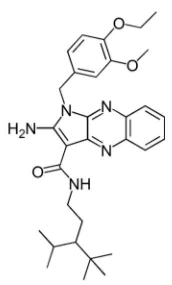

2A-29
(5.395)

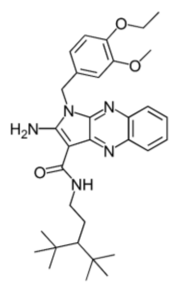

2A-30
(5.348)

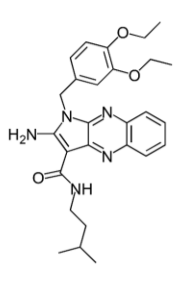

2A-31
(5.432)

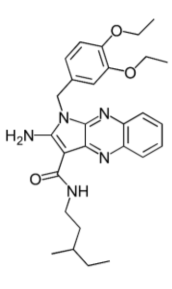

2A-32
(5.428)

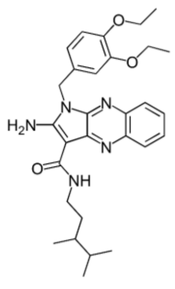

2A-33
(5.418)

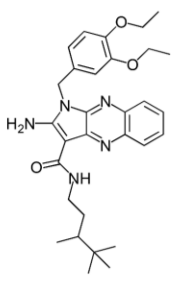

2A-34
(5.441)

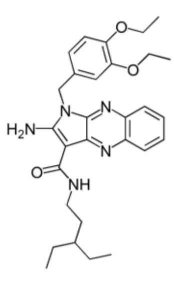

2A-35
(5.378)

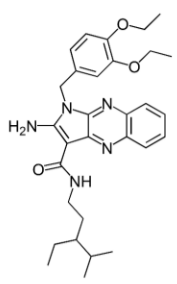

2A-36
(5.418)

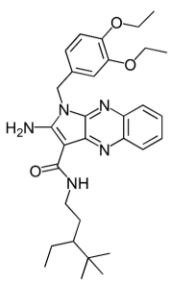

2A-37
(5.461)

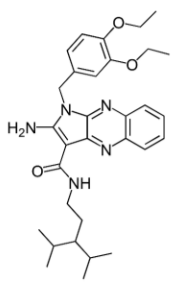

2A-38
(5.381)

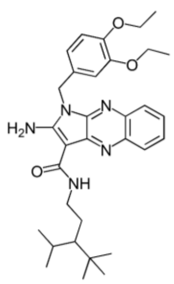

2A-39
(5.350)

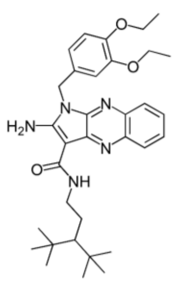

2A-40
(5.330)

Figure S2 (cont.): Chemical structure of modified compounds **2A**

EXCLI Journal 2024;23:818-832 – ISSN 1611-2156
Received: March 26, 2024, accepted: April 17, 2024, published: May 27, 2024

2A

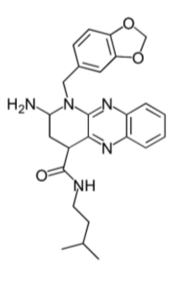

2A-41
(5.405)

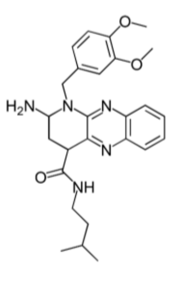

2A-42
(5.377)

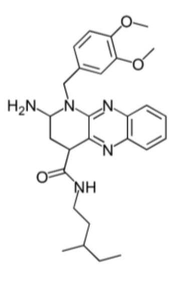

2A-43
(5.360)

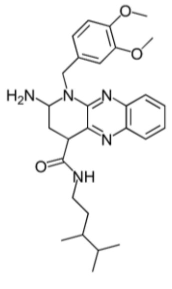

2A-44
(5.317)

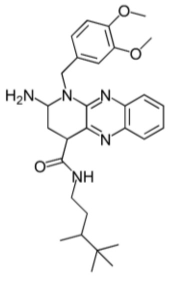

2A-45
(5.282)

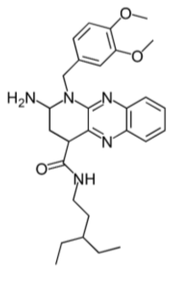

2A-46
(5.351)

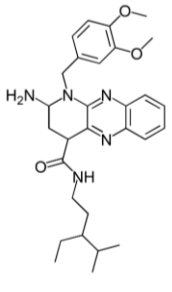

2A-47
(5.377)

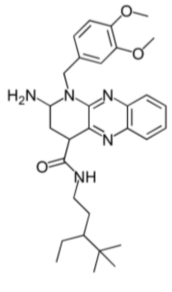

2A-48
(5.421)

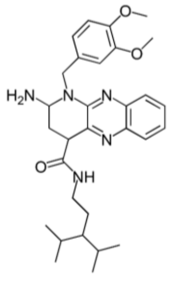

2A-49
(5.265)

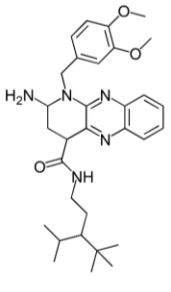

2A-50
(5.337)

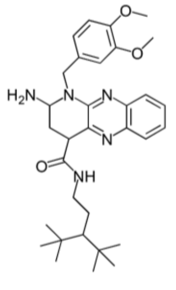

2A-51
(5.310)

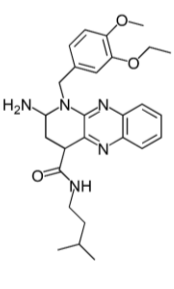

2A-52
(5.347)

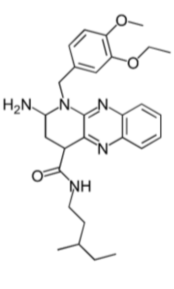

2A-53
(5.332)

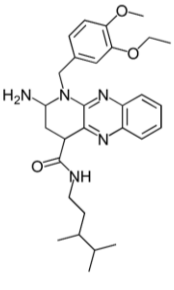

2A-54
(5.289)

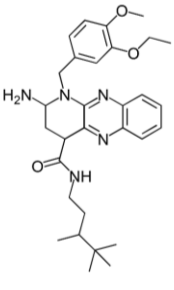

2A-55
(5.254)

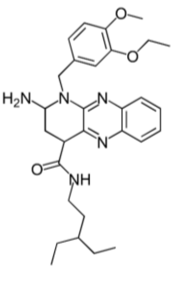

2A-56
(5.298)

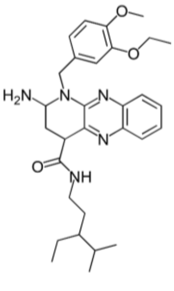

2A-57
(5.326)

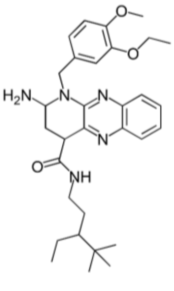

2A-58
(5.399)

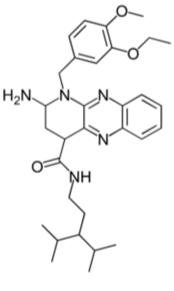

2A-59
(5.430)

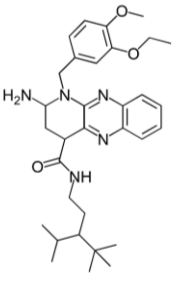

2A-60
(5.511)

Figure S2 (cont.): Chemical structure of modified compounds **2A**

EXCLI Journal 2024;23:818-832 – ISSN 1611-2156
Received: March 26, 2024, accepted: April 17, 2024, published: May 27, 2024

2A

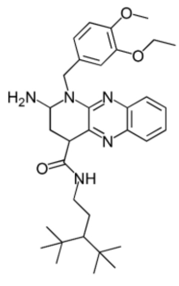
2A-61
(5.429)

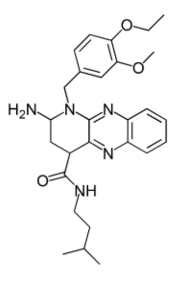
2A-62
(5.355)

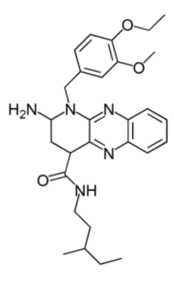
2A-63
(5.340)

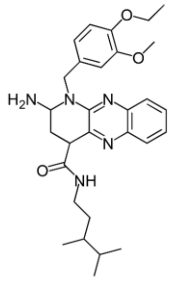
2A-64
(5.300)

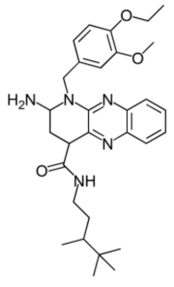
2A-65
(5.242)

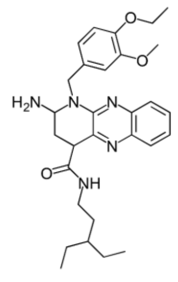
2A-66
(5.308)

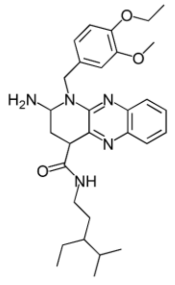
2A-67
(5.334)

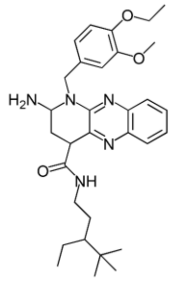
2A-68
(5.401)

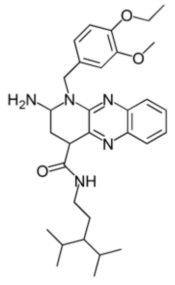
2A-69
(5.406)

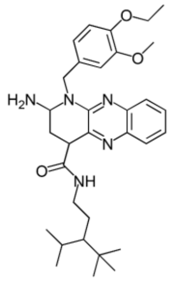
2A-70
(5.459)

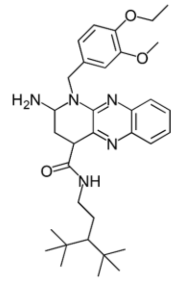
2A-71
(5.426)

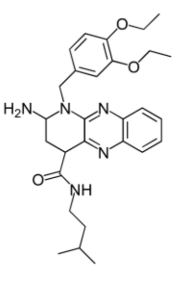
2A-72
(5.499)

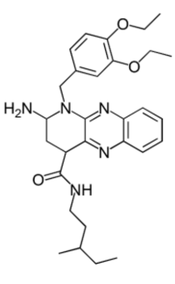
2A-73
(5.291)

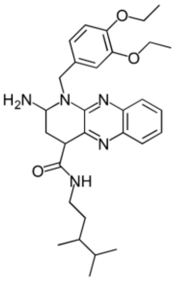
2A-74
(5.250)

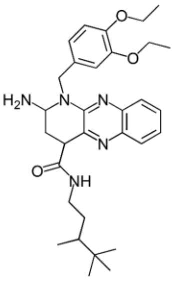
2A-75
(5.387)

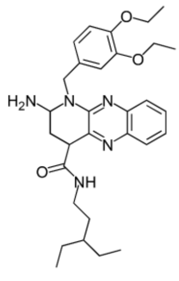
2A-76
(5.283)

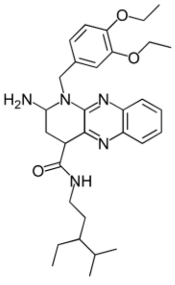
2A-77
(5.286)

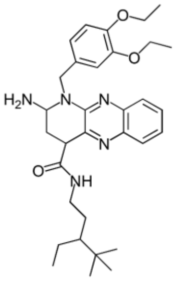
2A-78
(5.356)

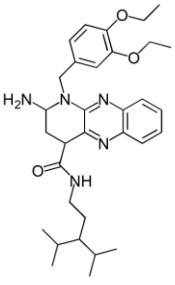
2A-79
(5.414)

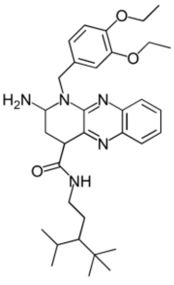
2A-80
(5.266)

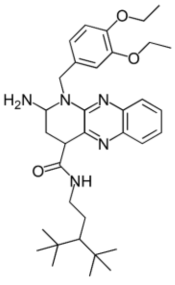
2A-81
(5.243)

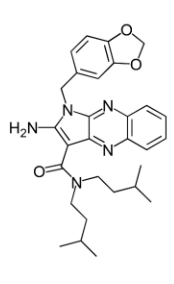
2A-82
(5.476)

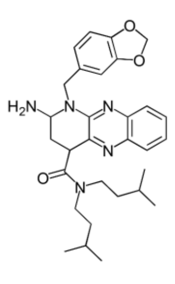
2A-83
(5.430)

Figure S2 (cont.): Chemical structure of modified compounds **2A**

EXCLI Journal 2024;23:818-832 – ISSN 1611-2156
Received: March 26, 2024, accepted: April 17, 2024, published: May 27, 2024

3A

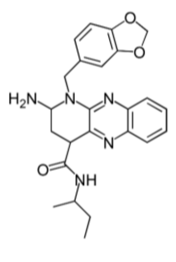

3A-1
(5.489)

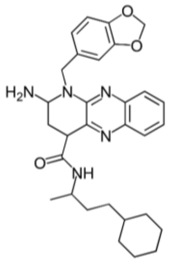

3A-2
(5.453)

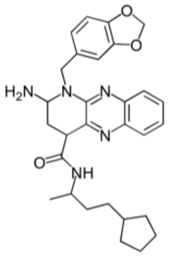

3A-3
(5.388)

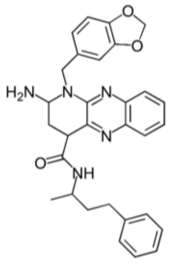

3A-4
(5.537)

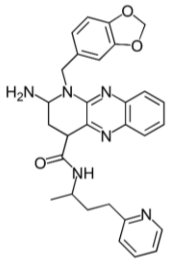

3A-5
(5.514)

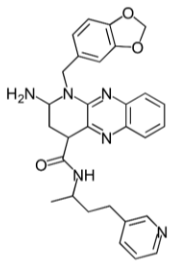

3A-6
(5.482)

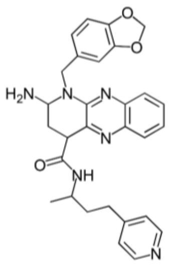

3A-7
(5.526)

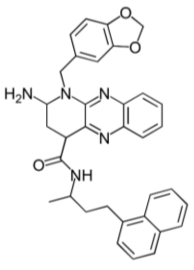

3A-8
(5.653)

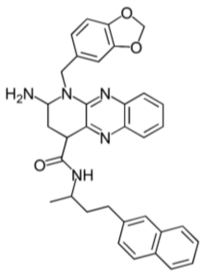

3A-9
(5.474)

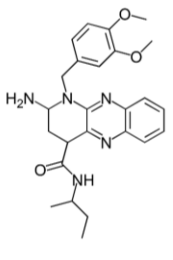

3A-10
(5.482)

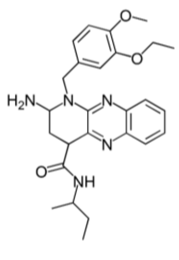

3A-11
(5.409)

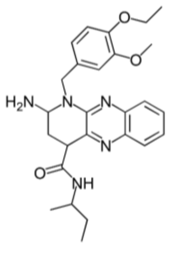

3A-12
(5.414)

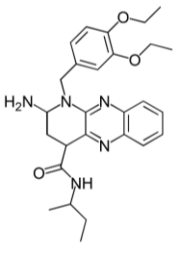

3A-13
(5.362)

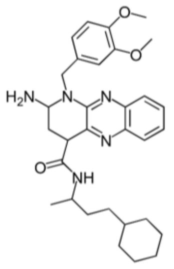

3A-14
(5.429)

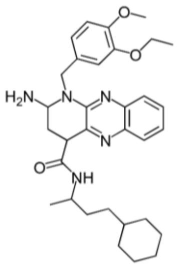

3A-15
(5.408)

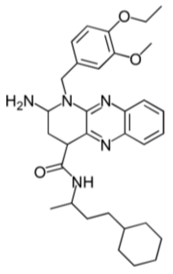

3A-16
(5.430)

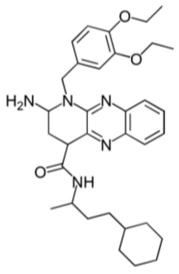

3A-17
(5.384)

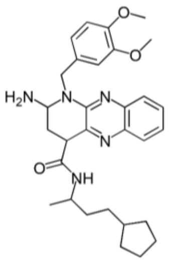

3A-18
(5.392)

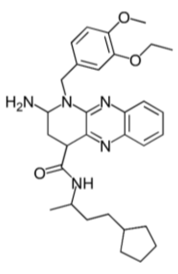

3A-19
(5.377)

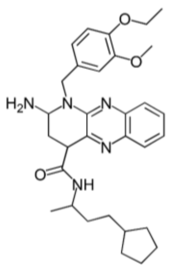

3A-20
(5.368)

Figure S3: Chemical structure of modified compounds **3A**

EXCLI Journal 2024;23:818-832 – ISSN 1611-2156
Received: March 26, 2024, accepted: April 17, 2024, published: May 27, 2024

3A

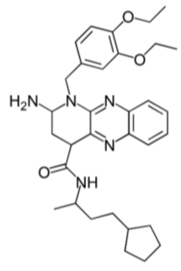
3A-21
(5.351)

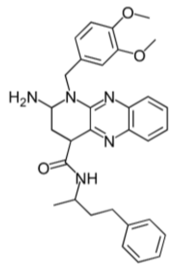
3A-22
(5.534)

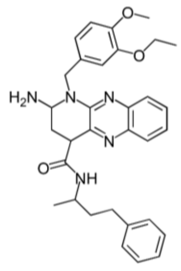
3A-23
(5.432)

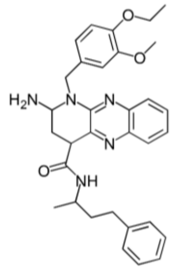
3A-24
(5.453)

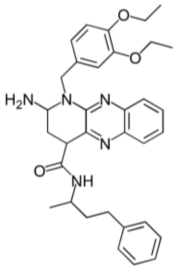
3A-25
(5.417)

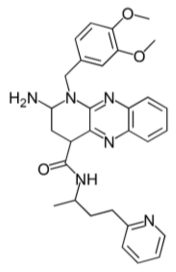
3A-26
(5.535)

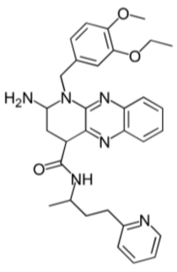
3A-27
(5.537)

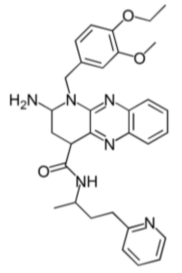
3A-28
(5.509)

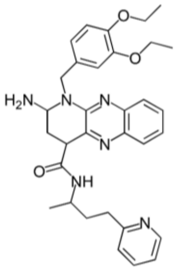
3A-29
(5.512)

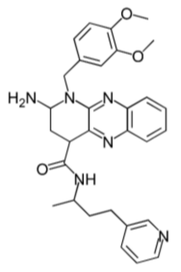
3A-30
(5.503)

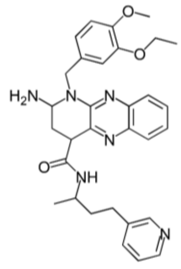
3A-31
(5.506)

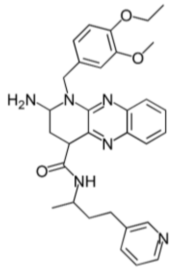
3A-32
(5.478)

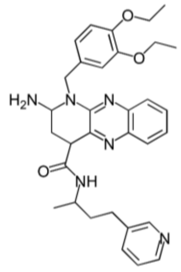
3A-33
(5.481)

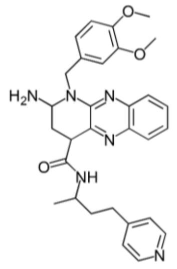
3A-34
(5.636)

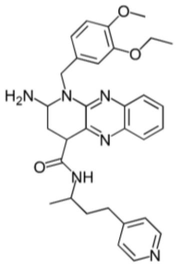
3A-35
(5.537)

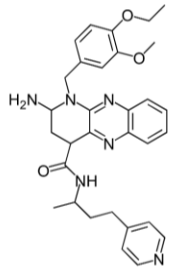
3A-36
(5.506)

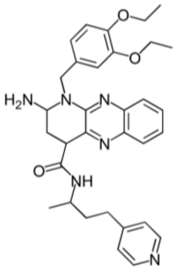
3A-37
(5.490)

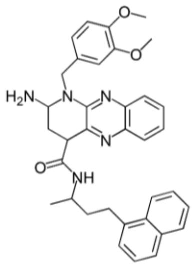
3A-38
(5.531)

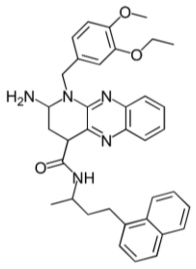
3A-39
(5.426)

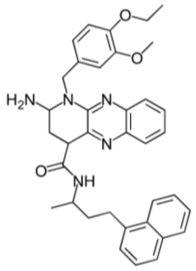
3A-40
(5.425)

Figure S3 (cont.): Chemical structure of modified compounds **3A**

3A

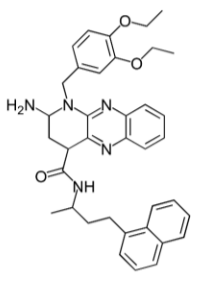

3A-41
(5.427)

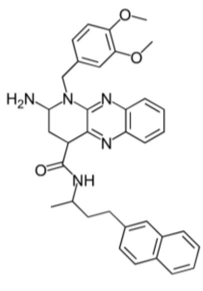

3A-42
(5.474)

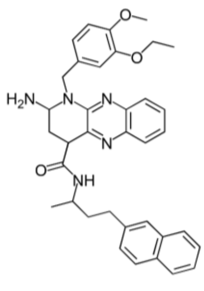

3A-43
(5.480)

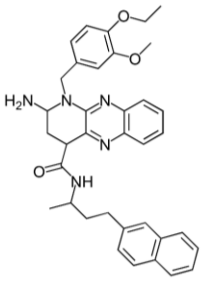

3A-44
(5.456)

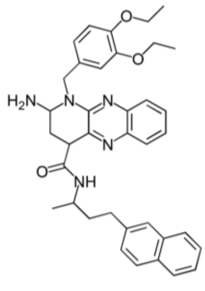

3A-45
(5.460)

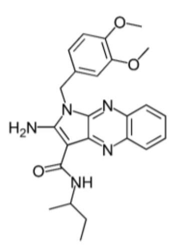

3A-46
(5.512)

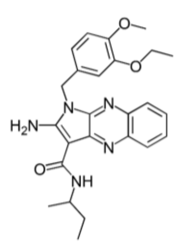

3A-47
(5.441)

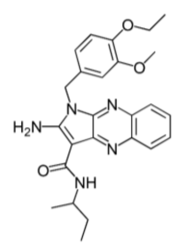

3A-48
(5.481)

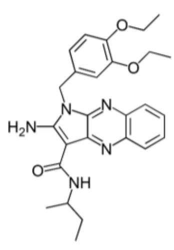

3A-49
(5.418)

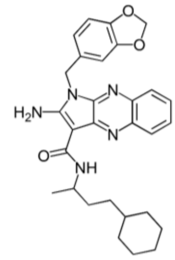

3A-50
(5.554)

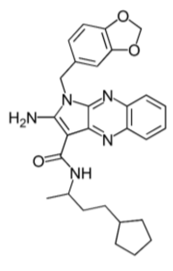

3A-51
(5.530)

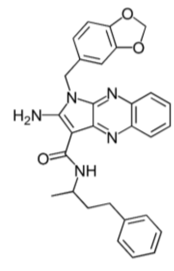

3A-52
(5.546)

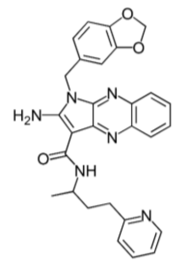

3A-53
(5.556)

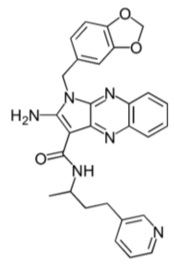

3A-54
(5.550)

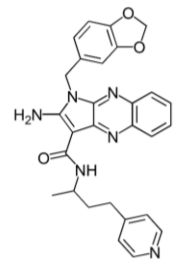

3A-55
(5.655)

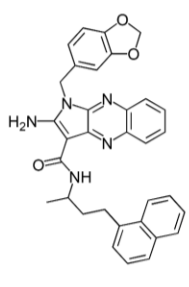

3A-56
(5.577)

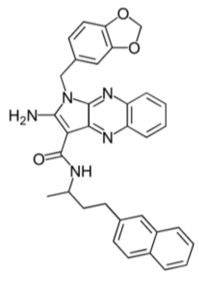

3A-57
(5.502)

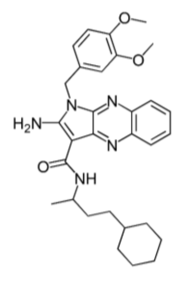

3A-58
(5.478)

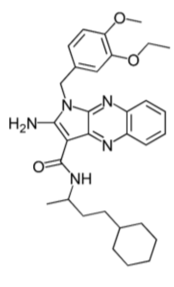

3A-59
(5.460)

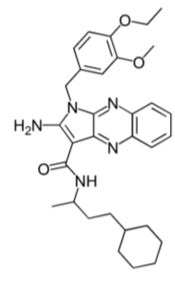

3A-60
(5.470)

Figure S3 (cont.): Chemical structure of modified compounds **3A**

EXCLI Journal 2024;23:818-832 – ISSN 1611-2156
Received: March 26, 2024, accepted: April 17, 2024, published: May 27, 2024

3A

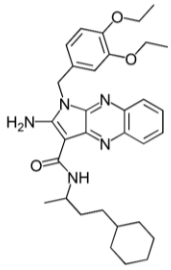

3A-61
(5.448)

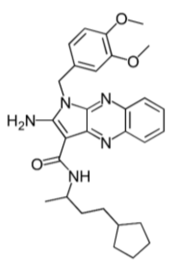

3A-62
(5.519)

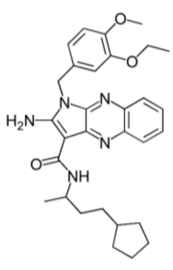

3A-63
(5.487)

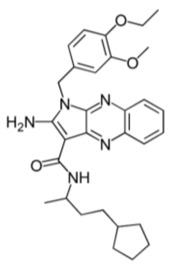

3A-64
(5.516)

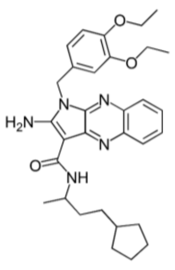

3A-65
(5.466)

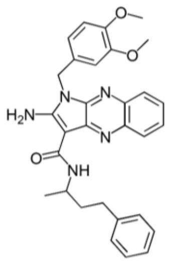

3A-66
(5.551)

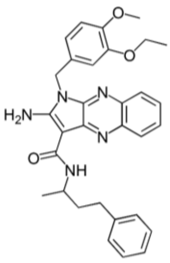

3A-67
(5.541)

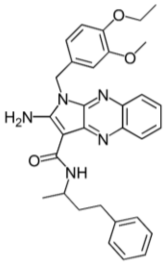

3A-68
(5.547)

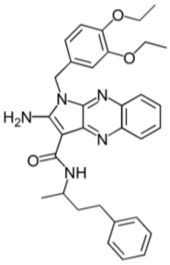

3A-69
(5.587)

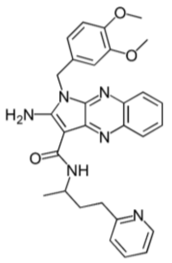

3A-70
(5.610)

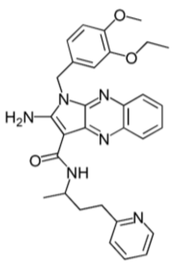

3A-71
(5.577)

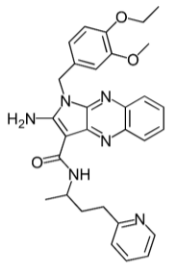

3A-72
(5.581)

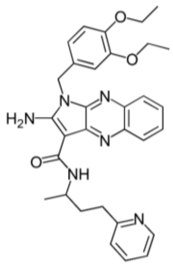

3A-73
(5.577)

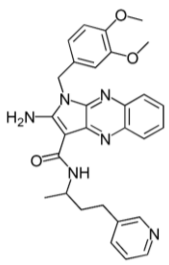

3A-74
(5.606)

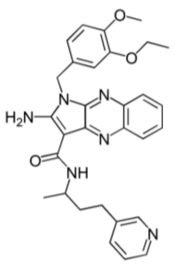

3A-75
(5.597)

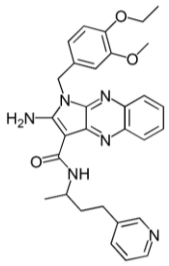

3A-76
(5.576)

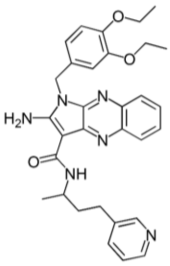

3A-77
(5.574)

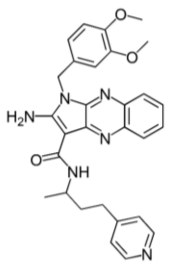

3A-78
(5.734)

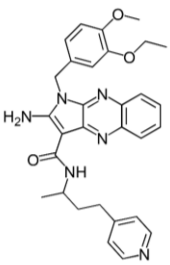

3A-79
(5.704)

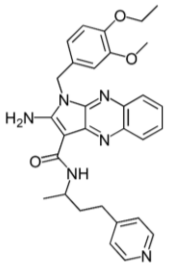

3A-80
(5.705)

Figure S3 (cont.): Chemical structure of modified compounds **3A**

EXCLI Journal 2024;23:818-832 – ISSN 1611-2156
Received: March 26, 2024, accepted: April 17, 2024, published: May 27, 2024

3A

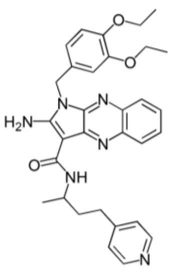

3A-81
(5.677)

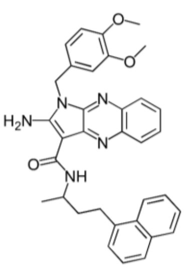

3A-82
(5.543)

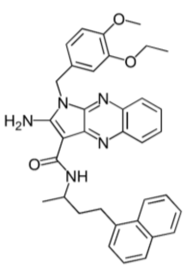

3A-83
(5.498)

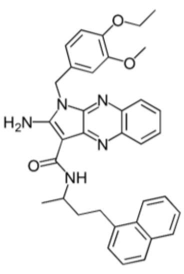

3A-84
(5.541)

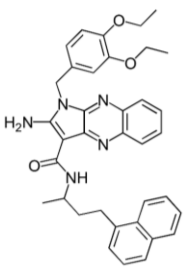

3A-85
(5.490)

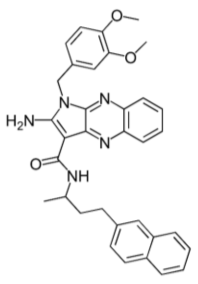

3A-86
(5.487)

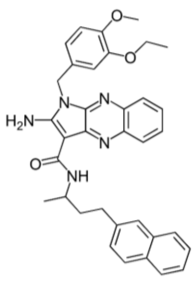

3A-87
(5.455)

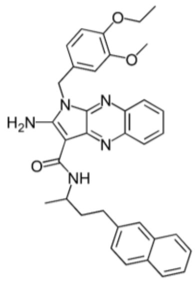

3A-88
(5.496)

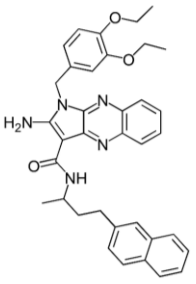

3A-89
(5.449)

Figure S3 (cont.): Chemical structure of modified compounds **3A**

EXCLI Journal 2024;23:818-832 – ISSN 1611-2156
Received: March 26, 2024, accepted: April 17, 2024, published: May 27, 2024

4A

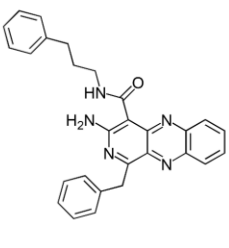

4A-1
(5.453)

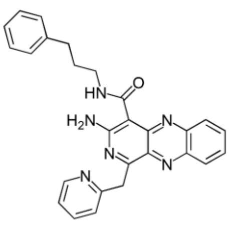

4A-2
(5.409)

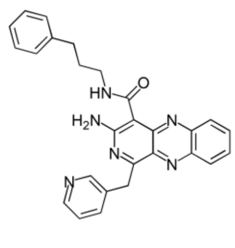

4A-3
(5.477)

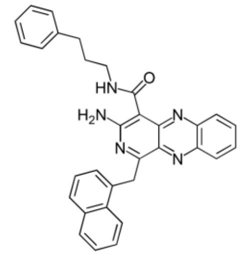

4A-4
(5.379)

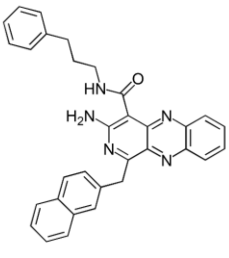

4A-5
(5.453)

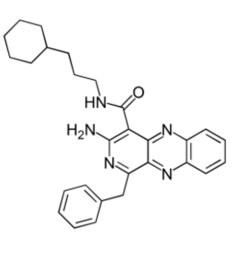

4A-6
(5.524)

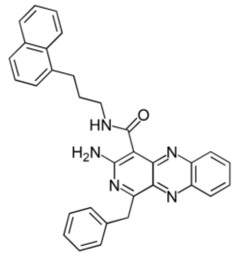

4A-7
(5.377)

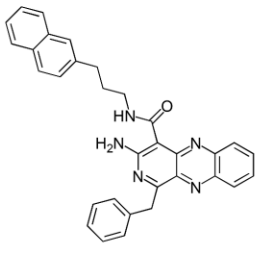

4A-8
(5.304)

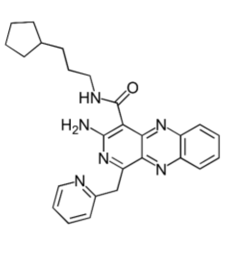

4A-9
(5.390)

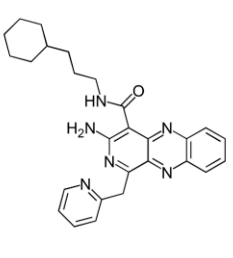

4A-10
(5.370)

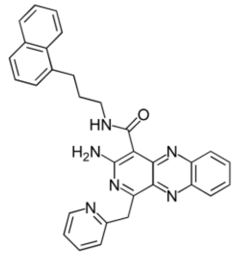

4A-11
(5.297)

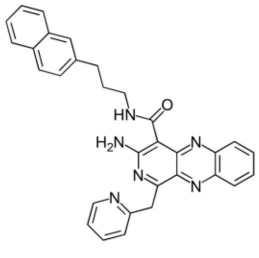

4A-12
(5.322)

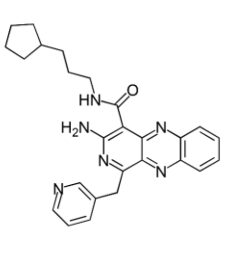

4A-13
(5.413)

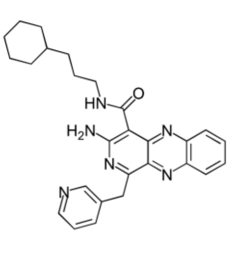

4A-14
(5.399)

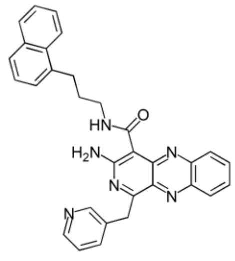

4A-15
(5.353)

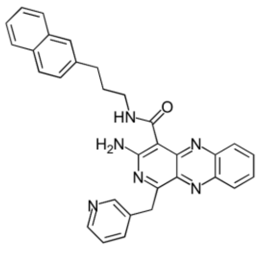

4A-16
(5.419)

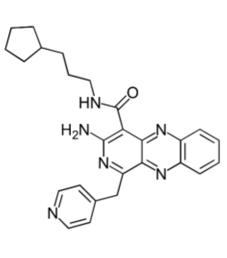

4A-17
(5.554)

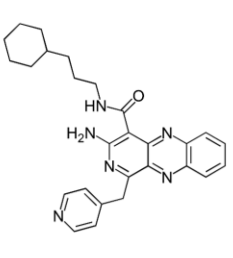

4A-18
(5.565)

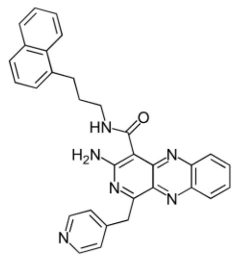

4A-19
(5.421)

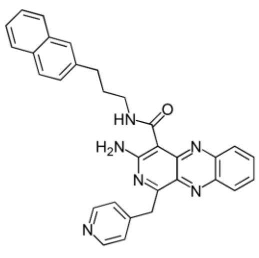

4A-20
(5.525)

Figure S4: Chemical structure of modified compounds **4A**

EXCLI Journal 2024;23:818-832 – ISSN 1611-2156
Received: March 26, 2024, accepted: April 17, 2024, published: May 27, 2024

4A

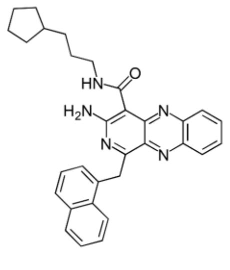

4A-21
(5.329)

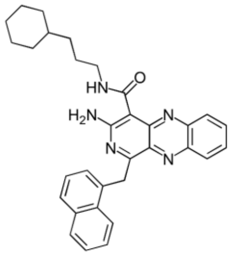

4A-22
(5.364)

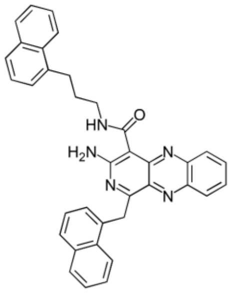

4A-23
(5.299)

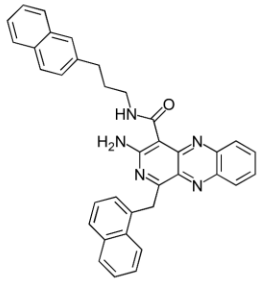

4A-24
(5.371)

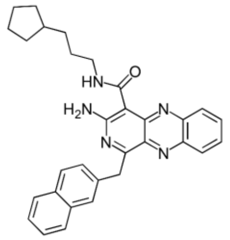

4A-25
(5.434)

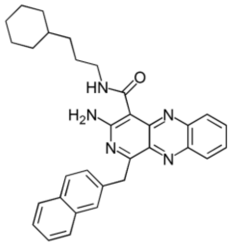

4A-26
(5.451)

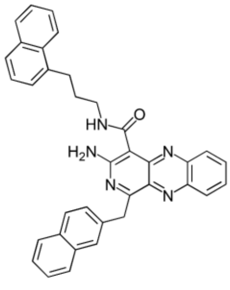

4A-27
(5.350)

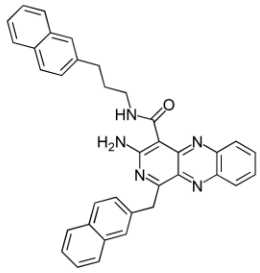

4A-28
(5.383)

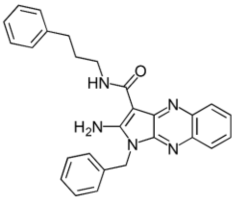

4A-29
(5.559)

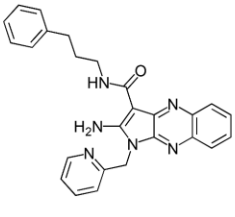

4A-30
(5.554)

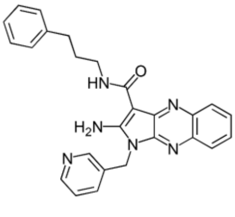

4A-31
(5.571)

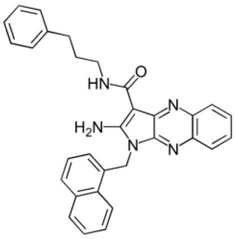

4A-32
(5.458)

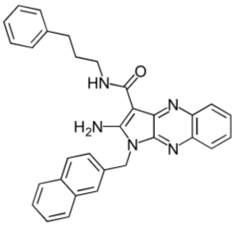

4A-33
(5.550)

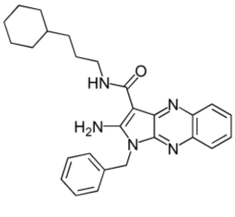

4A-34
(5.545)

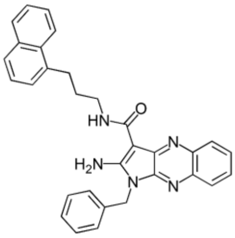

4A-35
(5.458)

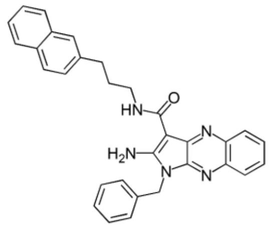

4A-36
(5.435)

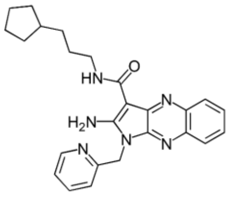

4A-37
(5.511)

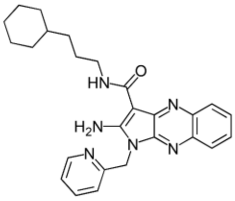

4A-38
(5.551)

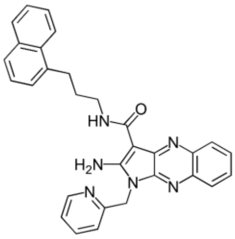

4A-39
(5.475)

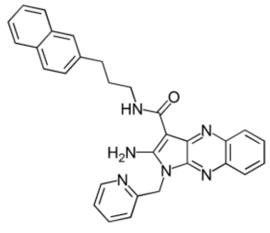

4A-40
(5.450)

Figure S4 (cont.): Chemical structure of modified compounds **4A**

EXCLI Journal 2024;23:818-832 – ISSN 1611-2156
Received: March 26, 2024, accepted: April 17, 2024, published: May 27, 2024

4A

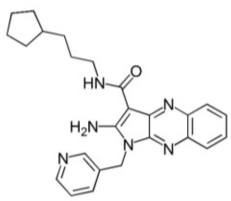

4A-41
(5.522)

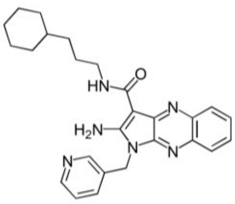

4A-42
(5.581)

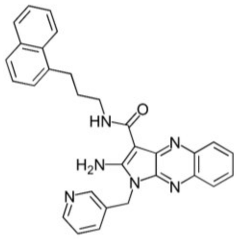

4A-43
(5.495)

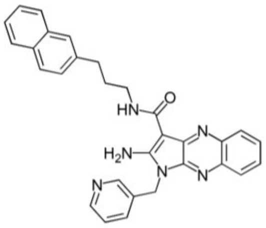

4A-44
(5.424)

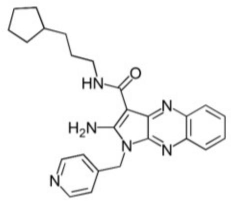

4A-45
(5.689)

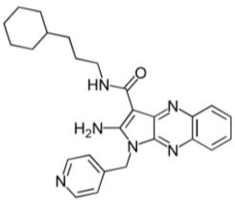

4A-46
(5.747)

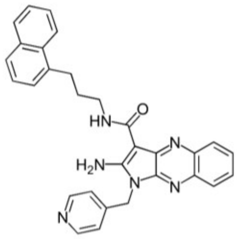

4A-47
(5.613)

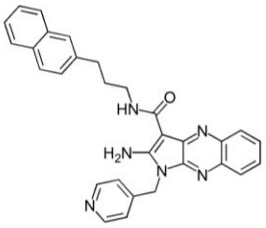

4A-48
(5.592)

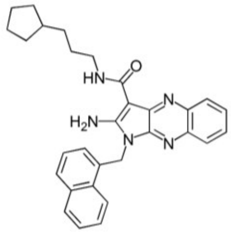

4A-49
(5.400)

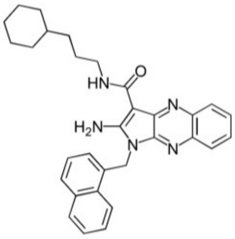

4A-50
(5.395)

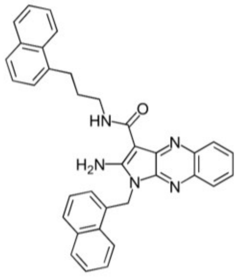

4A-51
(5.343)

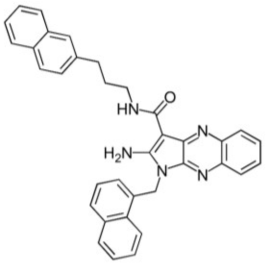

4A-52
(5.382)

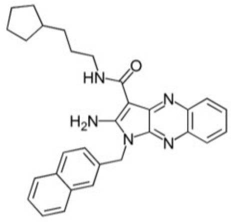

4A-53
(5.532)

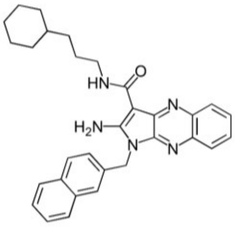

4A-54
(5.533)

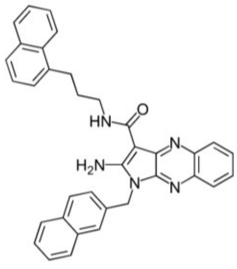

4A-55
(5.512)

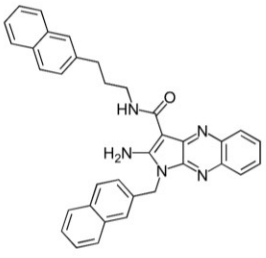

4A-56
(5.481)

Figure S4 (cont.): Chemical structure of modified compounds **4A**

5A

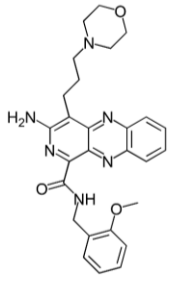

5A-1
(5.497)

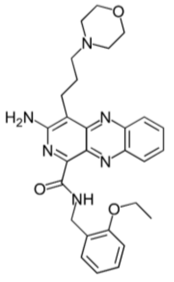

5A-2
(5.470)

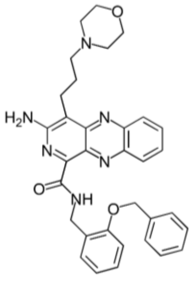

5A-3
(5.378)

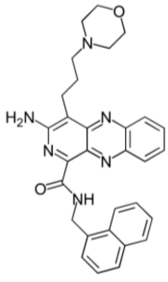

5A-4
(5.448)

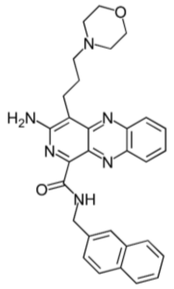

5A-5
(5.427)

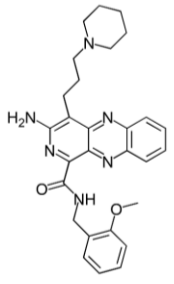

5A-6
(5.506)

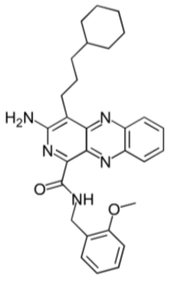

5A-7
(5.494)

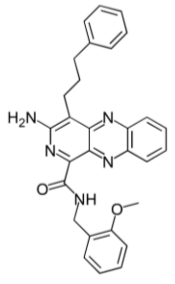

5A-8
(5.504)

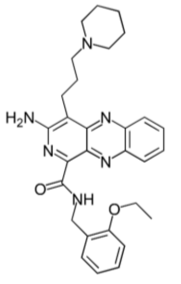

5A-9
(5.477)

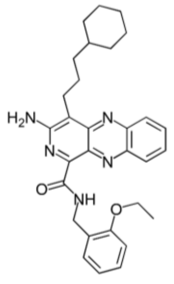

5A-10
(5.499)

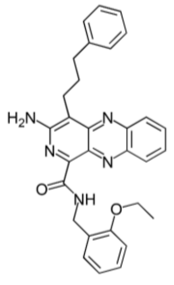

5A-11
(5.447)

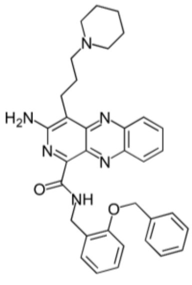

5A-12
(5.387)

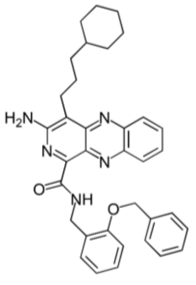

5A-13
(5.448)

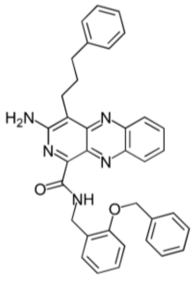

5A-14
(5.358)

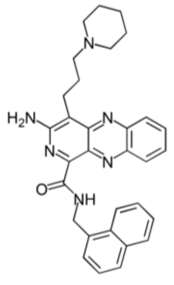

5A-15
(5.464)

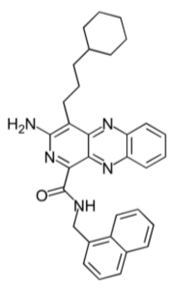

5A-16
(5.482)

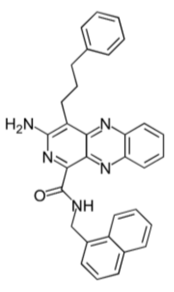

5A-17
(5.512)

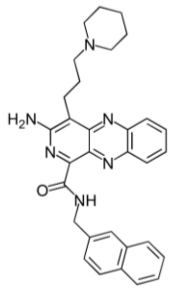

5A-18
(5.242)

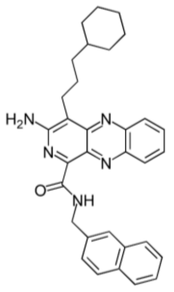

5A-19
(5.252)

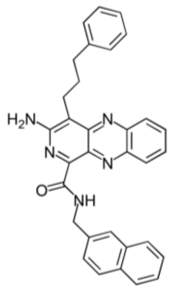

5A-20
(5.372)

Figure S5: Chemical structure of modified compounds **5A**

EXCLI Journal 2024;23:818-832 – ISSN 1611-2156
Received: March 26, 2024, accepted: April 17, 2024, published: May 27, 2024

5A

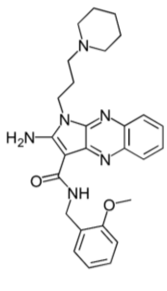

5A-21
(5.467)

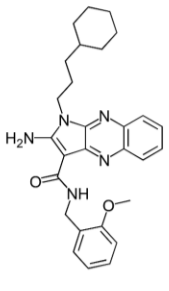

5A-22
(5.502)

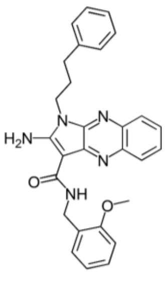

5A-23
(5.431)

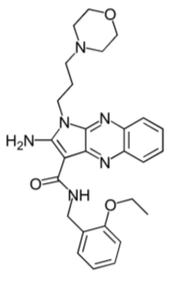

5A-24
(5.380)

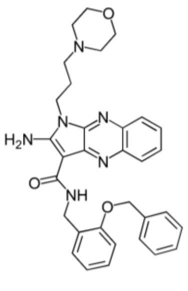

5A-25
(5.308)

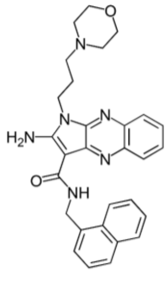

5A-26
(5.325)

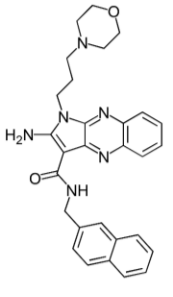

5A-27
(5.367)

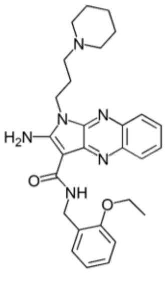

5A-28
(5.381)

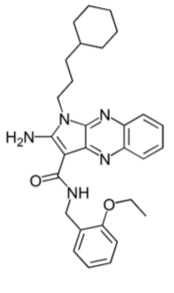

5A-29
(5.365)

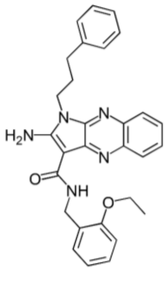

5A-30
(5.406)

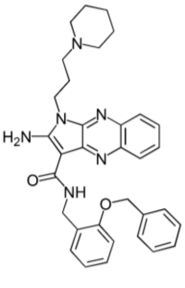

5A-31
(5.365)

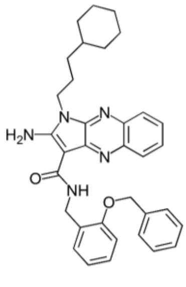

5A-32
(5.346)

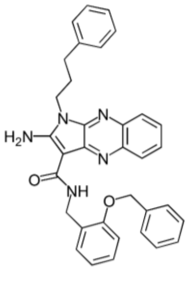

5A-33
(5.645)

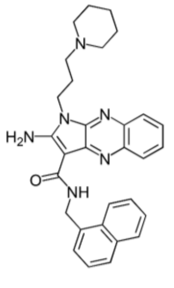

5A-34
(5.328)

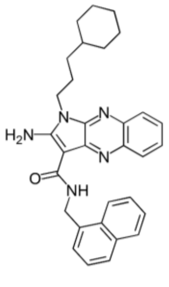

5A-35
(5.312)

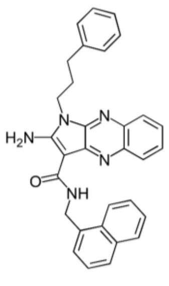

5A-36
(5.381)

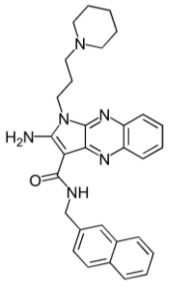

5A-37
(5.370)

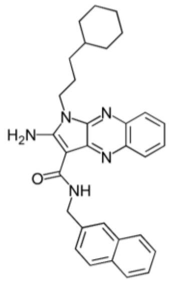

5A-38
(5.357)

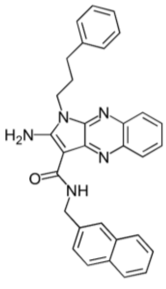

5A-39
(5.365)

Figure S5 (cont.): Chemical structure of modified compounds **5A**

EXCLI Journal 2024;23:818-832 – ISSN 1611-2156
Received: March 26, 2024, accepted: April 17, 2024, published: May 27, 2024

6A

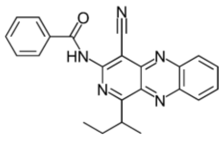
6A-1
(5.458)

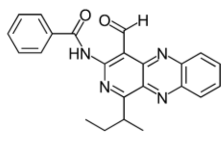
6A-2
(5.453)

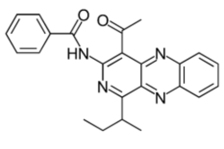
6A-3
(5.451)

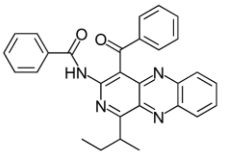
6A-4
(5.559)

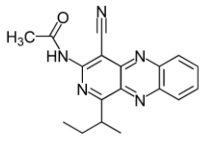
6A-5
(5.608)

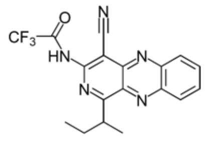
6A-6
(5.516)

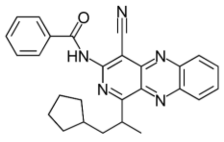
6A-7
(5.511)

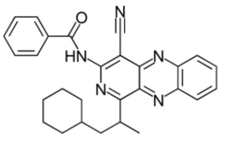
6A-8
(5.562)

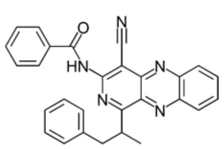
6A-9
(5.600)

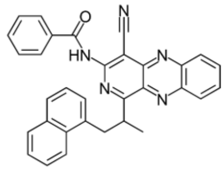
6A-10
(5.573)

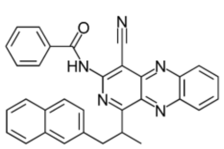
6A-11
(5.664)

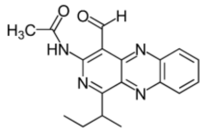
6A-12
(5.580)

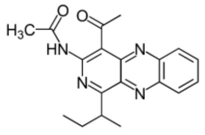
6A-13
(5.562)

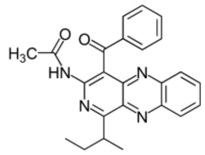
6A-14
(5.626)

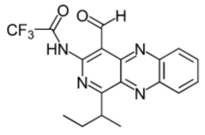
6A-15
(5.502)

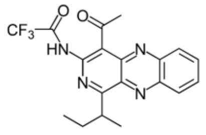
6A-16
(5.451)

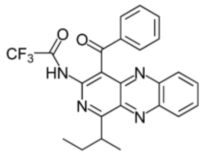
6A-17
(5.620)

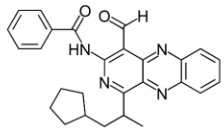
6A-18
(5.470)

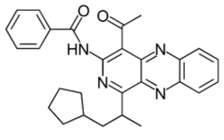
6A-19
(5.504)

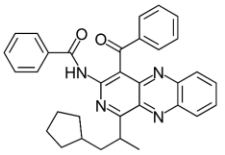
6A-20
(5.517)

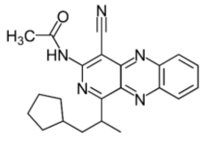
6A-21
(5.586)

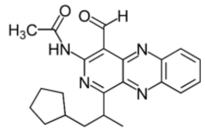
6A-22
(5.585)

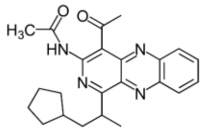
6A-23
(5.524)

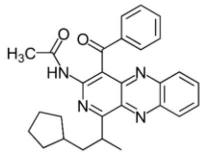
6A-24
(5.478)

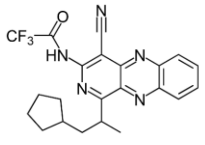
6A-25
(5.588)

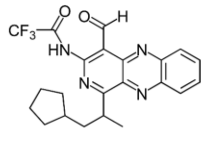
6A-26
(5.559)

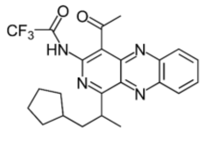
6A-27
(5.520)

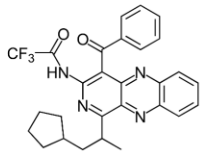
6A-28
(5.506)

Figure S6: Chemical structure of modified compounds **6A**

EXCLI Journal 2024;23:818-832 – ISSN 1611-2156
Received: March 26, 2024, accepted: April 17, 2024, published: May 27, 2024

6A

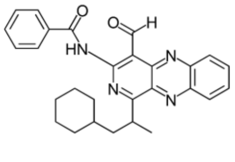

6A-29
(5.511)

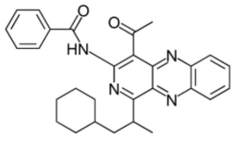

6A-30
(5.552)

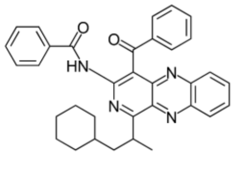

6A-31
(5.554)

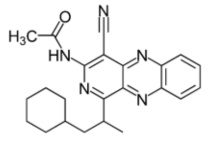

6A-32
(5.616)

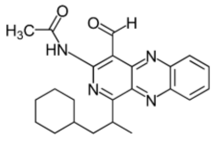

6A-33
(5.618)

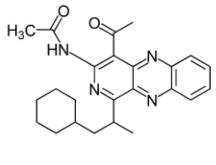

6A-34
(5.556)

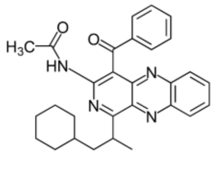

6A-35
(5.592)

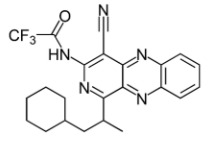

6A-36
(5.630)

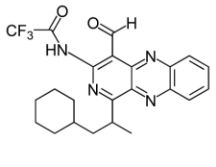

6A-37
(5.615)

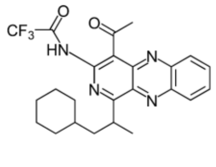

6A-38
(5.553)

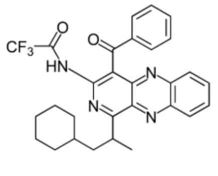

6A-39
(5.543)

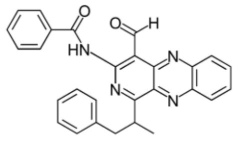

6A-40
(5.599)

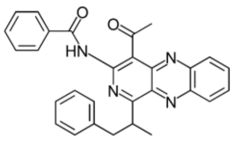

6A-41
(5.619)

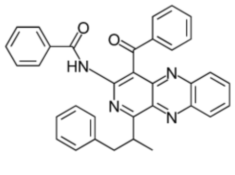

6A-42
(5.618)

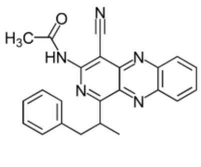

6A-43
(5.765)

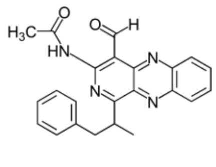

6A-44
(5.767)

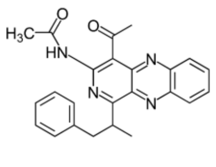

6A-45
(5.755)

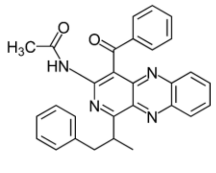

6A-46
(5.725)

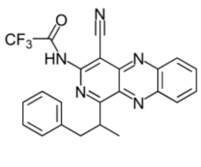

6A-47
(5.738)

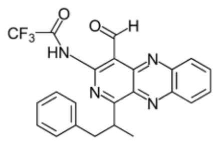

6A-48
(5.729)

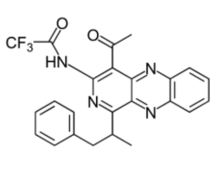

6A-49
(5.725)

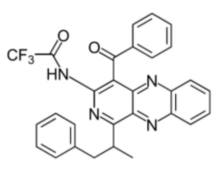

6A-50
(5.713)

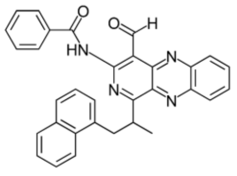

6A-51
(5.576)

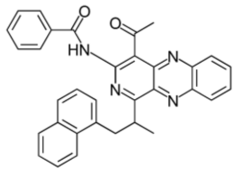

6A-52
(5.560)

Figure S6 (cont.): Chemical structure of modified compounds **6A**

EXCLI Journal 2024;23:818-832 – ISSN 1611-2156
Received: March 26, 2024, accepted: April 17, 2024, published: May 27, 2024

6A

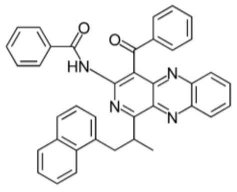

6A-53
(5.520)

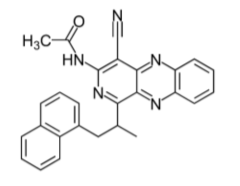

6A-54
(5.657)

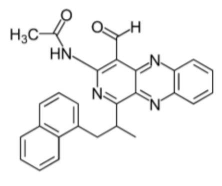

6A-55
(5.658)

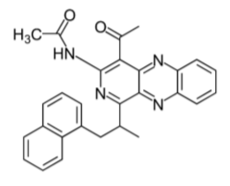

6A-56
(5.643)

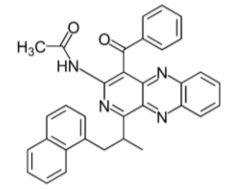

6A-57
(5.507)

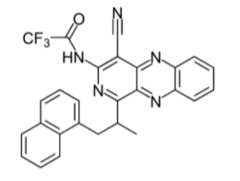

6A-58
(5.654)

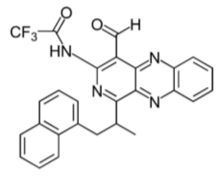

6A-59
(5.626)

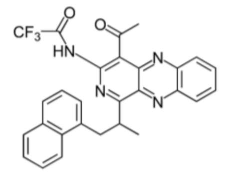

6A-60
(5.611)

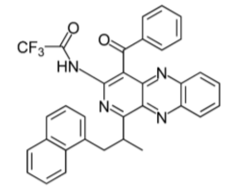

6A-61
(5.568)

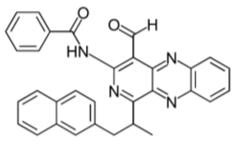

6A-62
(5.667)

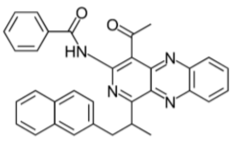

6A-63
(5.655)

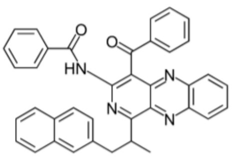

6A-64
(5.560)

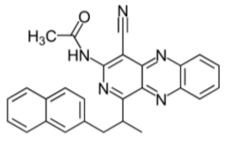

6A-65
(5.701)

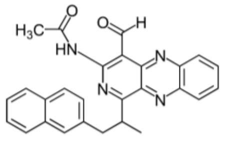

6A-66
(5.718)

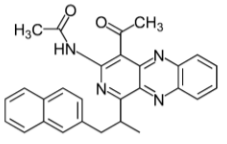

6A-67
(5.706)

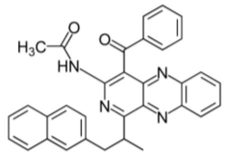

6A-68
(5.606)

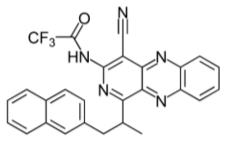

6A-69
(5.725)

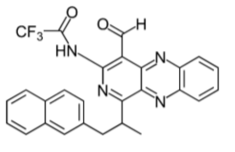

6A-70
(5.722)

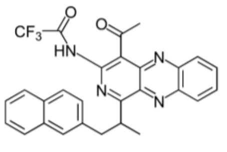

6A-71
(5.711)

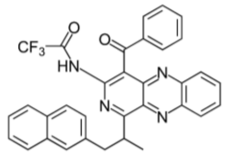

6A-72
(5.674)

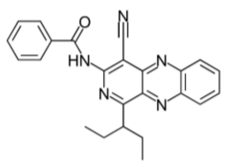

6A-73
(5.408)

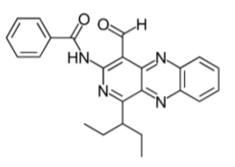

6A-74
(5.378)

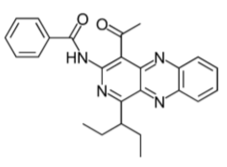

6A-75
(5.451)

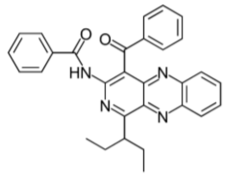

6A-76
(5.557)

Figure S6 (cont.): Chemical structure of modified compounds **6A**

EXCLI Journal 2024;23:818-832 – ISSN 1611-2156
Received: March 26, 2024, accepted: April 17, 2024, published: May 27, 2024

6A

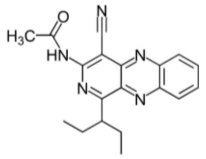

6A-77
(5.571)

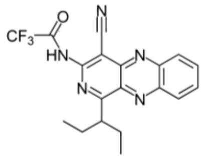

6A-78
(5.492)

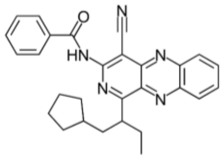

6A-79
(5.505)

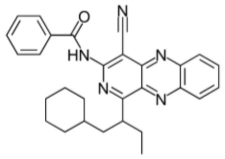

6A-80
(5.548)

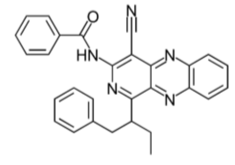

6A-81
(5.627)

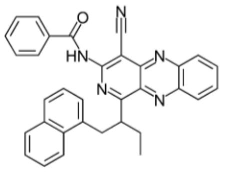

6A-82
(5.523)

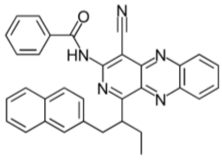

6A-83
(5.637)

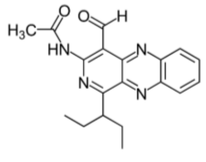

6A-84
(5.541)

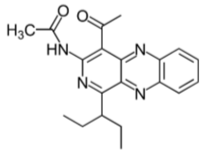

6A-85
(5.524)

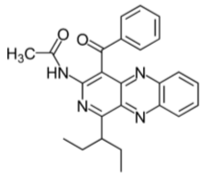

6A-86
(5.600)

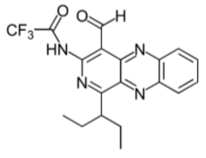

6A-87
(5.455)

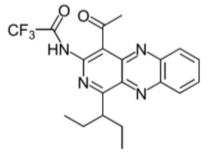

6A-88
(5.429)

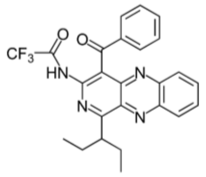

6A-89
(5.612)

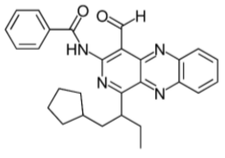

6A-90
(5.477)

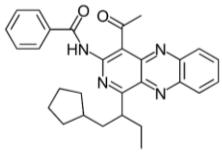

6A-91
(5.499)

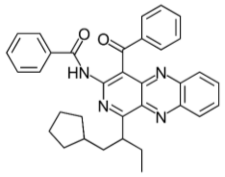

6A-92
(5.567)

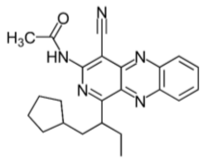

6A-93
(5.630)

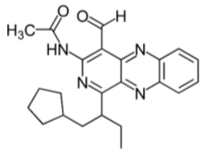

6A-94
(5.605)

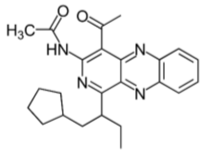

6A-95
(5.561)

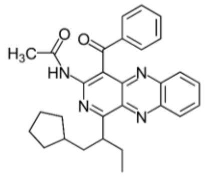

6A-96
(5.630)

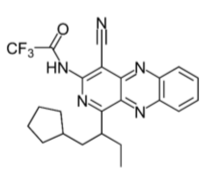

6A-97
(5.595)

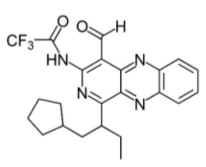

6A-98
(5.561)

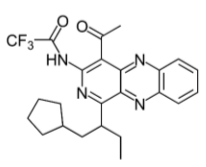

6A-99
(5.529)

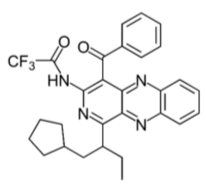

6A-100
(5.584)

Figure S6 (cont.): Chemical structure of modified compounds **6A**

EXCLI Journal 2024;23:818-832 – ISSN 1611-2156
Received: March 26, 2024, accepted: April 17, 2024, published: May 27, 2024

6A

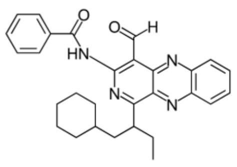

6A-101
(5.521)

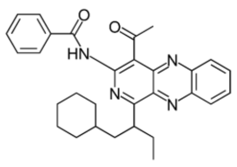

6A-102
(5.542)

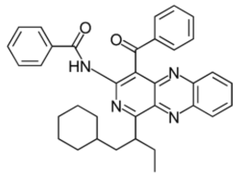

6A-103
(5.610)

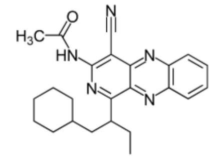

6A-104
(5.675)

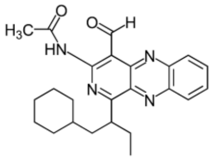

6A-105
(5.650)

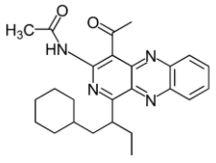

6A-106
(5.605)

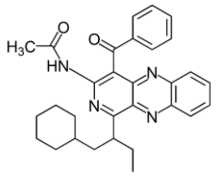

6A-107
(5.682)

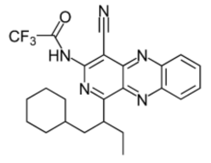

6A-108
(5.637)

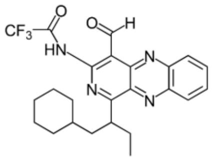

6A-109
(5.605)

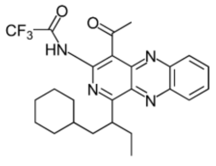

6A-110
(5.569)

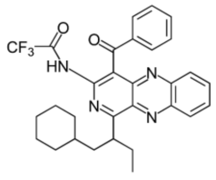

6A-111
(5.637)

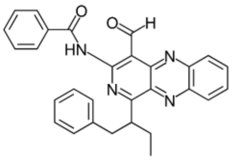

6A-112
(5.625)

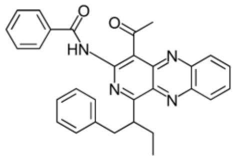

6A-113
(5.564)

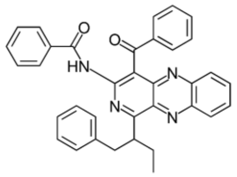

6A-114
(5.621)

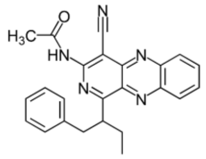

6A-115
(5.769)

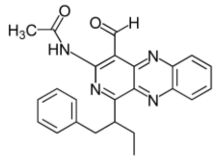

6A-116
(5.772)

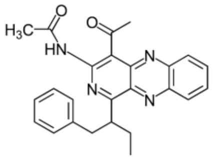

6A-117
(5.716)

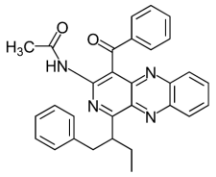

6A-118
(5.721)

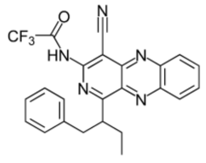

6A-119
(5.691)

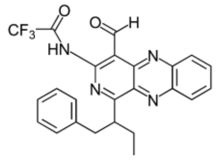

6A-120
(5.682)

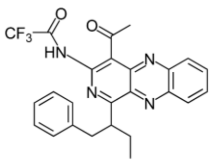

6A-121
(5.670)

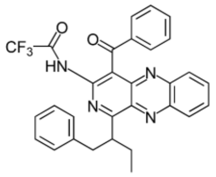

6A-122
(5.706)

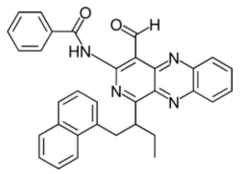

6A-123
(5.499)

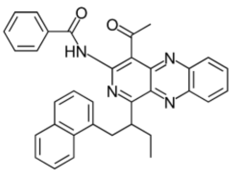

6A-124
(5.589)

Figure S6 (cont.): Chemical structure of modified compounds **6A**

EXCLI Journal 2024;23:818-832 – ISSN 1611-2156
Received: March 26, 2024, accepted: April 17, 2024, published: May 27, 2024

6A

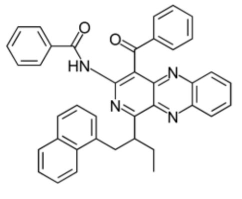	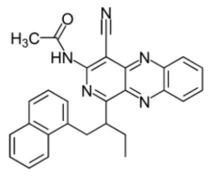	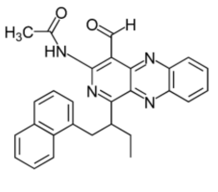	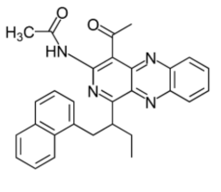
**6A-125** (5.533)	**6A-126** (5.516)	**6A-127** (5.518)	**6A-128** (5.470)
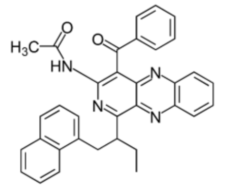	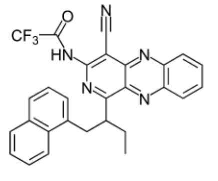	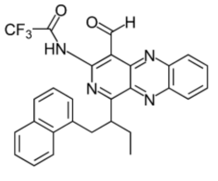	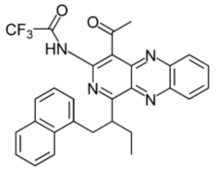
**6A-129** (5.568)	**6A-130** (5.525)	**6A-131** (5.522)	**6A-132** (5.478)
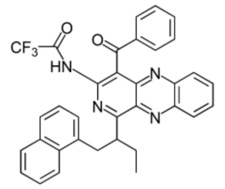	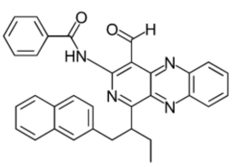	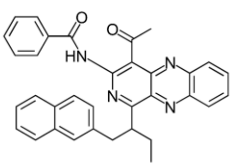	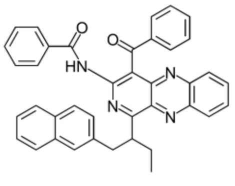
**6A-133** (5.587)	**6A-134** (5.632)	**6A-135** (5.661)	**6A-136** (5.647)
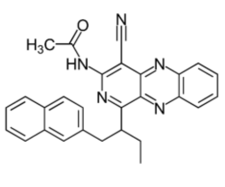	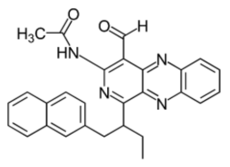	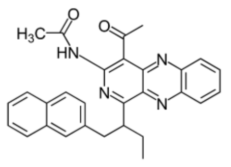	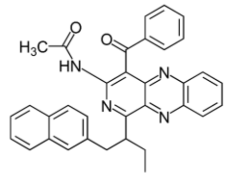
**6A-137** (5.715)	**6A-138** (5.712)	**6A-139** (5.711)	**6A-140** (5.741)
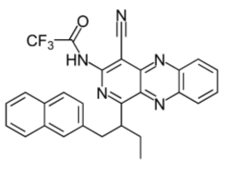	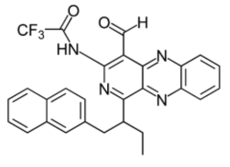	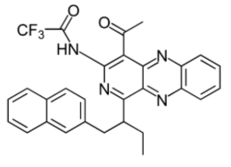	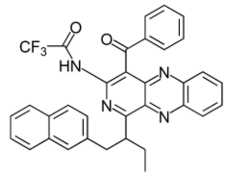
**6A-141** (5.697)	**6A-142** (5.690)	**6A-143** (5.694)	**6A-144** (5.683)
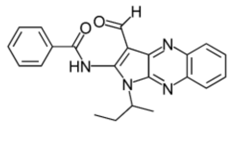	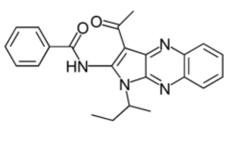	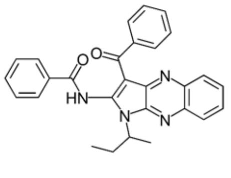	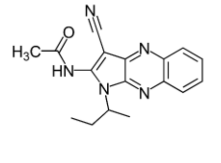
**6A-145** (5.291)	**6A-146** (5.293)	**6A-147** (5.569)	**6A-148** (5.502)

Figure S6 (cont.): Chemical structure of modified compounds **6A**

EXCLI Journal 2024;23:818-832 – ISSN 1611-2156
Received: March 26, 2024, accepted: April 17, 2024, published: May 27, 2024

6A

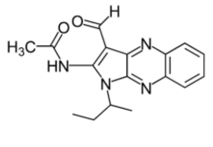

6A-149
(5.466)

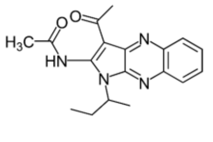

6A-150
(5.459)

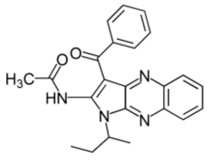

6A-151
(5.612)

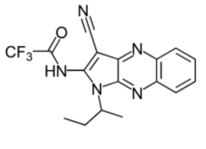

6A-152
(5.441)

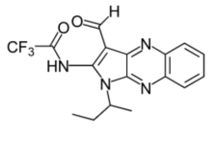

6A-153
(5.400)

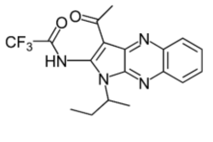

6A-154
(5.532)

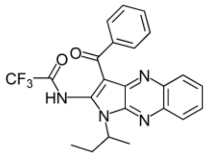

6A-155
(5.607)

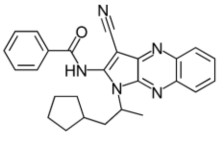

6A-156
(5.417)

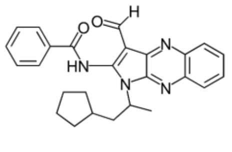

6A-157
(5.422)

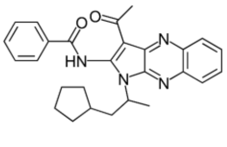

6A-158
(5.361)

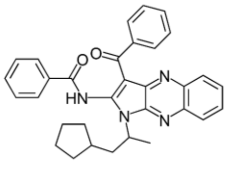

6A-159
(5.496)

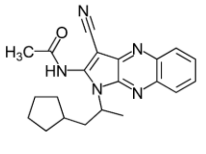

6A-160
(5.562)

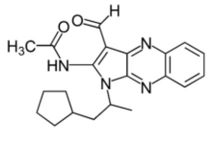

6A-161
(5.638)

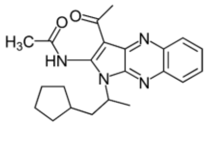

6A-162
(5.563)

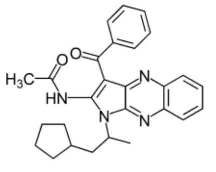

6A-163
(5.519)

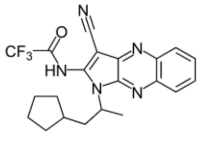

6A-164
(5.578)

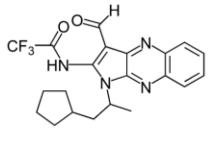

6A-165
(5.568)

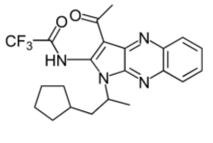

6A-166
(5.594)

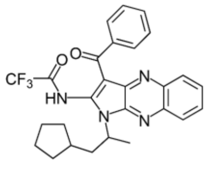

6A-167
(5.538)

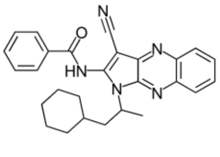

6A-168
(5.474)

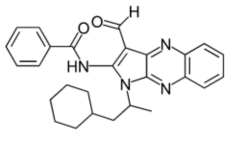

6A-169
(5.488)

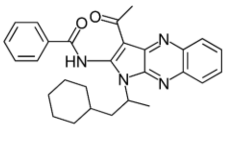

6A-170
(5.471)

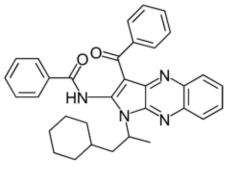

6A-171
(5.526)

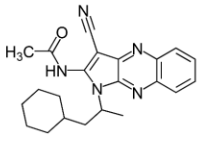

6A-172
(5.588)

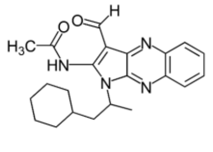

6A-173
(5.623)

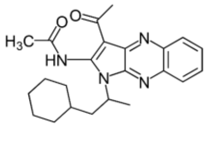

6A-174
(5.595)

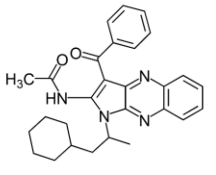

6A-175
(5.554)

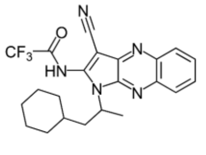

6A-176
(5.616)

Figure S6 (cont.): Chemical structure of modified compounds **6A**

EXCLI Journal 2024;23:818-832 – ISSN 1611-2156
Received: March 26, 2024, accepted: April 17, 2024, published: May 27, 2024

6A

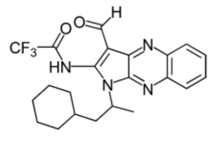

6A-177
(5.633)

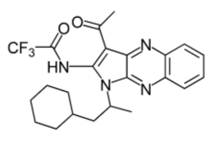

6A-178
(5.583)

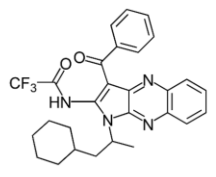

6A-179
(5.575)

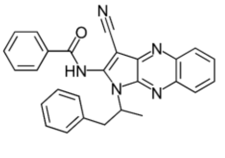

6A-180
(5.673)

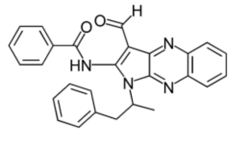

6A-181
(5.609)

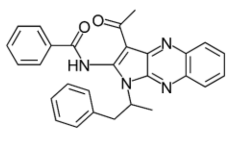

6A-182
(5.589)

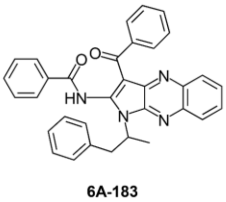

6A-183
(5.681)

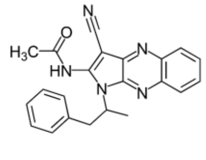

6A-184
(5.573)

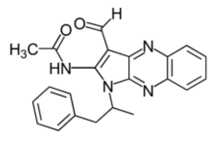

6A-185
(5.683)

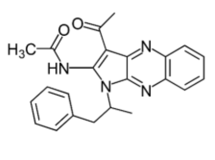

6A-186
(5.672)

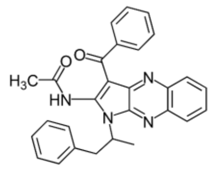

6A-187
(5.678)

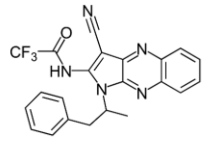

6A-188
(5.572)

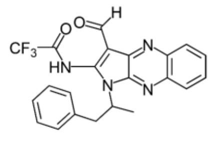

6A-189
(5.643)

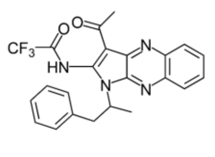

6A-190
(5.705)

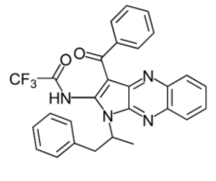

6A-191
(5.696)

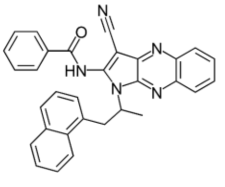

6A-192
(5.612)

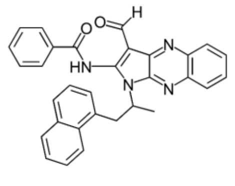

6A-193
(5.586)

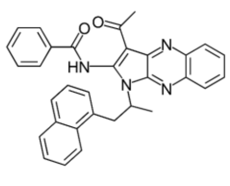

6A-194
(5.567)

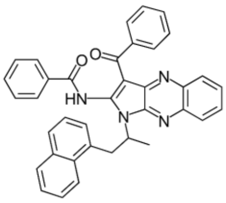

6A-195
(5.670)

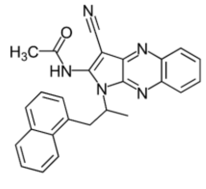

6A-196
(5.404)

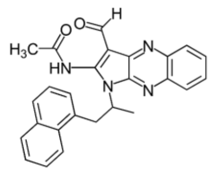

6A-197
(5.522)

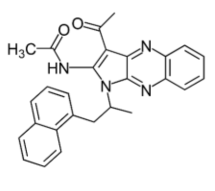

6A-198
(5.452)

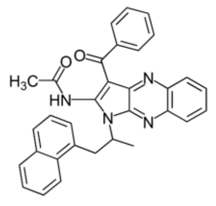

6A-199
(5.576)

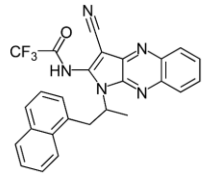

6A-200
(5.549)

Figure S6 (cont.): Chemical structure of modified compounds **6A**

EXCLI Journal 2024;23:818-832 – ISSN 1611-2156
Received: March 26, 2024, accepted: April 17, 2024, published: May 27, 2024

6A

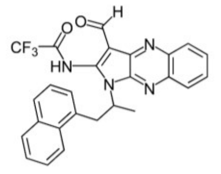

6A-201
(5.594)

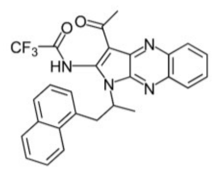

6A-202
(5.569)

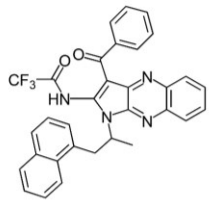

6A-203
(5.579)

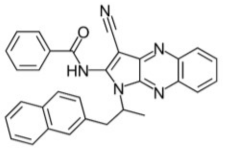

6A-204
(5.685)

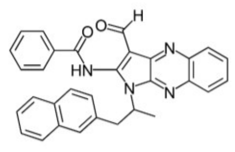

6A-205
(5.580)

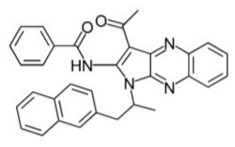

6A-206
(5.490)

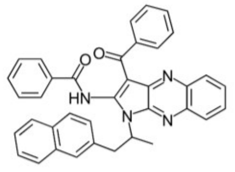

6A-207
(5.560)

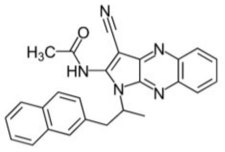

6A-208
(5.525)

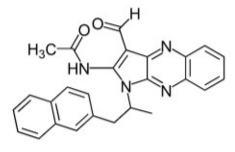

6A-209
(5.532)

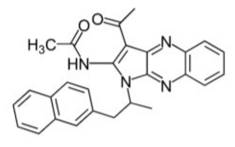

6A-210
(5.666)

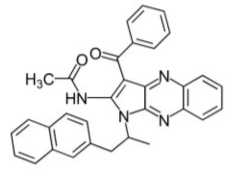

6A-211
(5.661)

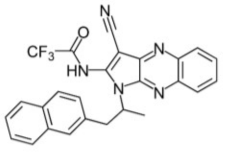

6A-212
(5.644)

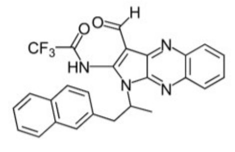

6A-213
(5.617)

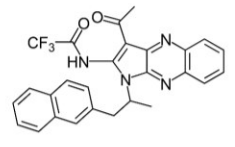

6A-214
(5.631)

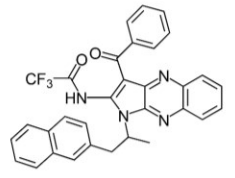

6A-215
(5.617)

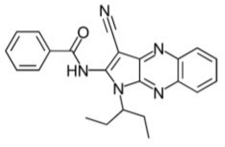

6A-216
(5.283)

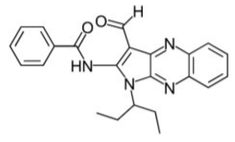

6A-217
(5.279)

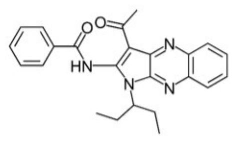

6A-218
(5.262)

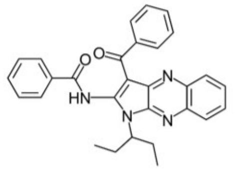

6A-219
(5.504)

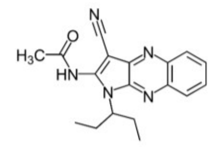

6A-220
(5.519)

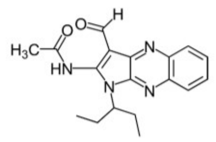

6A-221
(5.489)

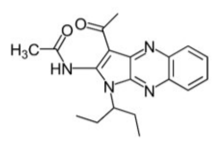

6A-222
(5.478)

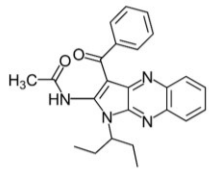

6A-223
(5.552)

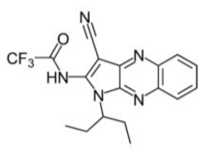

6A-224
(5.463)

Figure S6 (cont.): Chemical structure of modified compounds **6A**

6A

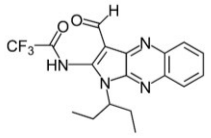

6A-225
(5.424)

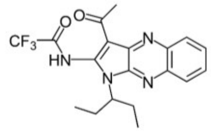

6A-226
(5.400)

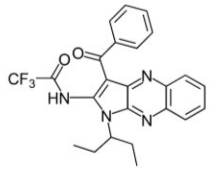

6A-227
(5.571)

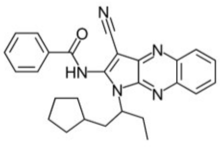

6A-228
(5.460)

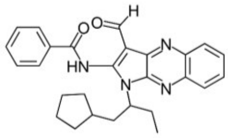

6A-229
(5.444)

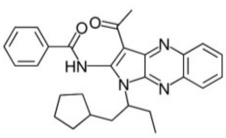

6A-230
(5.370)

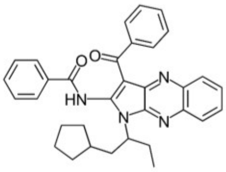

6A-231
(5.361)

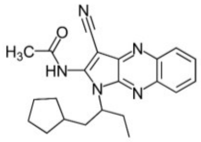

6A-232
(5.564)

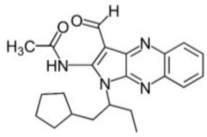

6A-233
(5.542)

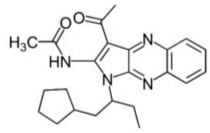

6A-234
(5.493)

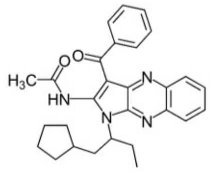

6A-235
(5.440)

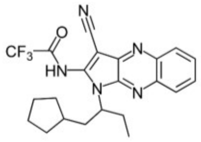

6A-236
(5.559)

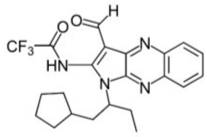

6A-237
(5.555)

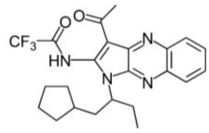

6A-238
(5.506)

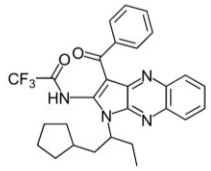

6A-239
(5.513)

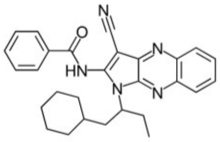

6A-240
(5.457)

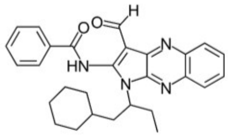

6A-241
(5.463)

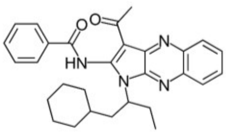

6A-242
(5.414)

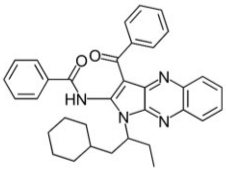

6A-243
(5.384)

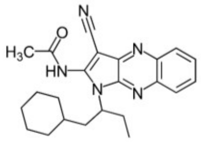

6A-244
(5.550)

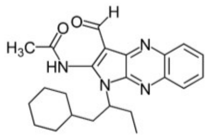

6A-245
(5.509)

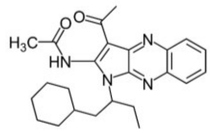

6A-246
(5.491)

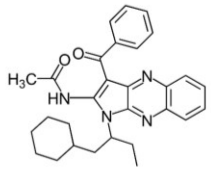

6A-247
(5.415)

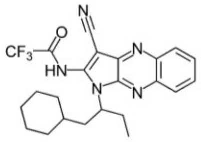

6A-248
(5.577)

Figure S6 (cont.): Chemical structure of modified compounds **6A**

EXCLI Journal 2024;23:818-832 – ISSN 1611-2156
Received: March 26, 2024, accepted: April 17, 2024, published: May 27, 2024

6A

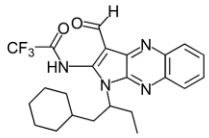
6A-249
(5.572)

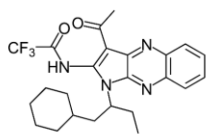
6A-250
(5.498)

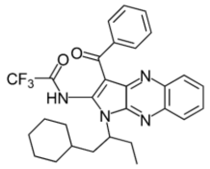
6A-251
(5.505)

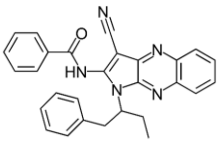
6A-252
(5.501)

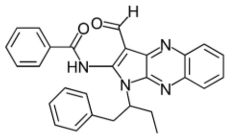
6A-253
(5.482)

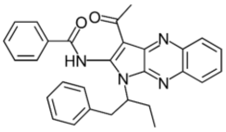
6A-254
(5.457)

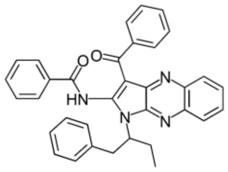
6A-255
(5.403)

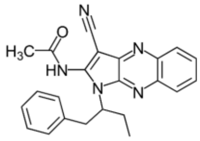
6A-256
(5.531)

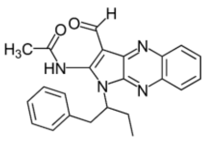
6A-257
(5.522)

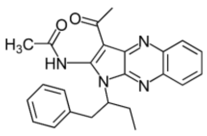
6A-258
(5.500)

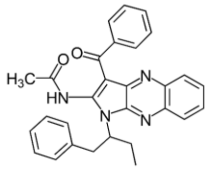
6A-259
(5.421)

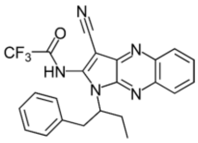
6A-260
(5.572)

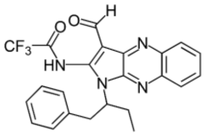
6A-261
(5.543)

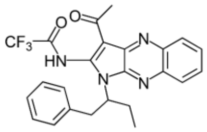
6A-262
(5.518)

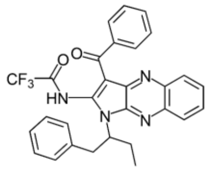
6A-263
(5.493)

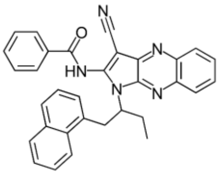
6A-264
(5.548)

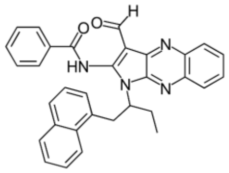
6A-265
(5.554)

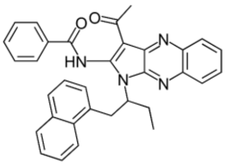
6A-266
(5.553)

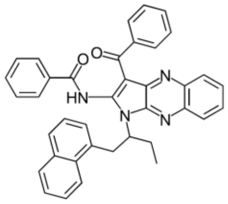
6A-267
(5.499)

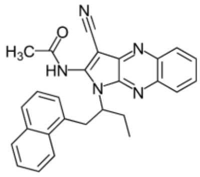
6A-268
(5.529)

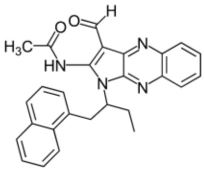
6A-269
(5.544)

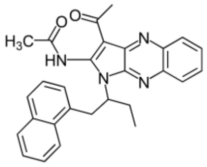
6A-270
(5.531)

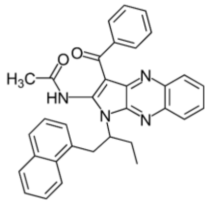
6A-271
(5.410)

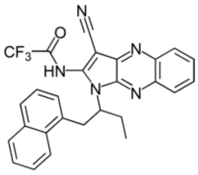
6A-272
(5.563)

Figure S6 (cont.): Chemical structure of modified compounds **6A**

6A

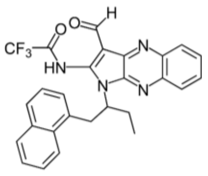

6A-273
(5.559)

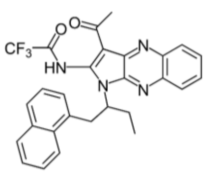

6A-274
(5.559)

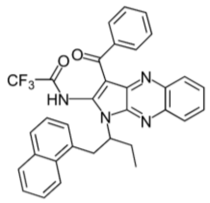

6A-275
(5.538)

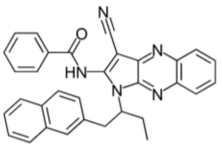

6A-276
(5.447)

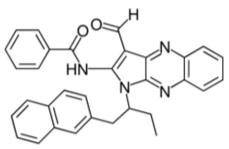

6A-277
(5.452)

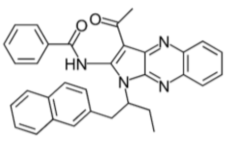

6A-278
(5.403)

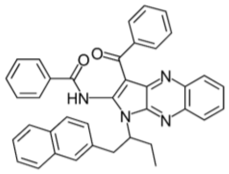

6A-279
(5.348)

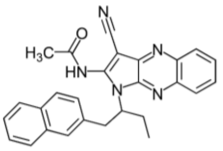

6A-280
(5.428)

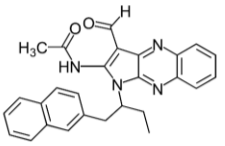

6A-281
(5.444)

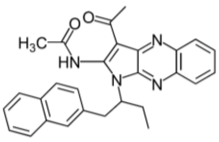

6A-282
(5.413)

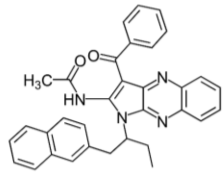

6A-283
(5.268)

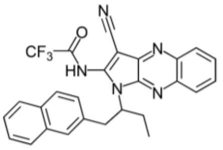

6A-284
(5.537)

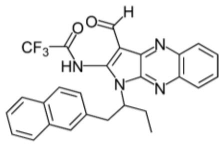

6A-285
(5.534)

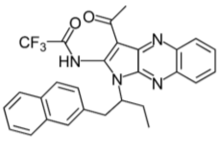

6A-286
(5.484)

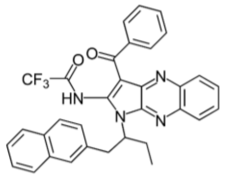

6A-287
(5.393)

Figure S6 (cont.): Chemical structure of modified compounds **6A**

EXCLI Journal 2024;23:818-832 – ISSN 1611-2156
Received: March 26, 2024, accepted: April 17, 2024, published: May 27, 2024

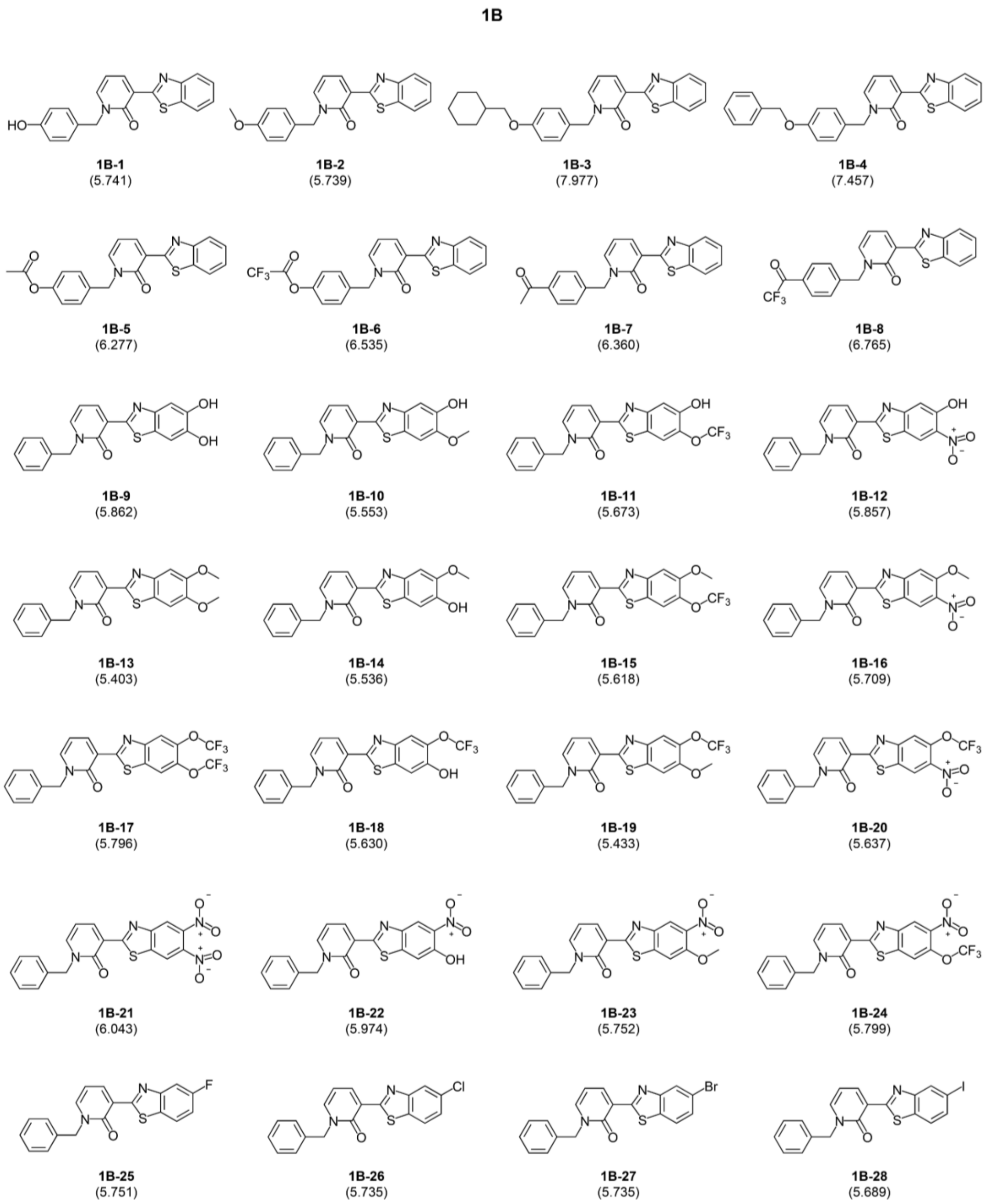

Figure S7: Chemical structure of modified compounds **1B**

EXCLI Journal 2024;23:818-832 – ISSN 1611-2156
Received: March 26, 2024, accepted: April 17, 2024, published: May 27, 2024

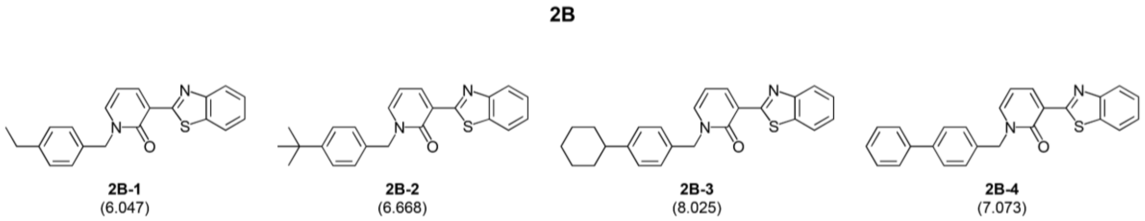

Figure S8: Chemical structure of modified compounds **2B**

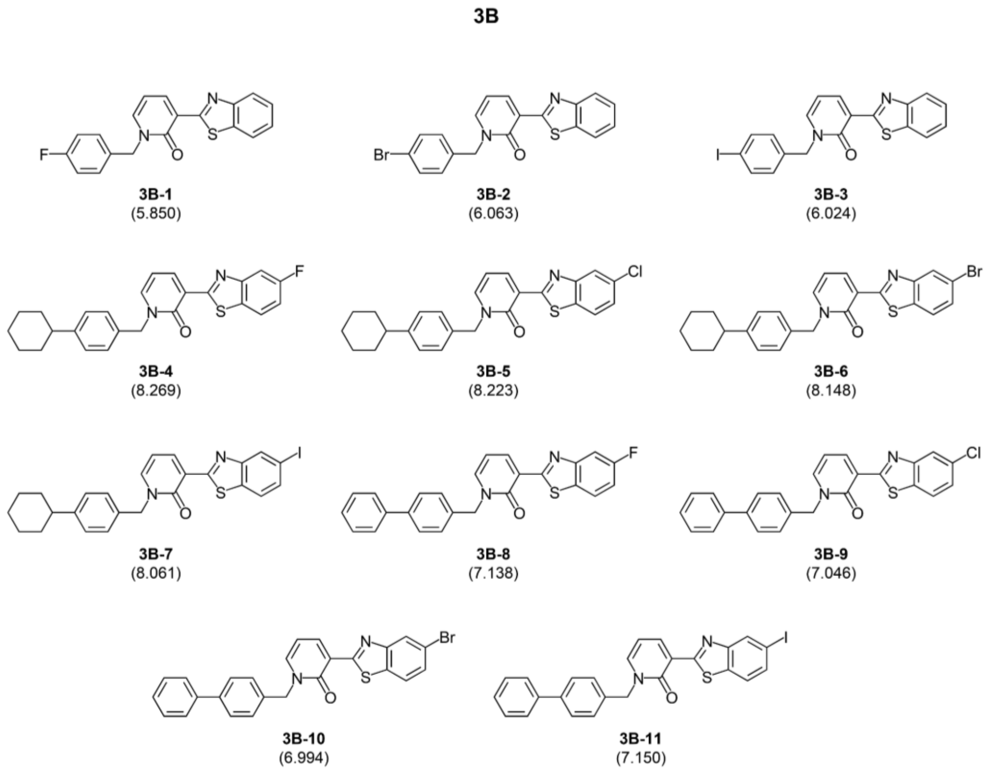

Figure S9: Chemical structure of modified compounds **3B**

EXCLI Journal 2024;23:818-832 – ISSN 1611-2156
Received: March 26, 2024, accepted: April 17, 2024, published: May 27, 2024

4B

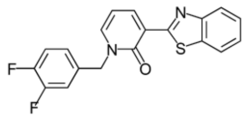
4B-1
(6.118)

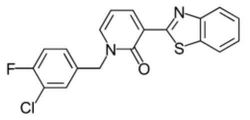
4B-2
(6.020)

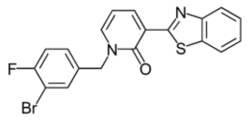
4B-3
(5.969)

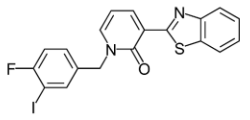
4B-4
(6.015)

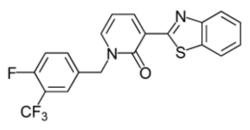
4B-5
(5.965)

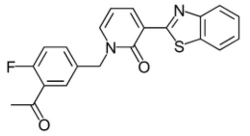
4B-6
(5.732)

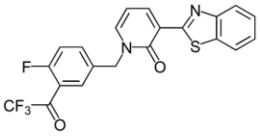
4B-7
(5.989)

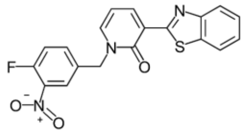
4B-8
(6.061)

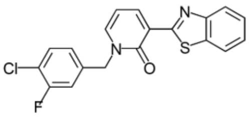
4B-9
(6.234)

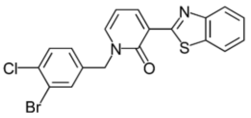
4B-10
(6.030)

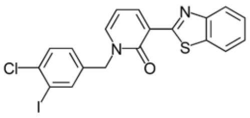
4B-11
(5.928)

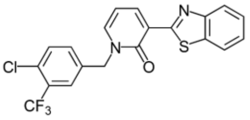
4B-12
(6.052)

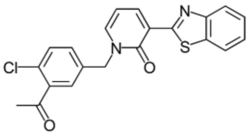
4B-13
(6.177)

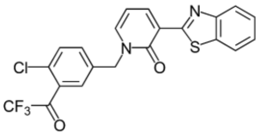
4B-14
(6.408)

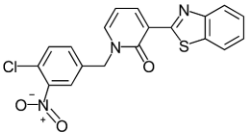
4B-15
(6.027)

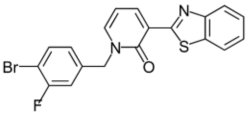
4B-16
(6.278)

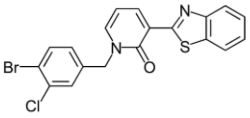
4B-17
(6.177)

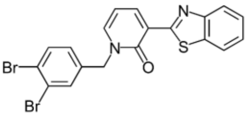
4B-18
(6.053)

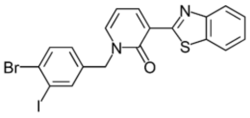
4B-19
(5.939)

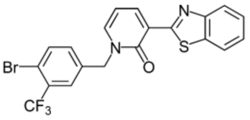
4B-20
(6.098)

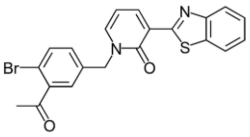
4B-21
(6.207)

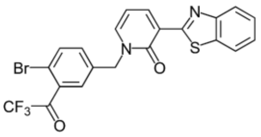
4B-22
(6.116)

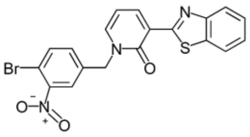
4B-23
(6.397)

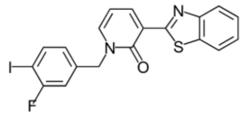
4B-24
(6.213)

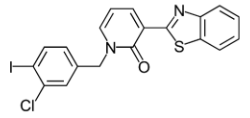
4B-25
(6.091)

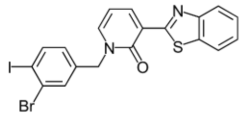
4B-26
(5.581)

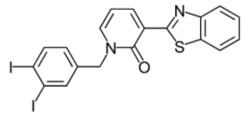
4B-27
(5.871)

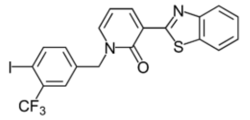
4B-28
(6.112)

Figure S10: Chemical structure of modified compounds **4B**

EXCLI Journal 2024;23:818-832 – ISSN 1611-2156
Received: March 26, 2024, accepted: April 17, 2024, published: May 27, 2024

4B

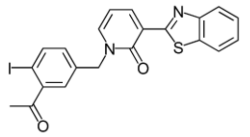
4B-29
(6.213)

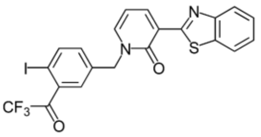
4B-30
(6.608)

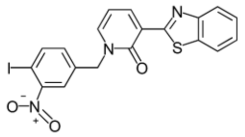
4B-31
(6.139)

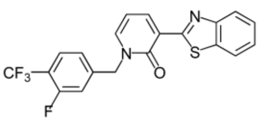
4B-32
(6.020)

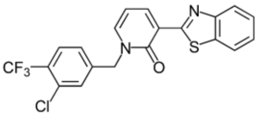
4B-33
(5.918)

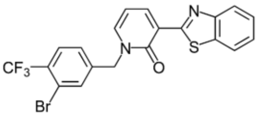
4B-34
(5.863)

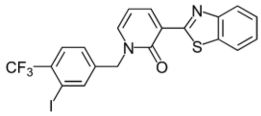
4B-35
(5.792)

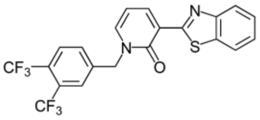
4B-36
(6.026)

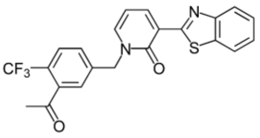
4B-37
(5.957)

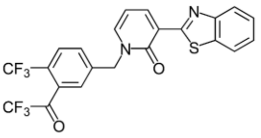
4B-38
(5.751)

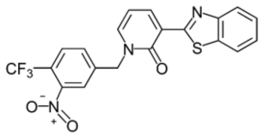
4B-39
(5.942)

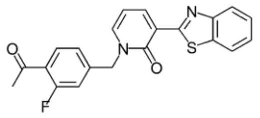
4B-40
(6.322)

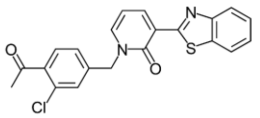
4B-41
(5.999)

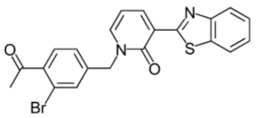
4B-42
(5.906)

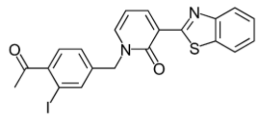
4B-43
(5.850)

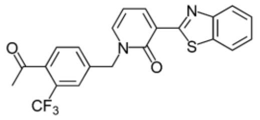
4B-44
(5.970)

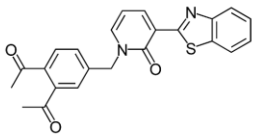
4B-45
(6.193)

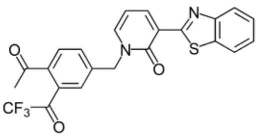
4B-46
(6.287)

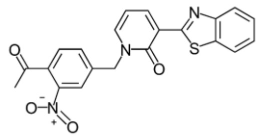
4B-47
(6.183)

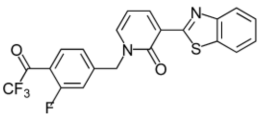
4B-48
(6.795)

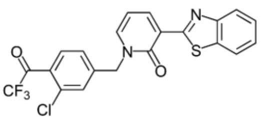
4B-49
(6.375)

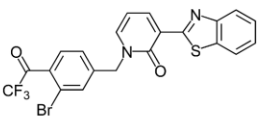
4B-50
(6.294)

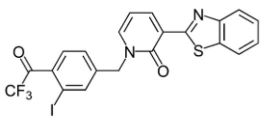
4B-51
(6.545)

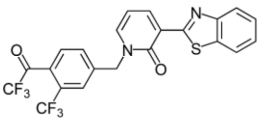
4B-52
(6.768)

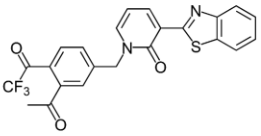
4B-53
(6.789)

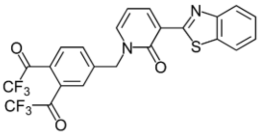
4B-54
(6.620)

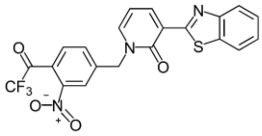
4B-55
(6.275)

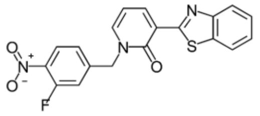
4B-56
(6.184)

Figure S10 (cont.): Chemical structure of modified compounds **4B**

EXCLI Journal 2024;23:818-832 – ISSN 1611-2156
Received: March 26, 2024, accepted: April 17, 2024, published: May 27, 2024

4B

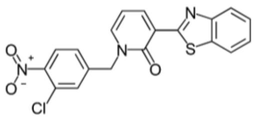

4B-57
(5.985)

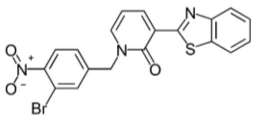

4B-58
(6.263)

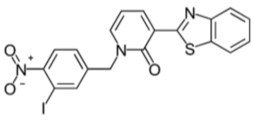

4B-59
(5.786)

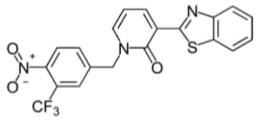

4B-60
(6.080)

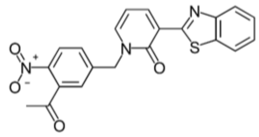

4B-61
(6.261)

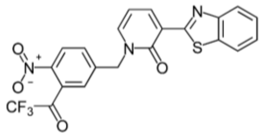

4B-62
(6.292)

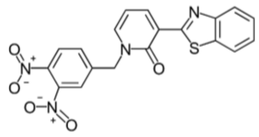

4B-63
(5.973)

Figure S10 (cont.): Chemical structure of modified compounds **4B**

5B

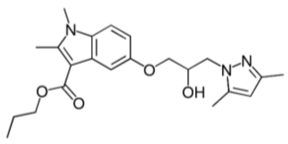

5B-1
(6.596)

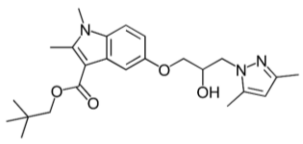

5B-2
(7.073)

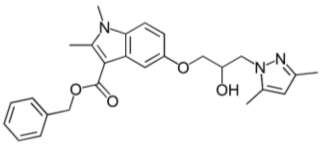

5B-3
(7.304)

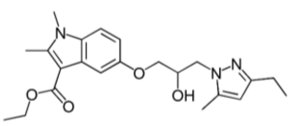

5B-4
(5.794)

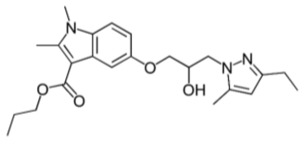

5B-5
(7.896)

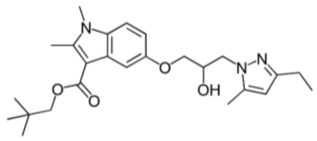

5B-6
(6.302)

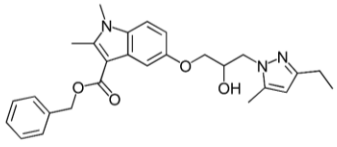

5B-7
(8.728)

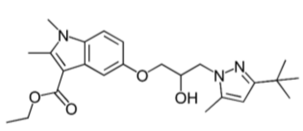

5B-8
(7.007)

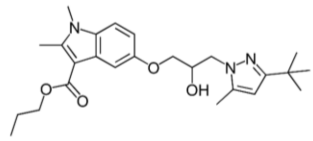

5B-9
(8.784)

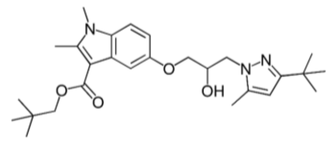

5B-10
(7.682)

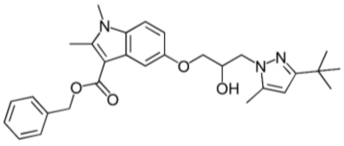

5B-11
(8.415)

Figure S11: Chemical structure of modified compounds **5B**
